# Correction: Emergence of corpse cremation during the Pre-Pottery Neolithic of the Southern Levant: A multidisciplinary study of a pyre-pit burial

**DOI:** 10.1371/journal.pone.0246488

**Published:** 2021-01-28

**Authors:** The PLOS ONE Staff

## Notice of republication

This article was republished on November 5, 2020, to correct errors throughout the article that were introduced during the typesetting process. The publisher apologizes for the errors. Please download this article again to view the correct version. The originally published, uncorrected article and the republished, corrected articles are provided here for reference.

## Supporting information

S1 File Originally published, uncorrected article. (PDF)

S2 File Republished, corrected article. (PDF)
