# Supplementary material for: Emergence of corpse cremation during the Pre-Pottery Neolithic of the Southern Levant: A multidisciplinary study of a pyre-pit burial
Source: PLoS One. 2020 Aug 12;15(8):e0235386. doi: 10.1371/journal.pone.0235386 (PMC7423105; doi:10.1371/journal.pone.0235386)
Supplement: S1 Fig — https://doi.org/10.7794/p6w6-f483. (PDF) [file pone.0235386.s001.pdf]

Beisamoun
Locus 338

catalogues 2791, 2804

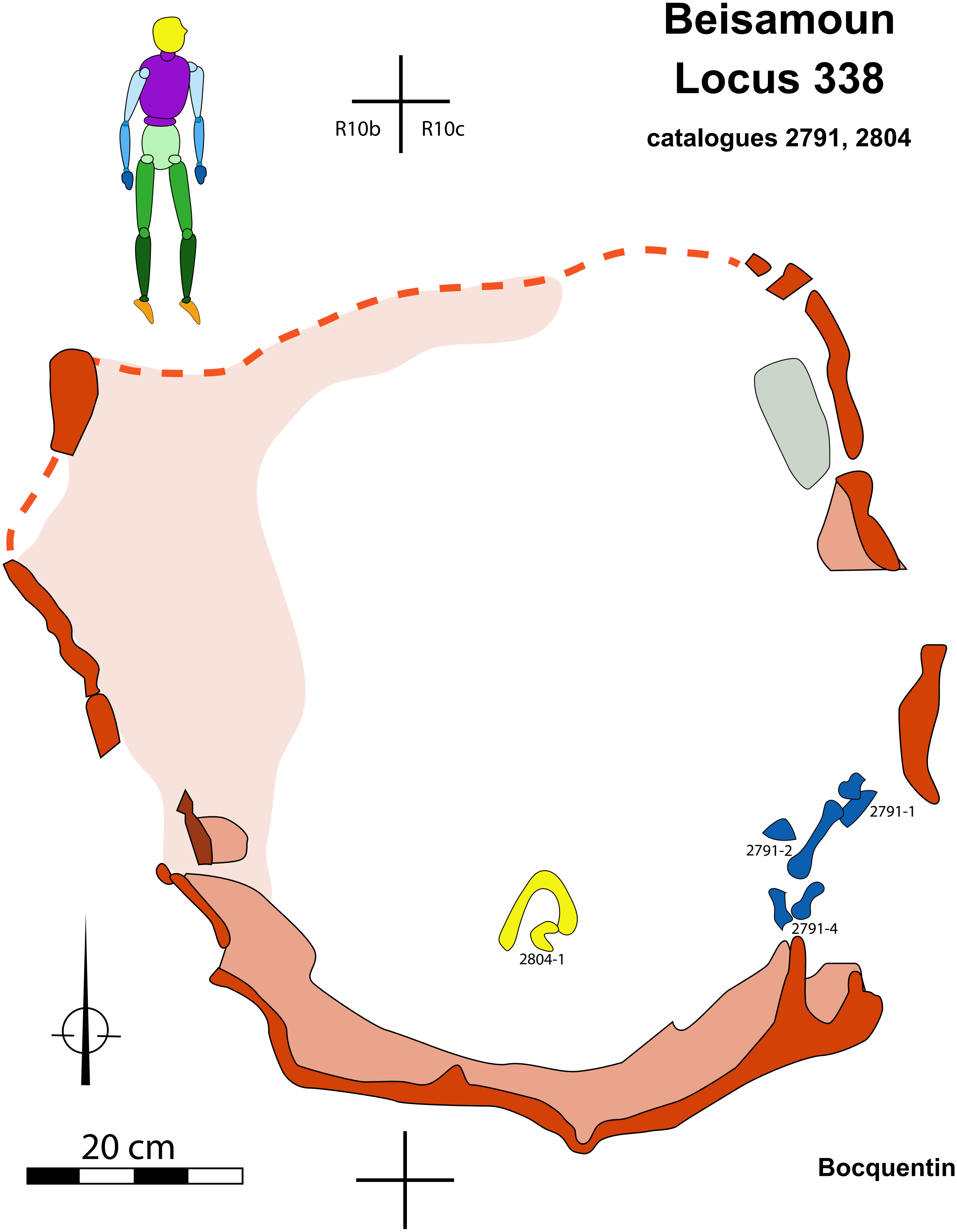

Bocquentin et al.,

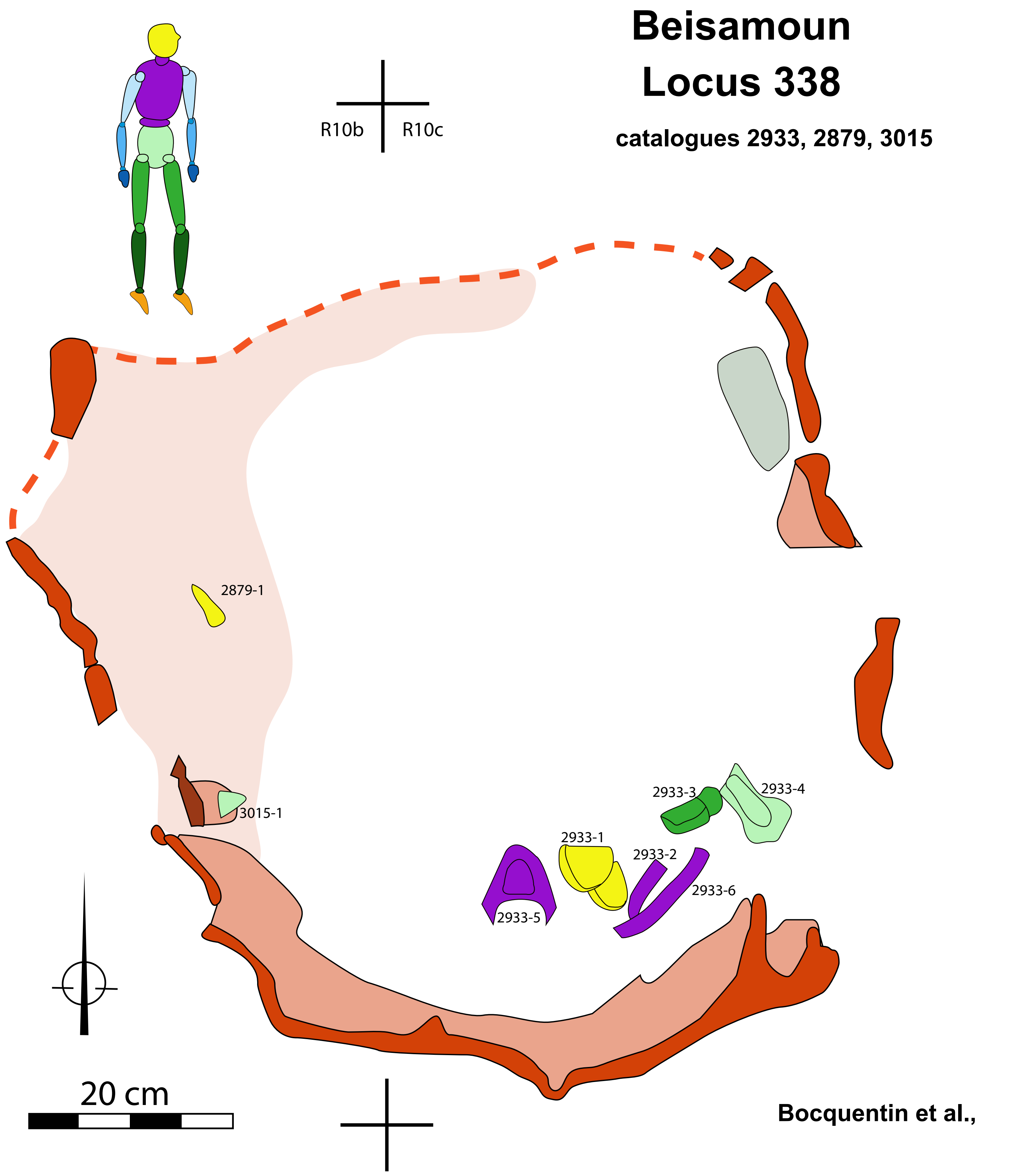
Beisamoun
Locus 338
catalogues 2933, 2879, 3015
R10b
R10c
2879-1
3015-1
2933-3
2933-4
2933-1
2933-2
2933-6
2933-5
20 cm
Bocquentin et al.,

Beisamoun
Locus 338

catalogues 3051, 3102

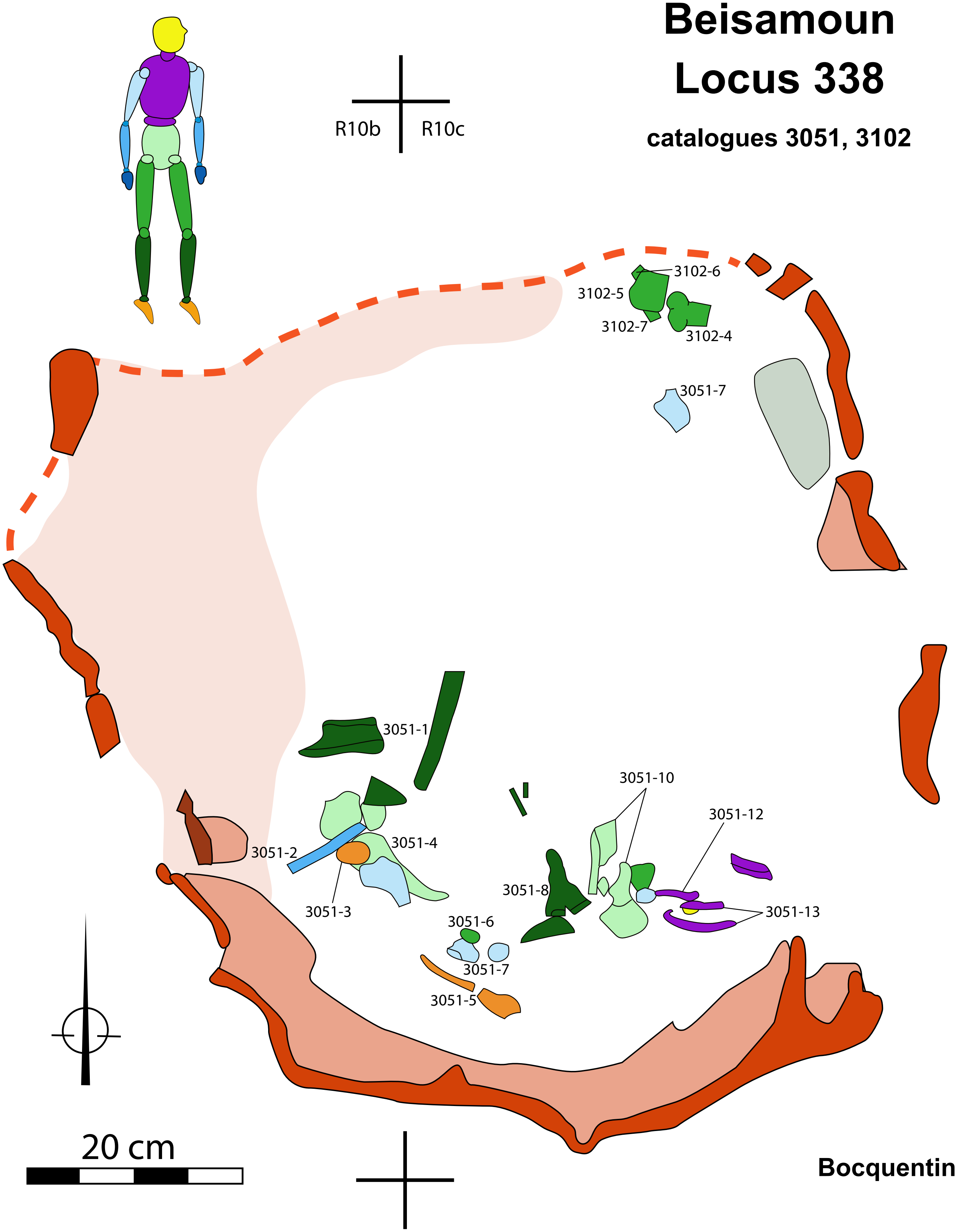

Bocquentin et al.,

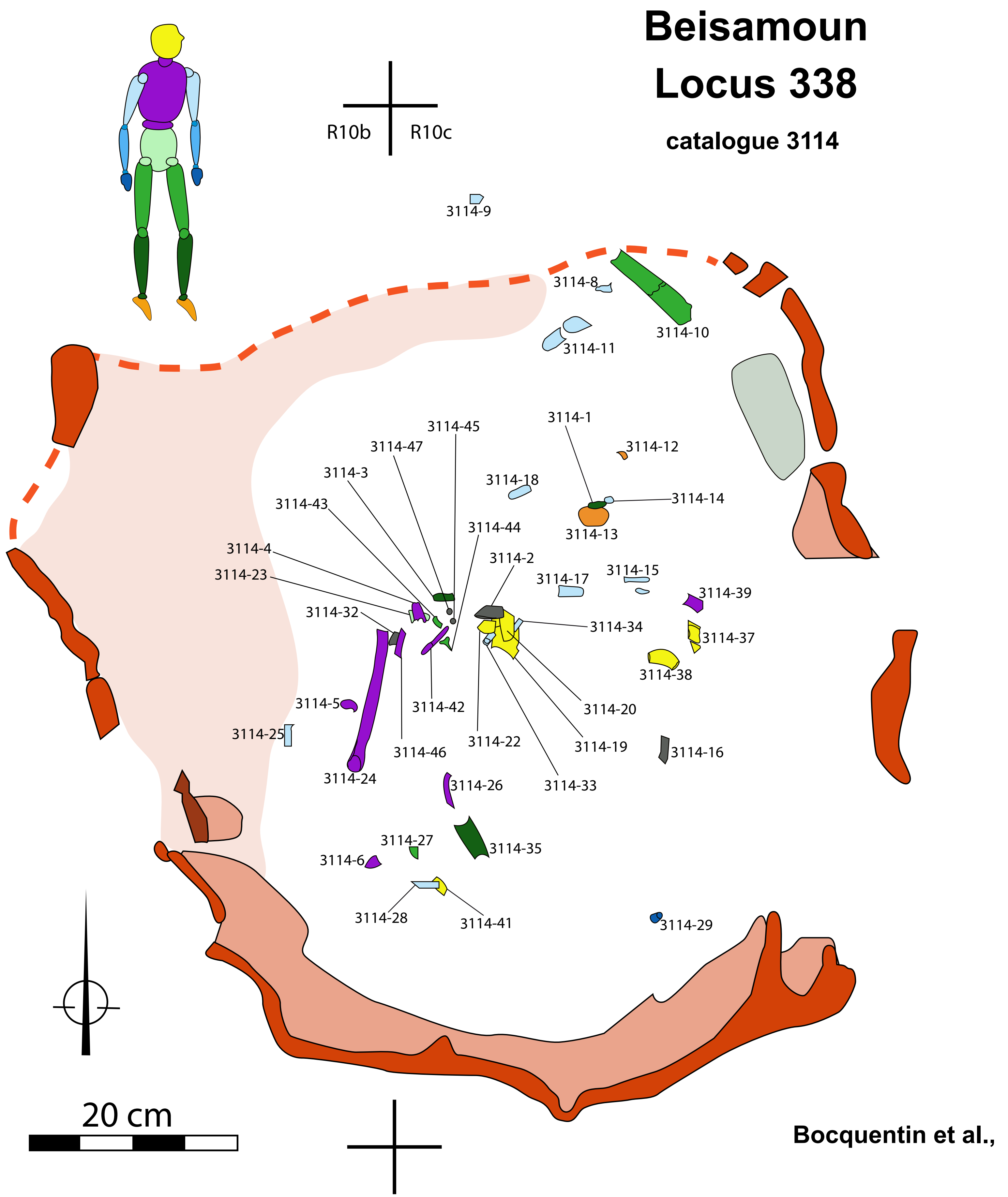
Beisamoun
Locus 338
catalogue 3114
R10b
R10c
3114-9
3114-8
3114-10
3114-11
3114-1
3114-12
3114-45
3114-47
3114-3
3114-18
3114-14
3114-43
3114-44
3114-13
3114-4
3114-2
3114-23
3114-17
3114-15
3114-39
3114-32
3114-34
3114-37
3114-38
3114-5
3114-42
3114-20
3114-25
3114-46
3114-22
3114-19
3114-16
3114-24
3114-26
3114-33
3114-27
3114-35
3114-6
3114-28
3114-41
3114-29
20 cm
Bocquentin et al.,

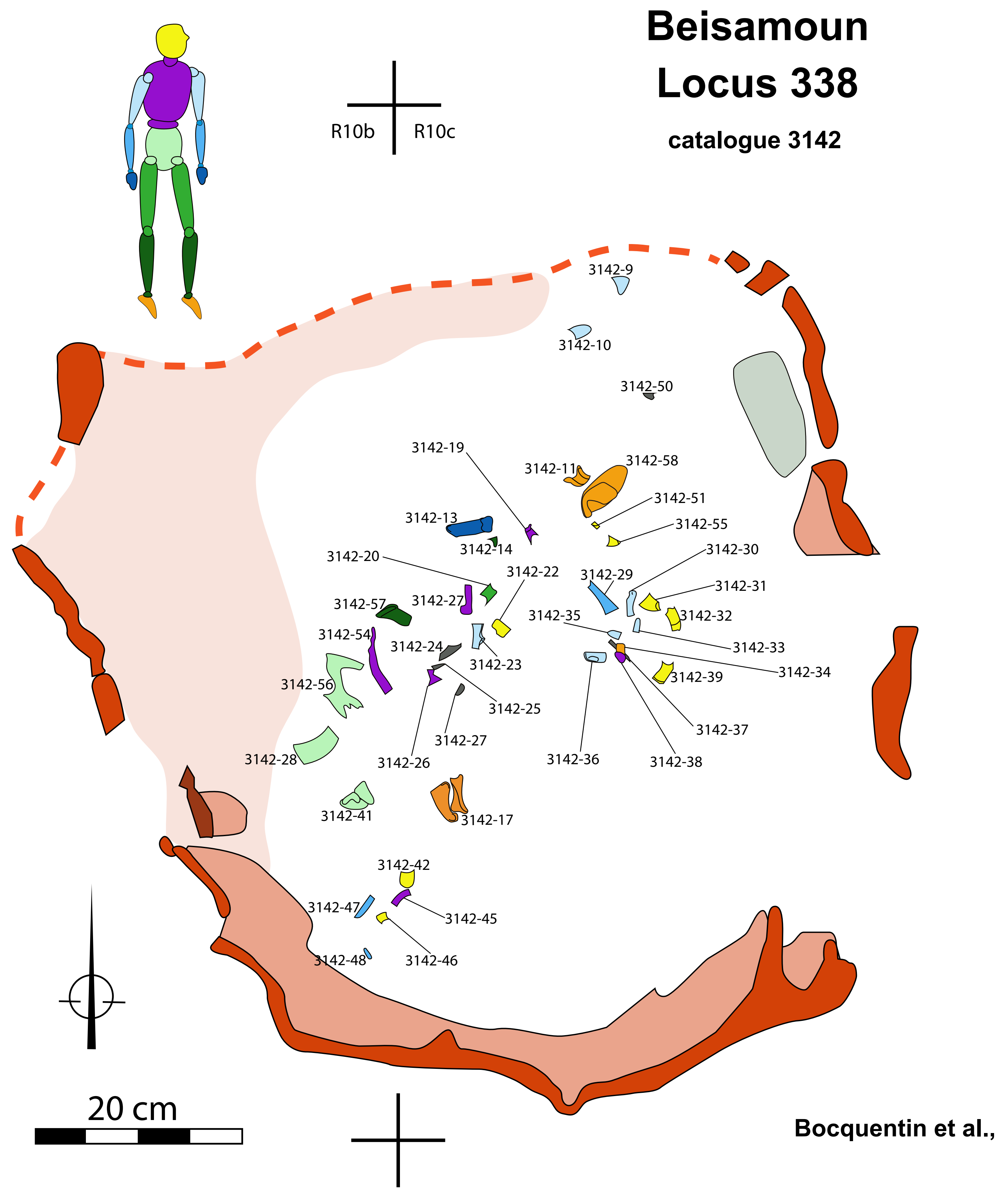
Beisamoun
Locus 338
catalogue 3142
R10b
R10c
3142-9
3142-10
3142-50
3142-19
3142-11
3142-58
3142-51
3142-55
3142-13
3142-14
3142-20
3142-22
3142-29
3142-30
3142-31
3142-57
3142-27
3142-32
3142-35
3142-54
3142-24
3142-23
3142-33
3142-34
3142-39
3142-56
3142-25
3142-37
3142-27
3142-28
3142-26
3142-36
3142-38
3142-41
3142-17
3142-42
3142-47
3142-45
3142-48
3142-46
20 cm
Bocquentin et al.,

Beisamoun
Locus 338
catalogue 3165

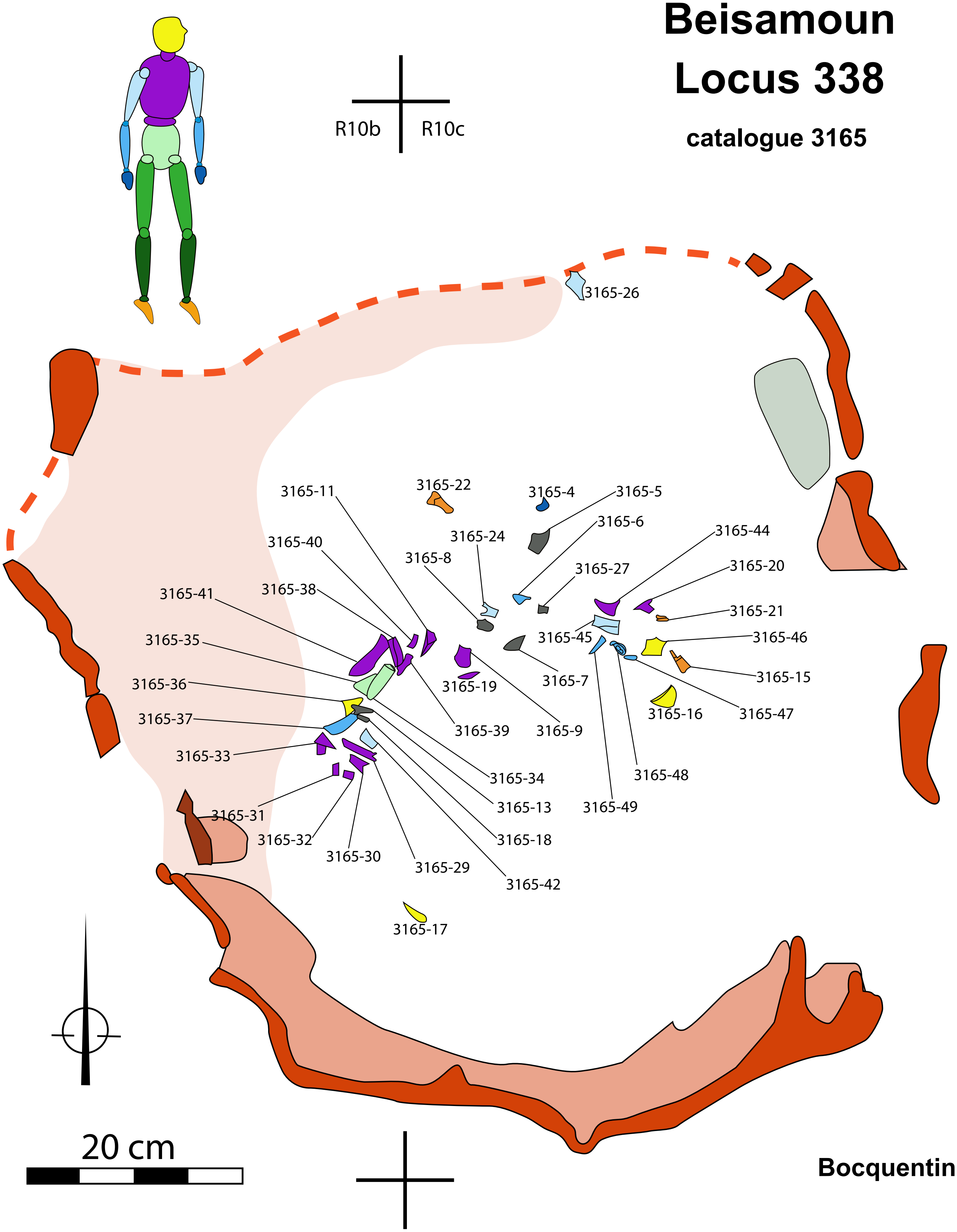

Bocquentin et al.,

Beisamoun Locus 338

catalogue 3176-A

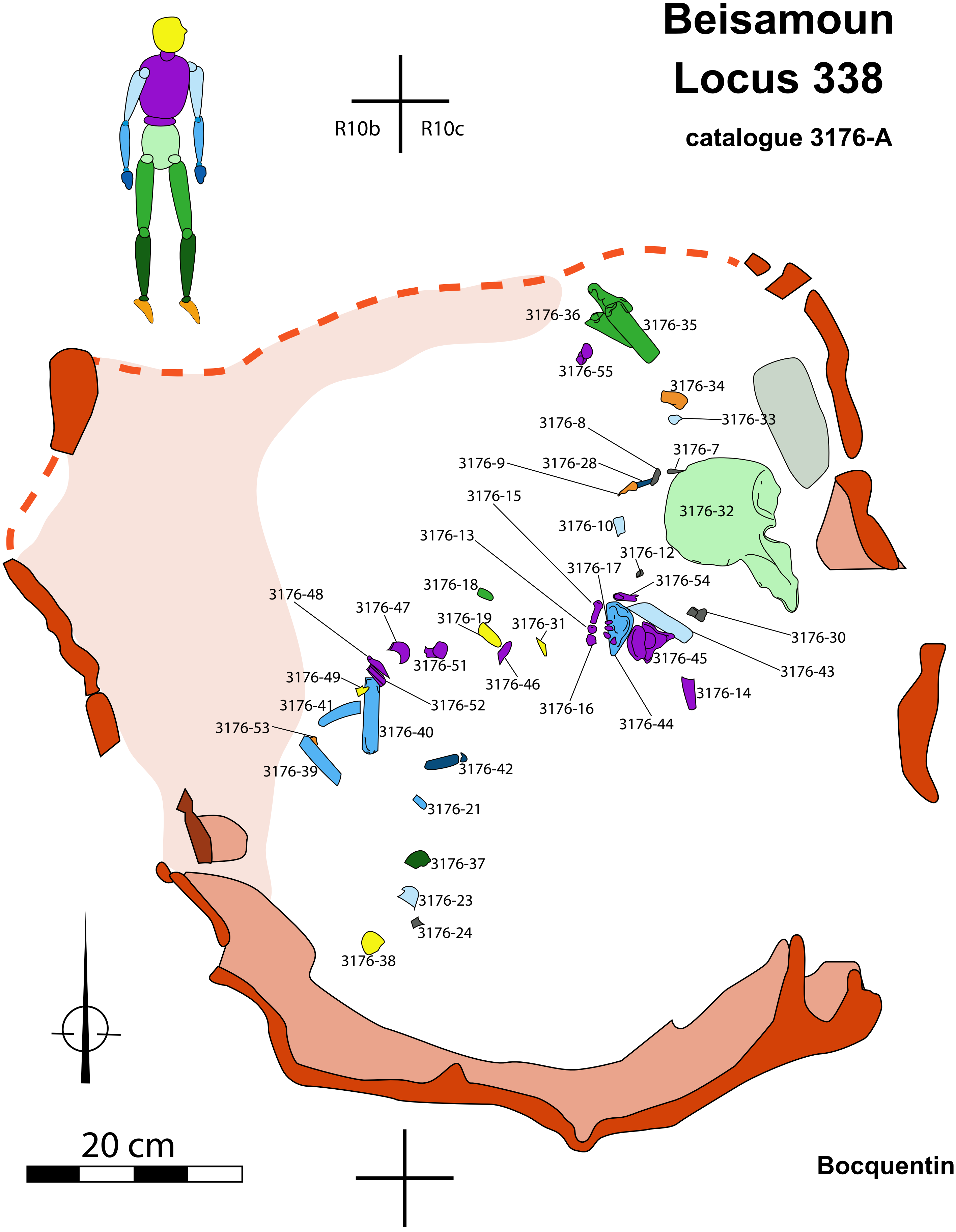

Bocquentin et al.,

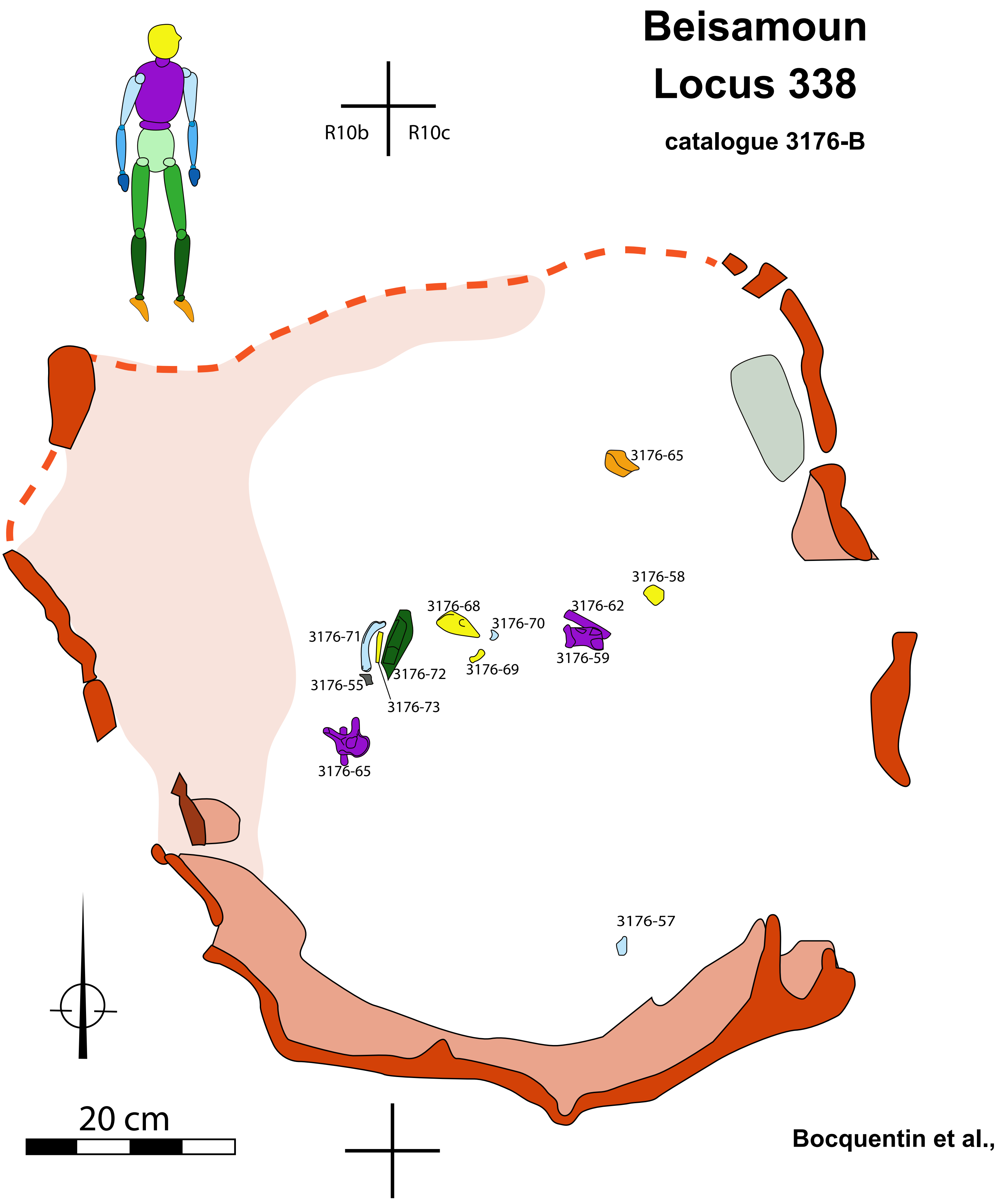
Beisamoun
Locus 338
catalogue 3176-B
R10b
R10c
3176-65
3176-58
3176-68
3176-62
3176-70
3176-71
3176-59
3176-72
3176-69
3176-55
3176-73
3176-65
3176-57
20 cm
Bocquentin et al.,

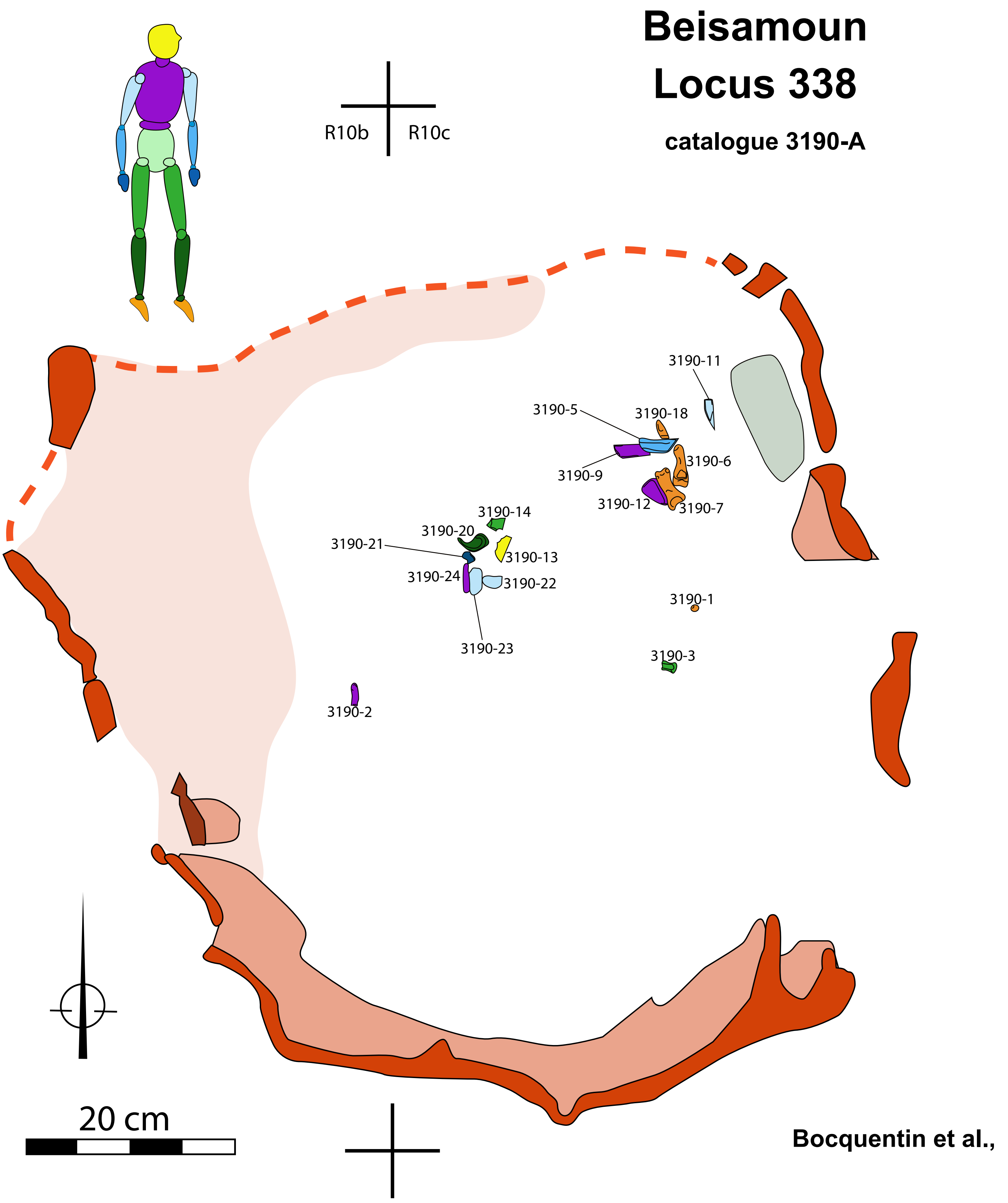
Beisamoun
Locus 338
catalogue 3190-A
R10b
R10c
3190-11
3190-5
3190-18
3190-6
3190-9
3190-12
3190-7
3190-14
3190-20
3190-21
3190-13
3190-24
3190-22
3190-1
3190-23
3190-3
3190-2
20 cm
Bocquentin et al.,

Beisamoun
Locus 338
catalogue 3190-B

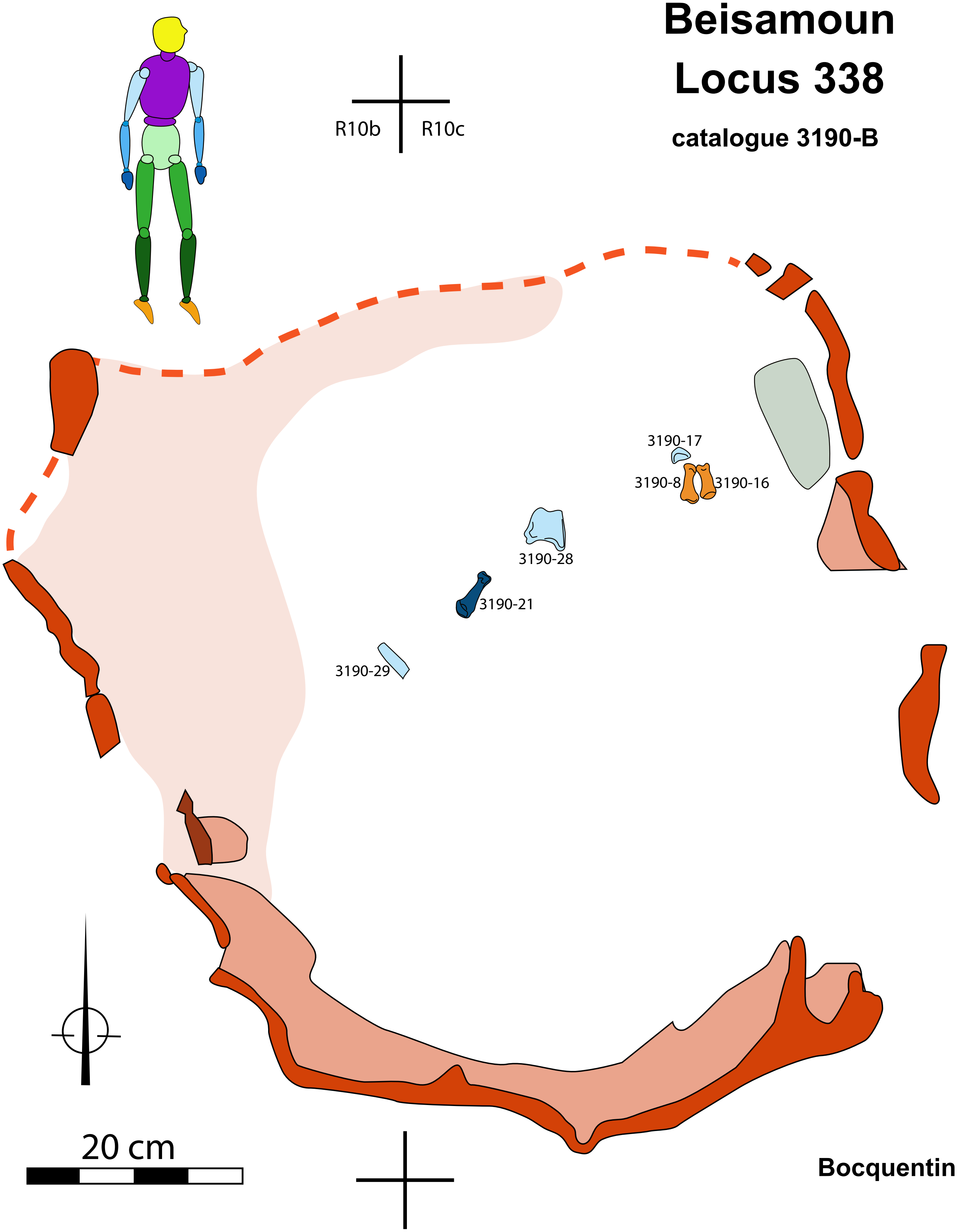

Bocquentin et al.,

Beisamoun
Locus 338
catalogue 3197, 3205

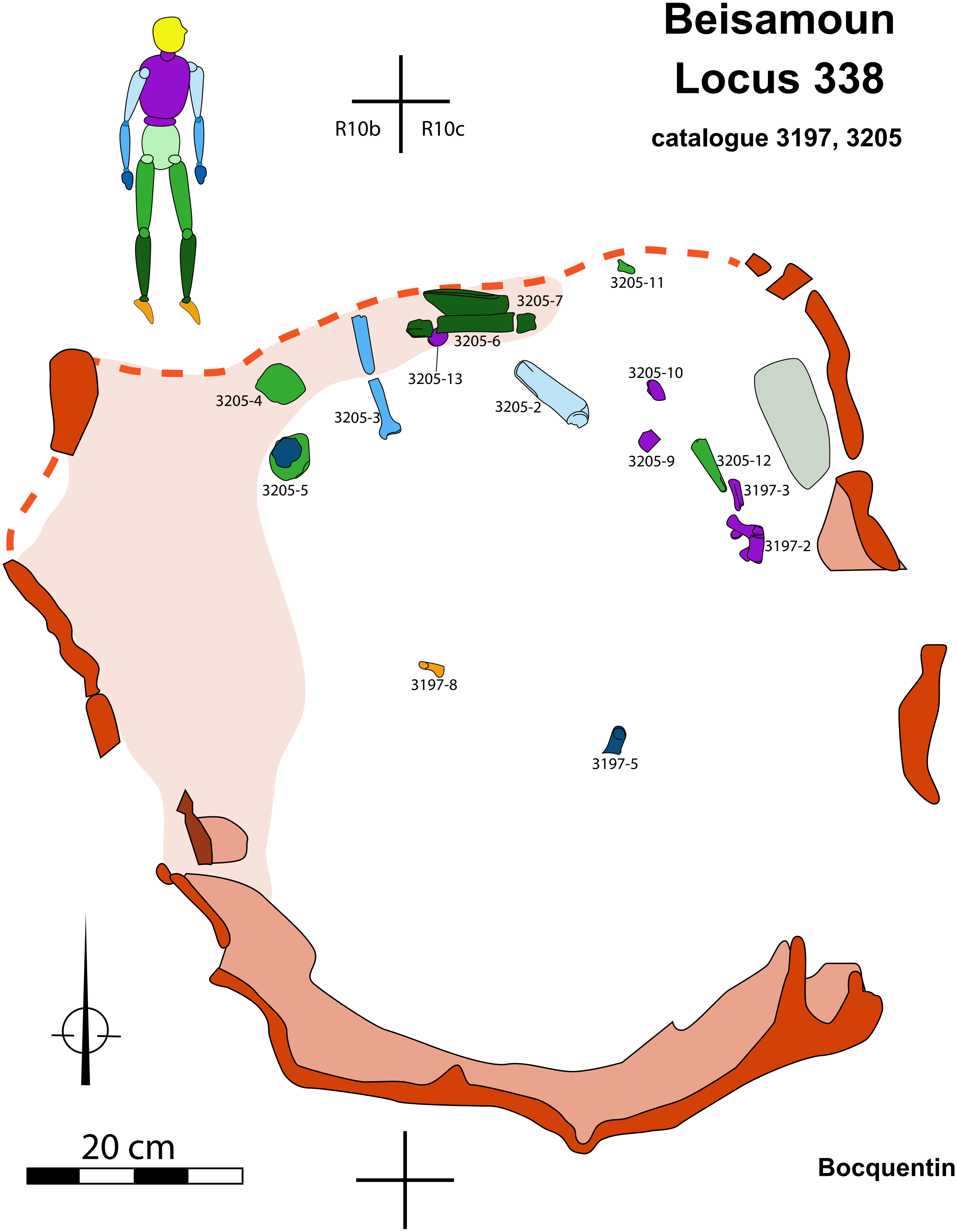

Bocquentin et al.,

Beisamoun
Locus 338

catalogue 3209, 3210

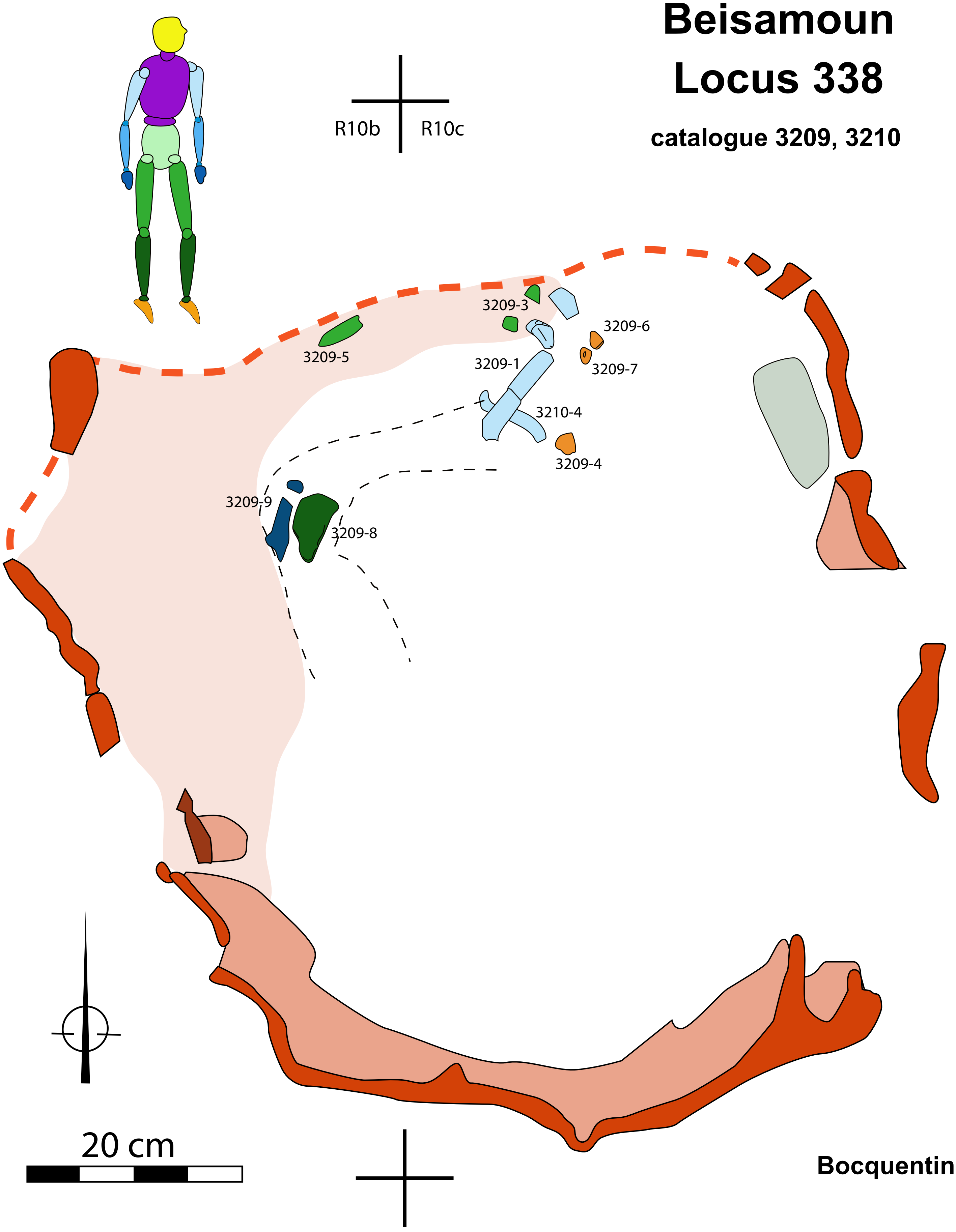

Bocquentin et al.,
