# Supplementary material for: Emergence of corpse cremation during the Pre-Pottery Neolithic of the Southern Levant: A multidisciplinary study of a pyre-pit burial
Source: PLoS One. 2020 Aug 12;15(8):e0235386. doi: 10.1371/journal.pone.0235386 (PMC7423105; doi:10.1371/journal.pone.0235386)
Supplement: S1 Table — https://doi.org/10.7794/13jm-9k62. (PDF) [file pone.0235386.s002.pdf]

Catalogue #	Piece #	Bone description	Anatomical part	Side	Weight (g)	Transverse cracking	Longitudinal cracking	Cracking in U	Other cracks	Shrinking	Distorsion	Fragmentation	External colour	Internal colour	Refits with
2791	2	Second metacarpal	Complete	Right	7,4	No	No	No	No	No	No	No	Not burned	Not burned	
2791	1+4	Proximal pollical phalanx	Complete	Right	5,4	No	No	No	No	No	No	Poor	Not burned	Not burned	
2791	4	Distal pollical phalanx	Complete	Right		No	No	No	No	No	No	No	Not burned	Not burned	
2804	1	Mandible	complete except right mandibular condyle and left coronoid process missing		36,2	Yes	Yes	No	Yes	Yes	Yes	High	White	Gray-white	
2933	Sieving refusal A	Lumbar vertebrae	Left superior articular process		0,9	No	No	No	Yes	No	No	Poor	White	White	
2933	Sieving refusal B	Skull	Several fragments		1,4	No	No	No	No	oui	No	Intermediate	White (out part)	Gray	
2933	1	Occipital bone	fragments with lambdoid suture		12,9	No	Yes	No	Yes	No	No	High	Gray-white with a gray spot	Gray-white with gray-blue spots	2969.2
2933	2	Rib	Head	Right	3,5	No	No	No	No	No	No	Poor	Superior-ventral part : brown with black spots ; dorsal part : brown-gray with white spots	Black	
2933	3	Lower limb	Fragments of shaft	?	8,3	Yes	No	No	Yes	Yes	No	No	White	Gray-white	
2933	4	os coxae	Fragment of illiac arcuate line	Left	4,6	No	No	No	Yes	No	No	Intermediate	Gray-white	Gray	
2933	5	Lumbar vertebrae	Fragments of centrum, inferior and superior articular process		12,3	No	No	No	Yes	No	No	High	Centrum : white ; Lower articular surfaces and apophysis : gray-black ; Upper articular surfaces : gray-white	Centrum : white ; Lower articular surfaces and apophysis : black ; Upper articular surfaces : gray	
2933	6	Rib	Fragment	Left	7,3	No	No	No	No	No	No	Intermediate	anterior part brown-black	brown	
2969	2	Occipital bone	Fragments		5,3	No	No	No	Yes	No	No	High	Endocranial part : white ; Ectocranial part : gray	Gray	2933.1
3015	1	Sacrum				No	Yes	No	No	No	No	No	White	White	
3022	Sieving refusal	Upper limb	Several fragments		9,8	No	No	No	No	No	No	High	Brown to white	Brown to white	
3036	Sieving refusal A	Sternum	Superior fragment of the manubrium		0,5	No	No	No	No	No	No	No	Gray-white	Gray-white	
3036	Sieving refusal B	Bone chip	Spongy fragment		0,2	No	No	No	No	No	No	No	Gray	Black	
3036	Sieving refusal C	Metacarpal	1/2 distal		0,7	No	No	No	Yes	Yes	Yes	Intermediate	Gray-white	Gray-white	
3051	1	Tibia	Fragment of shaft	?	9,8	Yes	No	No	Yes	Yes	No	No	Gray-white	Gray-black	
3051	2	Ulna	1/3 proximal without articular extremity	Right	4,9	Yes	No	No	No	Yes	Yes	Intermediate	White	Gray	
3051	3 (A)	Tibia	Proximal articular extremity	?	4,3	No	No	No	Yes	No	No	No	Gray-blue	Gray	
3051	3 (B)	Thoracic/lumbar vertebrae	Fragment of centrum		1,3	No	No	No	Yes	No	No	No	White	Gray	
3051	3 (C)	Metacarpal or metatarsal	Fragment of shaft	?	1,2	No	No	No	No	Yes	Yes	No	Gray with black spots	Gray	
3051	4	Sacrum	Sacral centrum		17,1	No	Yes	Yes	Yes	Yes	No	High	White	White	
3051	5	Fifth metatarsal	Complete	Left	8,4	No	No	No	No	No	No	No	Black	Black	
3051	6	Patella	Fragments	?	5,8	No	No	No	Yes	Yes	No	High	Brown-gray	Brown-gray	
3051	7	Scapula	Fragment of lateral border	Left?	2,4	No	No	No	Yes	No	No	No	Anterior part : gray ; Posterior part : black	Black	
3051	8	Tibia	Fragment of proximal extremity	Right?	13,1	No	No	No	Yes	Yes	No	High	Gray-white	Gray-white	
3051	10	os coxae	Fragment of ilium, iliac spine	Left	7,4	No	Yes	Yes	Yes	No	No	Intermediate	Gray-white	Gray	
3051	12	Rib	Fragments	?	2,1	No	No	No	No	No	No	High	Brown-black	Black	
3051	13	Rib	Fragment	?	2,1	No	No	No	No	No	No	No	Brown-black	Brown-black	
3051	14	Tibia	Proximal articular extremity	?	4,2	No	No	No	Yes	Yes	Yes	No	Gray-white	Gray-black	
3051	15	os coxae	Fragment of auricular surface	Left	2,5	No	Yes	No	Yes	No	No	High	Brown-grey	Gray	
3051	16	First permanent upper incisor		Right	1,0	No	No	No	No	No	No	No	Root : black ; Crown : grey		
3051	17	Rib	Head and fragments	Right	3,6	No	No	No	No	No	No	High	Head : white ; fragments : black	Head : white ; fragments : black	
3051	18 (A)	Femur	Distal articular extremity	Right	8,8	No	No	No	Yes	Yes	No	Intermediate	Gray-white	Gray-white	
3051	18 (B)	Cervical vertebae	Fragment of centrum		0,9	No	No	No	Yes	Yes	No	No	Grey	Gray	
3051	19	Humerus	1/3 distal (half trochlea and lateral epicondyle)	Right	25,2	Yes	Yes	No	Yes	Yes	Yes	High	Shaft : white ; Trochlea and lateral epicondyle : grey	Shaft : gray ; Distal extremity : white	3190.22
3051	20	Upper limb	Fragment of thin shaft	?	1,9	No	No	No	Yes	Yes	No	High	Gray-white	Gray-white	
3051	21	Upper limb	Fragment of shaft	?	1,1	No	No	No	Yes	Yes	No	No	White	White	
3074	Sieving refusal A	Rib	Bone chips	?	0,4	No	Yes	No	No	Yes	No	Intermediate	White	White	
3074	2	Bone chips			0,6	No	No	No	No	Yes	Yes	Intermediate	Gray-white	Gray	
3102	4	Femur	Fragment of femoral head	?	16,8	No	No	No	No	No	No	No	Gray-blue	Gray	
3102	5	Femur	Fragment of femoral neck	Left	16,4	No	No	No	No	No	No	Poor	Gray-white	Gray-black	3102.7
3102	6	Femur	Fragment of distal extremity : femoral neck	?	4,0	No	Yes	No	Yes	No	No	No	Gray	Black	
3102	7	Femur	Fragment of femoral neck	Left		No	No	No	No	No	No	Poor	Gray-white	Gray-black	3102.5
3114	1	Fibula	Fragment of shaft	Right?	6,5	Yes	Yes	No	Yes	Yes	Yes	Poor	White	White	

3114	2	Long bone	Spongy fragment		0,3	No	No	No	Yes	No	No	No	Gray	Gray	
3114	3	Fibula	Fragment proximal of shaft	Right?	1,5	No	No	No	Yes	Yes	Yes	No	White	White	
3114	4	Vertebrae?	fragment of posterior arch?		0,2	No	No	No	No	Yes	No	No	Gray-white	Gray-white	
3114	5	Thoracic vertebrae	Transverse prcess		0,5	No	No	No	Yes	Yes	No	No	Gray	Gray	
3114	6	Rib or vertebrae	Fragment		0,4	No	No	No	No	No	No	High	Black	Black	
3114	8	Upper limb	Fragment of shaft		0,4	No	No	No	No	No	No	No	White	Black	
3114	9	Humerus	Fragment of shaft	?	1,3	Yes	No	No	No	Yes	No	No	White	Black	
3114	10	Femur	1/4 of shaft	Left	134,1	Yes	No	No	Yes	No	No	High	Anterior part : gray ; Posterior part : white	Anterior part : noir ; Posterior part : gray-black	3176.35, 3176.36
3114	11	Humerus	Fragment of shaft	Left		No	No	No	Yes	No	No	High	Gray-white	Gray-black	3205.2, 3209.1, 3142.10
3114	12	Sesamoid	Complete	?	0,3	No	No	No	No	No	No	No	Black		
3114	13	Talus	Sulcus tali and part of the posterior subtalar facet	Right	3,1	No	No	No	No	No	No	Poor	Black	Black	
3114	14	Upper limb	Fragment of shaft	?	0,4	No	No	No	No	Yes	No	No	White	White	
3114	15	Humerus	Fragment of shaft	?	3,2	Yes	Yes	No	Yes	Yes	Yes	No	Gray-white	Gray-white	
3114	16	Bone chips				No	No	No	No	No	No	High	Brown-gray	Gray	
3114	17	Upper limb	Fragment of shaft	?	1,5	No	No	No	Yes	Yes	No	No	White	Gray-white	
3114	18	Upper limb	Fragment of shaft	?	0,6	No	No	No	No	No	No	No	Gray	Black	
3114	19	Parietal bone	Fragment of inferior temporal line	Right?		No	No	No	Yes	No	No	High	Endocranial part : white ; Ectocranial part : gray-white	Gray	3114.22, 3176.31, 3190.13
3114	20	Temporal bone	Fragment with squamous suture	Right	2,3	No	No	No	Yes	No	No	Poor	Endocranial part : gray ; Ectocranial part : gray-white	Gray	
3114	22	Parietal bone	Fragment with suture	Right?		Yes	Yes	No	No	No	No	High	Endocranial part : white ; Ectocranial part : gray-white	Gray	3114.19, 3176.31, 3190.13
3114	23	Coccyx	Fragment of Cx-1		0,5	No	No	No	Yes	No	No	No	Gray-white	Gray	
3114	24	Rib	Fragment of 1/2	Right	6,6	Yes	Yes	No	Yes	Yes	Yes	High	White	White	
3114	25	Upper limb	Fragment of shaft	?	1,0	No	No	No	No	No	No	No	Gray-black	Gray-black	
3114	26	Rib	Fragment	?	2,3	No	Yes	No	Yes	No	No	Poor	White	White	
3114	27	Lower limb	Fragment of shaft		0,3	No	No	No	No	Yes	No	No	White	Gray	
3114	28	Upper limb	Fragment of shaft		0,5	No	No	No	No	No	No	Intermediate	Black	Black	
3114	29	Triquetral	Complete	Right	1,5	No	No	No	No	No	No	No	Not burned	Not burned	
3114	32	Bone chips			0,0	No	No	No	Yes	Yes	No	Poor	White	Gray	
3114	33	Upper limb	Fragment of shaft	?	2,2	No	No	No	Yes	Yes	No	No	White	Gray	
3114	34	Upper limb	Fragment of shaft	?	0,6	Yes	No	No	No	Yes	No	No	Gray-white	Gray	
3114	35	Tibia	Fragment of shaft	?	5,1	No	Yes	No	Yes	Yes	Yes	No	White	Gray-white	
3114	37	Parietal bone	Fragment	?	2,9	No	No	No	Yes	No	No	Intermediate	Endocranial part : gray-black ; Ectocranial part : white	Gray	
3114	38	Parietal bone	Fragment with sagital suture	Right?	3,6	No	No	No	No	No	No	No	Gray-white with gray spots in lateral part	Lateral part : gray ; Medial part : white	
3114	39	Thoracic vertebrae	Centrum		2,4	No	No	No	Yes	No	No	No	Gray-white	Gray-white	
3114	41	Parietal bone	Fragment	?	0,9	No	No	No	No	No	No	No	Ectocranial part : gray-black	Brown-black	
3114	42	Rib	Fragment		1,6	No	No	No	Yes	Yes	No	No	White	White	
3114	43	Lower limb			0,3	No	Yes	No	Yes	Yes	Yes	No	White	Gray	
3114	44	Lower limb	Fragment of shaft		0,6	No	Yes	No	Yes	Yes	Yes	No	White	Gray	
3114	45	Bone chips	2 fragments		0,3	No	No	No	Yes	Yes	No	Poor	One gray and one white	One gray and one white	
3114	46	Rib	Fragment	?	1,5	No	Yes	No	Yes	Yes	No	No	White	White	
3114	47	Bone chips			0,3	No	No	No	No	Yes	No	Intermediate	White	Gray-white	
3142	Sieving refusal A	Metacarpal	Fragment of shaft		0,5	No	No	No	No	Yes	Yes	No	White	Gray	
3142	Sieving refusal B	Skull	2 fragments : on of eaxh has a suture		0,7	No	No	No	Yes	No	No	Poor	Endocranial part : gray-white ; Ectocranial part : white	One gray and one gray-blue to gray	
3142	Sieving refusal C	Tooth	Root		0,3	No	No	No	No	No	No	High	Gray	Gray-black	
3142	9	Humerus?	Fragment of shaft with spongy bone part	?	2,0	No	No	No	No	No	No	No	Gray	Black	
3142	10	Humerus	Fragment of shaft	Left		Yes	No	No	Yes	No	No	No	Gray	Black	3205.2, 3209.1, 3114.11
3142	11	Proximal hallucial phalanx	1/2 distal	Left	1,0	No	No	No	No	No	No	No	Plantar part : gray-black ; Dorsal part : black	Black	
3142	13	First metacarpal	Complete	Right	5,0	No	No	No	No	No	No	No	Not burned	Not burned	
3142	14	Tibia	1/4 of shaft	Left	19,8	Yes	No	No	Yes	Yes	Yes	Poor	Gray	Gray	3190.20
3142	17	Calcaneus	Talar facets	Left	8,4	Yes	Yes	No	Yes	No	No	High	White	Gray-white	

3142	19	Cervical vertebae	Fragment of corpus		0,9	No	No	No	Yes	No	No	No	Gray	Gray	
3142	20	Lower limb	Fragment of shaft	?	1,7	No	No	No	No	Yes	No	No	Gray-white	Gray-white	
3142	21	Rib	Sternal end	?	0,4	No	No	No	No	Yes	No	No	Gray	Gray	
3142	22	Frontal bone	Fragment		0,6	No	No	No	No	No	No	No	Gray-white	Gray-black	
3142	23	Upper limb	Fragment of shaft	?	1,3	Yes	No	No	No	Yes	No	No	White	White	
3142	24	Bone chips			0,5	No	Yes	No	Yes	Yes	No	No	White	Gray	
3142	25	Bone chips			0,0	No	No	No	No	Yes	No	No	Gray-white	Gray	
3142	26	Thoracic vertebrae	Transverse process		0,0	No	No	No	Yes	Yes	Yes	No	Gray with articular surface more darker	Gray	
3142	27	Bone chips			0,0	No	No	No	Yes	Yes	No	No	Gray-white	Gray	
3142	28 (A)	Sacrum	fragment of S1 with superior part		7,0	No	No	No	Yes	No	No	Intermediate	White	White	
3142	29	Ulna	Fragment of shaft	?	2,3	Yes	No	No	Yes	Yes	No	No	Gray-white	Gray-white	
3142	30	Upper limb	Fragment of shaft		1,6	No	No	No	No	Yes	No	High	White	Gray	
3142	31	Maxillae bone	Fragment		0,8	No	No	No	Yes	Yes	No	No	White	Gray	
3142	32	Parietal bone	Fragment with suture	?	1,6	No	No	No	Yes	No	No	Intermediate	Endocranial part : gray-blue with white spots ; Ectocranial part : white	Gray	
3142	33	Upper limb	Fragment of shaft		0,3	No	No	No	Yes	Yes	No	No	Gray-white	Gray	
3142	34	Metatarsal	1/4 of shaft next to proximal extremity	?	0,5	No	No	No	No	Yes	No	No	White	Gray-white	
3142	35	Upper limb	Fragment of shaft		0,3	No	No	No	Yes	Yes	No	No	Gray-white	Gray	
3142	36	Scapula	Coracoid process	Right	0,9	No	No	No	Yes	No	No	No	Gray	Gray	
3142	37	Bone chips			0,0	No	No	No	Yes	Yes	No	No	White	White	
3142	38	Thoracic vertebrae	Fragment wit costal demifacet		0,0	No	No	No	Yes	Yes	Yes	High	White	White	
3142	39	Parietal bone	Fragment	?	1,2	No	No	No	Yes	No	No	No	Endocranial part : gray-black ; Ectocranial part : gray-white	Gray	
3142	41	os coxae	Fragment with auricular surface	Left	1,9	No	No	No	Yes	Yes	No	High	Gray	Gray	
3142	42	Frontal bone	Fragment		2,2	No	No	No	No	No	No	Poor	Black with gray spots	Black with gray spots	3165.17
3142	45	Rib	Fragment	?	1,2	No	No	No	No	No	No	No	Black	Black	
3142	46	Skull	Fragment with suture		0,3	No	No	No	No	No	No	No	Black with gray spots	Gray	
3142	47	Upper limb	Fragment of shaft	?	1,2	No	No	No	No	No	No	No	Gray	Gray-black	
3142	48	Upper limb	3 fragments of shaft		1,2	No	No	No	No	No	No	Intermediate	Black to white	Black to white	
3142	50	Bone chips			0,6	No	No	No	No	Yes	No	No	White	White	
3142	51	Skull	Fragment		0,4	No	No	No	No	No	No	No	White	Gray-black	
3142	54	Rib	Fragment	?	2,1	No	Yes	No	Yes	Yes	No	Poor	ventral part : white ; dorsal part : white with gray spots	White	
3142	55	Skull	Bone chips		0,3	No	No	No	Yes	Yes	Yes	No	White	Gray-white	
3142	56	os coxae	Fragments of pubis	Left	4,7	No	Yes	No	Yes	Yes	No	High	White	White	
3142	57	Fibula	Fragment of distal extremity	Right	2,7	No	No	No	Yes	No	Yes	Poor	Proximal part : white ; Distal part : gray	Gray	
3142	58	Calcaneus	Posterior talar facet	Right	10,4	No	No	No	Yes	No	No	High	Gray-black	Black	3176.56
3165	2	Bone chips			3,6	No	No	No	No	No	No	High	Black to white	Black to white	
3165	4	Metacarpal	Fragment of distal extremity	?	0,3	No	No	No	Yes	No	No	No	Gray	Gray	
3165	5	Bone chips			0,4	No	No	No	No	No	No	No	Gray	Gray	
3165	6	Radius	Fragment of radial head	Right	0,4	No	No	No	No	No	No	No	Gray	Gray	
3165	7	Bone chips			0,7	No	No	No	No	No	No	No	Gray	Gray	
3165	8	Bone chips			0,3	No	No	No	No	No	No	No	Gray	Gray-black	
3165	9	Vertebrae	Fragment of centrum		0,5	No	No	No	Yes	No	No	No	Gray-white	Gray-black	
3165	11	Lumbar vertebrae	Right inferior articular facet		1,0	No	No	No	Yes	No	No	No	Gray	Gray-black	
3165	13	Bone chips			0,0	No	No	No	Yes	Yes	No	No	White	White	
3165	15	Metatarsal	Fragment of proximal extremity	?	0,9	No	Yes	No	No	Yes	Yes	No	White	Gray	
3165	16	Frontal bone	Fragment		1,1	No	No	No	Yes	No	No	No	Endocranial part : gray-blue ; Ectocranial part : white	Gray	
3165	17	Frontal bone	Fragment			No	No	No	No	No	No	oui	Black with one brown spot	Black with one brown spot	3142.42
3165	18	Bone chips			0,0	No	No	No	No	Yes	No	No	White	Gray	
3165	19	Vertebrae	Spinous process		0,0	No	No	No	Yes	Yes	No	No	Gray-white	Gray-white	
3165	20	Cervical vertebae	Fragment of posterior arch with left superior and inferior facets		0,8	No	No	No	Yes	Yes	No	No	Gray-white	Gray-white	
3165	21	Metatarsal	Fragment of shaft	?	0,4	No	No	No	Yes	Yes	Yes	No	White	Gray-white	
3165	22	Proximal foot phalange	Without proximal articular surface	Left	0,9	No	No	No	No	No	No	No	Black	Black	
3165	23	Bone chips			0,2	No	No	No	No	Yes	No	No	Gray	Gray	
3165	24	Upper limb	Fragment of shaft	?	0,8	No	No	No	Yes	No	No	No	Gray-white	Gray-white	

3165	26	Upper limb	Fragment of shaft		0,6	No	No	No	Yes	No	No	No	Gray-white	Gray-white	
3165	27	Bone chips			0,0	No	No	No	Yes	Yes	No	No	Gray-white	Gray	
3165	29 (A)	Rib	Fragment	?	0,9	No	Yes	No	Yes	Yes	No	Poor	White	White	
3165	29 (B)	Cervical vertebae	Fragment of centrum		0,4	No	No	No	No	No	No	No	Gray	Gray	
3165	30	Vertebrae	Spinous process		0,4	No	No	No	Yes	No	No	No	White	White	
3165	31	Vertebrae	Fragment of inferior articular facet		0,0	No	No	No	Yes	No	No	No	Gray-white	Gray-white	
3165	32	Thoracic vertebrae	Transverse proces		0,3	No	No	No	Yes	No	No	No	White	White	
3165	33	Thoracic vertebrae	Left transverse process		1,5	No	No	No	Yes	No	No	No	Posterior part : white ; Anterior part : gray	Gray	
3165	34	os coxae	Ischiopubic ramus	Left	1,3	No	Yes	No	Yes	Yes	Yes	No	Gray-white	Gray	
3165	35	os coxae	Bone chips	Left	0,3	No	No	No	Yes	Yes	Yes	Intermediate	White	Gray	
3165	36	Temporal bone	Fragment with squamous suture	?	1,0	No	Yes	No	No	Yes	Yes	No	White	Gray	
3165	37	Ulna	Fragment of shaft	Right	2,9	No	No	No	Yes	Yes	Yes	Poor	White	Gray	
3165	38	Rib	Fragment	?	0,5	No	Yes	No	No	No	No	Poor	Gray	Gray	
3165	39	Rib	Fragment	?	0,2	No	No	No	Yes	No	No	High	White	White	
3165	40	Rib	Fragment	?	0,5	No	No	No	Yes	No	No	Intermediate	White	White	
3165	41	Rib	Head	Right	2,0	No	Yes	No	Yes	Yes	Yes	No	White	White	
3165	42	Upper limb	Fragment of shaft	?	2,4	Yes	Yes	No	Yes	Yes	Yes	No	White	White	
3165	43	Upper limb	Fragment of shaft	?	2,0	Yes	No	No	Yes	Yes	Yes	No	White	White	
3165	44	Thoracic vertebrae	Rigth transverse process and part of posterior arch		1,5	No	Yes	No	Yes	No	No	No	Posterior part : gray-black with gray spots ; Anterior-inferior part : gray ; Anterior-superiod part : white	Posterior part : gray-black ; Anterior : white	
3165	45	Upper limb	Fragment of shaft	?	2,8	Yes	No	No	Yes	Yes	Yes	No	Gray-white	Gray-white	
3165	46	Parietal bone	Fragment with sagital suture		0,9	No	No	No	No	Yes	No	No	Gray-white	Gray-white	
3165	47	Ulna	Fragment of distal extremity	Right	0,2	No	No	No	No	Yes	No	No	Gray-white	Gray	
3165	48	Ulna	Fragment of coronoid process and radial notch	Right	0,5	No	No	No	Yes	Yes	No	No	Gray	Gray	
3165	49	Ulna	Fragment of shaft next to proximal extremity with radial notch	Right	0,7	No	No	No	No	Yes	Yes	No	White	White	
3176	1	Bone chips			2,3	No	No	No	No	No	No	High	Brown to white	Brown to white	
3176	7	Bone chips			0,0	No	No	No	No	Yes	No	No	Gray	Gray-black	
3176	8	Bone chips			0,3	No	No	No	No	No	No	High	Gray	Black	
3176	9	Metatarsal	Fragment of shaft next to distal extremity	?	0,4	No	No	No	No	No	No	No	Black	Black	
3176	10	Upper limb	Fragment of shaft	?	0,6	Yes	No	No	Yes	Yes	No	No	White	Gray-white	
3176	13	Vertebrae	Fragment		0,2	No	No	No	No	No	No	Poor	Gray-black	Gray	
3176	14	Rib	Fragment	?	0,9	No	Yes	No	No	No	No	No	White	White	
3176	15	Rib	Head	Right	0,4	No	No	No	Yes	No	No	No	Gray	Gray	
3176	16	Thoracic vertebrae	Fragment of centrum with superior costal demifacet		0,4	No	No	No	Yes	No	No	No	Gray	Gray	
3176	17	Lumbar vertebrae	Fragment of left superior articular facet		1,4	No	No	No	Yes	No	No	High	Gray-white	Gray-white	
3176	18	Lower limb	Fragment of shaft		0,9	No	No	No	No	No	No	No	Brown	Brown	
3176	19	Frontal bone	Fragment of temporal line		1,2	No	No	No	Yes	No	No	No	Endocranial part : gray-black ; ectocranial part : gray-white	Gray-black	
3176	21	Radius or ulna	Fragment of shaft		0,6	No	No	No	No	No	No	No	Brown-black	Brown-black	
3176	23	Upper limb	Fragment of shaft	?	0,8	No	No	No	No	No	No	No	Gray-black	Gray-black	
3176	24	Bone chips			0,2	No	No	No	No	No	No	No	Brown-black	Brown-black	
3176	28	Distal hand phalange	Complete	Left?	0,1	No	No	No	Yes	No	No	No	Proximal part : gray ; Distal part : white		
3176	28	Metacarpal	Shaft	?	1,1	No	No	No	No	No	No	High	Gray	Black	
3176	29	Bone chips			0,3	No	No	No	Yes	Yes	Yes	No	Gray	Gray	
3176	30	Bone chips			1,0	No	No	No	No	No	No	Intermediate	Brown to gray	Brown to gray	
3176	31	Parietal bone	Fragment	Right?	8,8	No	No	No	Yes	No	No	Intermediate	Endocranial part : gray-white ; Ectocranial : white	Gray	3114.19, 3114.22, 3190.13
3176	32	os coxae	Without pubis	Right	107,9	No	No	No	Yes	Yes	No	High	Gray-white	Gray-white	
3176	33	Humerus	Fragment of shaft	?	2,5	Yes	No	No	Yes	Yes	No	No	White	White	
3176	34 (A)	Navicular	Fragment of facet for the talar head	Right	2,6	No	No	No	No	No	No	No	Posterior part : gray-black ; Anterior part : black	Black	
3176	34 (B)	Bone chips			0,4	No	No	No	No	Yes	No	Intermediate	Gray	Gray	

3176	35	Femur	1/5 of shaft	Left		Yes	No	No	Yes	No	No	High	Posterior part : gray-white ; Anterior part : gray	Black	3114.10, 3176.36
3176	36	Femur	Fragment of shaft	Left		No	No	No	Yes	No	No	High	Gray-white	Black	3176.35, 3114.10
3176	37	Fibula	Proximal extremity	?	2,7	No	No	No	Yes	No	No	No	White	White	
3176	38	Occipital bone	Fragment of right part to the foramen magnum with a part of atlas articular facet		2,6	No	No	No	No	No	No	Poor	Gray	Gray	
3176	39	Ulna	1/4 of shaft next to distal extremity	Left	10,1	No	No	No	No	No	No	No	Black	Black	
3176	40	Radius or ulna	Fragment of shaft	?	2,0	Yes	No	No	No	Yes	Yes	High	White	White	
3176	41	Radius	Fragment of shaft	?	2,1	Yes	Yes	No	Yes	Yes	Yes	Poor	White	Gray-white	
3176	42	Metacarpal	Shaft	?	1,3	No	No	No	No	No	No	Intermediate	Gray-black	Black	
3176	43	Humerus	Fragment of shaft	Right	8,3	Yes	Yes	No	No	Yes	No	Poor	White	White	
3176	44	Ulna	Fragment of oleocranon	Right	2,7	No	No	No	Yes	Yes	No	Intermediate	Gray-white	Gray-white	
3176	45	Thoracic vertebrae	Posterior arch and a part of centrum without transverse process		4,4	No	Yes	No	Yes	No	No	No	Posterior part : gray with gray-black spots ; Anterior part : gray	Posterior part : gray ; Anterior part : white	
3176	46	Rib	Fragment	?	0,3	No	No	No	No	No	No	No	White	White	
3176	47	Cervical vertebae	Centrum		1,5	No	No	No	Yes	No	No	No	Gray-white	Gray	
3176	48	Rib	Fragment	?	0,5	No	No	No	Yes	Yes	Yes	No	White	White	
3176	49 (A)	Thoracic vertebrae	Fragment of centrum		0,6	No	No	No	No	No	No	No	White	White	
3176	49 (B)	Skull	fragment		0,5	No	No	No	Yes	Yes	No	No	White	White	
3176	51	Lumbar vertebrae	Fragment of posterior arch		2,2	No	No	No	Yes	No	No	Poor	Gray	Gray	
3176	53	Proximal foot phalange	Fragment of proximal articular surface	?	0,0	No	No	No	No	No	No	No	Black	noir	
3176	54	Thoracic vertebrae	Articular facet of the posterior arch		0,6	No	No	No	Yes	No	No	No	Posterior part : white with a gray spot ; Anterior part : gray-white	White	
3176	55 (A)	Cervical vertebae	Articular facet of the posterior arch		0,7	No	No	No	Yes	No	No	No	Gray	Gray	
3176	55 (B)	Bone chips			0,4	No	No	No	No	No	No	No	Gray-white	Gray-white	
3176	56	Calcaneus	Anterior talar facet	Right	2,9	No	No	No	No	No	No	No	Gray-black	Black	3142.58
3176	57	Upper limb	Articular facet of the posterior arch		0,6	No	No	No	No	No	No	No	Gray	Gray	
3176	58	Maxillae bone	Fragment		1,1	No	No	No	No	No	No	Poor	Gray	Gray	
3176	59	Thoracic vertebrae	Fragment of centrum		2,3	No	No	No	No	No	No	High	Gray	Gray	
3176	62	Rib	Head	Right	1,6	No	Yes	No	Yes	No	No	No	dorsalpart : gray ; ventral part : gray-white	Gray	
3176	64 (A)	Thoracic vertebrae	Posterior arch with part of centrum		5,9	Yes	Yes	No	Yes	No	No	Poor	White	White	
3176	64 (B)	Rib	Sternal end	?	0,2	Yes	No	No	Yes	Yes	No	No	White	White	
3176	65	Rib	Fragment	?	0,7	No	Yes	No	No	Yes	No	No	Lateral part : white ; ventral part : gray	Gray	
3176	66	Bone chips			0,5	No	No	No	Yes	Yes	No	High	Black to gray	Gray	
3176	68	Petrous bone	External acoustic meatus	Left	6,9	No	No	No	Yes	Yes	Yes	Poor	Gray-white	Gray	
3176	69	Frontal bone	Fragment of glabella		0,9	No	No	No	No	No	No	No	Endocranial part : gray ; ectocranial part : white	Gray	
3176	70	Upper limb	Fragment of shaft	?	0,9	No	No	No	Yes	Yes	No	No	Gray	Gray-black	
3176	71	Clavicule	1/3 lateral	Right	5,4	No	Yes	No	Yes	Yes	Yes	Intermediate	Gray-white	Gray	
3176	72	Tibia	Fragment of shaft	Right	11,0	No	No	No	Yes	Yes	Yes	Intermediate	White	White	
3176	73	Frontal bone	Fragment of right orbitae with frontozygomatic suture and supraorbital foramen		2,1	No	No	No	No	No	No	Poor	Gray	Gray	
3176	74	Lumbar vertebrae	Spinous process		0,6	No	No	No	Yes	No	No	No	Gray	Gray	
3176	75	Upper limb	Fragment of shaft	?	0,8	No	No	No	No	Yes	No	No	White	White	
3190	Sieving refusal A	Metatarsal	Fragment of shaft		0,5	No	No	No	No	No	No	Poor	Gray-black	Black	
3190	1	Intermediate foot phalange	Complete		0,0	No	No	No	No	No	No	No	Black	Black	
3190	2	Vertebrae	Spinous process		0,0	No	No	No	Yes	Yes	No	Intermediate	Gray-white	White	
3190	3	Lower limb	Fragment of shaft	?	2,1	Yes	No	No	Yes	Yes	No	No	White	White	
3190	5	Ulna	Ulnar tuberosity	Left	2,5	No	No	No	No	No	No	No	Gray-black	Black	
3190	6	Proximal foot phalange	Complete	Right	1,7	No	No	No	No	No	No	No	Black		
3190	7	Proximal foot phalange	Complete	Right	1,3	No	No	No	Yes	No	No	No	Lateral part : black ; Medial part : black with a gray spot		

3190	8	Proximal foot phalange	Complete	Right	0,9	No	No	No	No	No	No	No	Proximal part : black ; Distal part : black with a grey spot		
3190	9	Rib	Fragment	?	0,9	No	No	No	No	No	No	Poor	Lateral part : black ; ventral part : brown-black	Black	
3190	10	Metatarsal	Fragment of shaft	?	0,7	No	No	No	No	No	No	No	Gray	Black	
3190	11	Upper limb	Fragment of shaft		0,7	No	No	No	No	No	No	No	Gray	Gray	
3190	12	Rib	Fragment	?	0,6	No	No	No	No	No	No	No	Gray	Black	
3190	13	Parietal bone	Fragment	Right?		No	No	No	No	No	No	High	Endocranial part : gray-white ; Ectocranial part : white	Gray	3114.19, 3114.22, 3176.31
3190	14	Lower limb	Fragment of shaft		0,8	No	No	No	No	No	No	No	Gray-white	Gray-black	
3190	16	Proximal foot phalange	Complete	Right	1,0	No	No	No	No	No	No	No	Black		
3190	17	Upper limb	Fragment of shaft		0,3	No	No	No	No	No	No	No	Gray	Black	
3190	20	Tibia	1/4 of shaft	Left		Yes	No	No	Yes	Yes	Yes	Poor	Gray	Gray	3142.14
3190	21	Proximal hand phalange	Complete	Left?	3,5	No	No	No	No	No	No	No	Black with distal extremity gray-black		
3190	22	Humerus	Fragment of trochlea	Right		No	No	No	No	No	No	No	White	White	3051.19
3190	23	Humerus	Fragment of shaft	?	1,2	No	No	No	No	No	No	No	Gray-white	Gray	
3190	24	Rib	Fragment	?	0,0	No	No	No	No	Yes	No	No	White	White	
3190	28	Scapula	Fragment of acromion	Left	10,1	No	No	No	No	No	No	No	Gray-white	Black	
3190	29	Upper limb	Fragment of shaft	?	1,8	No	No	No	No	Yes	No	No	White	White	
3197	Sieving refusal A	Rib	Fragment	?	1,2	No	Yes	No	Yes	No	No	Poor	Gray-white	Gray	
3197	Sieving refusal B	Rib	Fragments	?	0,0	No	No	No	No	No	No	No	Gray-black	Gray-black	
3197	2	Thoracic vertebrae	Centrum with part of posterior arch		6,6	No	No	No	Yes	No	No	Poor	Gray	Gray	
3197	3	Rib	Fragment	?	1,3	No	No	No	Yes	Yes	No	No	Gray	Gray	
3197	5	Capitate	Complete	Right	1,3	No	No	No	No	No	No	No	Black	Black	
3197	8	Distal hallucial phalanx	Complete	Left	1,0	No	No	No	No	No	No	No	Plantar part : gray-black ; Dorsal part : black with a brown spot	Black	
3203	Sieving refusal A	Rib	Head	?	0,9	No	Yes	No	No	No	No	No	ventral part : gray-white ; dorsal part : dark gray	Gray	
3203	Sieving refusal B	Upper limb	Fragment of shaft		3,4	No	No	No	No	No	No	High	Gray-white	Gray-blue	
3203	Sieving refusal C	Thoracic vertebrae	Fragment of transverse process		0,2	No	No	No	Yes	No	No	No	Gray with white spots	Gray	
3203	Sieving refusal D	Frontal bone	Fragment		1,5	No	No	No	No	No	No	No	Endocranial part : gray-white ; Ectocranial part : white	Gray to gray-black	
3205	Sieving refusal A	Lumbar vertebrae	Superior articular facet		1,1	No	No	No	Yes	No	No	No	Gray-black	Black	
3205	Sieving refusal B	Sesamoid	Complete		0,3	No	No	No	No	No	No	No	Black	Black	
3205	Sieving refusal C	Lumbar vertebrae	Left superior articular facet		1,1	No	No	No	Yes	Yes	No	No	Gray-white	Light gray	
3205	2	Humerus	Fragment of shaft	Left		Yes	No	No	No	No	No	High	Gray	Black	3209.1, 3114.11, 3142.10
3205	3	Radius	1/3 proximal	Left	15,0	Yes	Yes	No	Yes	No	No	Intermediate	Proximal-anterior part : brown ; Proximal-posterior part : black ; Distal part : gray with interosseous border in white color	Gray-black	
3205	4	Femur	Fragment distal extremity		5,2	No	No	No	No	No	No	Poor	Gray-black	Brown-black	
3205	5 (A)	Femur	Fragment distal extremity	Left	34,3	No	No	No	No	No	No	High	Lateral part : brown ; Medial part : black	Lateral part : brown ; Medial part : black	3205.11
3205	5 (B)	Trapezium	Complete	Left		No	No	No	No	No	No	High	Black	Black	
3205	6 et 7	Tibia	1/4 of shaft next to proximal extremity	Right	44,2	Yes	Yes	No	Yes	No	No	High	Gray	Black	
3205	9	Rib	Fragment	?	1,9	No	No	No	No	No	No	No	Black	Black	
3205	10	Rib	Fragment	?	1,9	No	No	No	No	No	No	No	Black	Black	
3205	11	Femur	Fragment distal extremity	Left		No	No	No	No	No	No	High	Black	Black	3205.5
3205	12	Femur or tibia	Fragment of shaft	?	9,4	No	No	No	No	No	No	No	Gray	Gray	
3205	13	Rib	Head	Left	0,5	No	No	No	No	No	No	Poor	Gray-black	Black	
3209	Sieving refusal A	Lateral cuneiform	Complete	Left	1,1	No	No	No	Yes	No	No	No	Gray-white	Gray-white	
3209	Sieving refusal B	Thoracic vertebrae	Transverse process		0,5	No	No	No	Yes	No	Yes	No	Gray-white	Gray-white	
3209	Sieving refusal C	Scaphoid?	2 fragments		0,8	No	No	No	Yes	Yes	No	Poor	White	Gray	
3209	Sieving refusal D	Rib	Head and fragments	?	0,6	No	No	No	No	No	No	Poor	Gray with gray-black spots	Head : black ; Fragments : gray	

3209	Sieving refusal E	Talus	Fragment of superior articular facet, medial malleolar surface and lateral malleolar surface	Left	0,7	No	No	No	No	No	No	No	Gray-black	Gray	
3209	1	Humerus	1/3 distal without articular surface	Left	62,2	Yes	Yes	No	Yes	No	No	High	Gray	Black	3205.2, 3142.10, 3114.11
3209	2	Scapula	Fragment of acromion	Left	2,6	No	No	No	No	No	No	High	Gray-white	Black	
3209	3	Lower limb	Spongy fragment	?	1,6	No	No	No	No	Yes	No	No	Gray-white	Gray	
3209	4	First metatarsal	Distal extremity	Right	2,5	No	No	No	No	No	No	No	Anterior-superior part : gray-black ; Posterior part : black	Black	
3209	5	Femur or tibia	Fragment of shaft	?	3,2	No	No	No	No	No	No	No	Gray-black	Brown-black	
3209	6	Metatarsal	Fragment of proximal extremity	?	0,6	No	No	No	No	No	No	High	Black	Black	
3209	7	Proximal hallucial phalanx	Proximal articular surface	Left	1,5	No	No	No	No	No	No	High	Black	Black	
3209	8	Tibia	Fragment of proximal extremity	?	12,9	No	No	No	Yes	No	No	No	Gray-white	Gray-black	
3209	9 (A)	Lunate	Complete	Left	0,6	No	No	No	No	No	No	No	Black	Black	
3209	9 (B)	Third metacarpal	1/3 proximal	Left	1,3	No	Yes	No	No	No	No	No	Gray-white	Gray-black	
3209	9 (C)	Metacarpal	1/2 distal	?	2,0	No	No	No	No	No	No	No	Black	Black	
3209	9 (D)	Trapezium	Complete	Left	0,5	No	No	No	No	No	No	No	Black	Black	
3209	9 (E)	Thoracic vertebrae	Centrum with transverse process, as well as left superior articular facet and right inferior articular facet		6,0	No	Yes	No	Yes	No	No	High	Gray	Gray	
3209	9 (F)	Rib	3 fragments	?	5,0	No	Yes	No	No	No	No	Intermediate	Gray-white	Gray-white	
3209	9 (G)	Lower limb	Fragments of shafts		6,9	No	No	No	No	No	No	No	Gray-black to white	Gray-black to white	
3209	9 (H)	Parietal bone	3 fragments	Left	4,2	No	No	No	Yes	No	No	Intermediate	Endocranial part : gray-black ; Ectocranial part : gray-white	Gray-black	
3209	9 (I)	Calcaneus	Fragment of facet for the cuboid	Right	1,9	No	No	No	No	No	No	No	Gray-black	Gray-black	
3209	9 (J)	First metacarpal	Fragment of distal articular surface	Left	0,3	No	No	No	No	No	No	No	Brown-black	Brown	
3210	Sieving refusal A	Rib	Fragments of arch	Left	4,2	No	No	No	No	No	No	No	Gray-black with sternal extremity gray	Black	
3210	Sieving refusal B	Parietal bone	Fragments		7,5	No	No	No	Yes	No	No	High	Endocranial part : gray-black ; Ectocranial part : gray-white	Gray	
3210	Sieving refusal C	Rib	Fragments	?	0,3	No	No	No	Yes	Yes	No	Poor	White	White	
3210	Sieving refusal D	Lower limb	Fragments of shaft		3,8	No	No	No	Yes	No	No	Poor	White	Black	
3210	Sieving refusal E	Rib	Fragment of arch	?	0,6	No	Yes	No	Yes	Yes	Yes	No	Gray-white	Gray	
3210	Sieving refusal F	Thoracic vertebrae	Fragment of transverse process		0,4	No	No	No	Yes	No	No	No	Gray-white	Gray	
3210	Sieving refusal G	Rib	Fragment of tubercle	?	0,3	No	No	No	Yes	Yes	Yes	No	Gray	Gray	
3210	1	Clavicule	2/3 lateral	Left	13,0	No	No	No	No	No	No	Intermediate	Lateral aprt : black with white spots ; Medial part : gray-black with white spots	Black	
3210	2 (A)	Thoracic vertebrae	Posterior arch with part of centrum		6,3	Yes	Yes	No	Yes	No	No	Poor	White	White	
3210	2 (B)	Hamate	Complete	Left	1,2	No	No	No	Yes	No	No	No	Gray-white	White	
3210	2 (C)	Ribs	Fragments of arch	?	5,4	Yes	Yes	No	No	No	No	High	White	White	
3210	2 (D)	Zygomatic bone	Almost complete	Left	0,1	Yes	Yes	Yes	Yes	Yes	Yes	No	White	White	
3210	2 (D)	Zygomatic bone	Almost complete	Right	0,1	Yes	Yes	Yes	Yes	Yes	Yes	No	White	White	
3210	2 (D)	Maxillae bone	Fragment of frontal process	Right	0,1	Yes	Yes	Yes	Yes	Yes	Yes	No	White	White	
3210	2 (D)	Tibia	Fragment of distal articular surface	?	0,1	No	No	No	Yes	Yes	Yes	No	White	White	
