# Supplementary material for: Emergence of corpse cremation during the Pre-Pottery Neolithic of the Southern Levant: A multidisciplinary study of a pyre-pit burial
Source: PLoS One. 2020 Aug 12;15(8):e0235386. doi: 10.1371/journal.pone.0235386 (PMC7423105; doi:10.1371/journal.pone.0235386)

PLOS ONE

Emergence of corpse cremation during the Southern Levantine Pre Pottery Neolithic

--Manuscript Draft--

**Manuscript Number:**	PONE-D-19-25674
**Article Type:**	Research Article
**Full Title:**	Emergence of corpse cremation<br>during the Southern Levantine Pre Pottery Neolithic
**Short Title:**	Emergence of cremation practice in the Near East
**Corresponding Author:**	Fanny Bocquentin<br>Centre National de la Recherche Scientifique<br>Nanterre, FRANCE
**Keywords:**	Corpse; cremation; Pre Pottery Neolithic; PPNC; 8th and 7th millennium cal BC; C14; Near East; Levant; Pyre; Taphonomy; Archaeothanatology; bioanthropology; zooarchaeology; soil micromorphological; phytolith; Projectile Point; injury; fuels; Beisamoun; funeral operational sequence; emergence; spatial analysis; human remains
**Abstract:**	Renewed excavations at the Neolithic site of Beisamoun (Upper Jordan Valley, Israel) has resulted in the discovery of a Late 8 th and 7 th millennium occupation layer (Final PPNB or PPNC: ca 7200-6400 cal BC). Several human graves have been unearthed within the habitation area, temporarily abandoned. They testify to a great diversity of funerary forms: primary burials, secondary deposits, skull removal, cranial plastering and cremations. One of the cremations represents a young adult individual who was cremated, in situ, in a pyre that also served as the final burial place. The right fibula has been directly dated to 7031-6700 cal BC making it the earliest corpse cremation yet found in the Near East. The deceased previously survived from a flint projectile injury, still embedded to the left shoulder. Archaeothanatology, bioanthropology, zooarchaeology, soil micromorphological analysis, and phytolith identification are integrated in order to explore the different stages and techniques involved in the ritual sequence: cremation pit construction, selection of fuel, primary position of the corpse, potential associated items and funerary containers, fire management, post-cremation gesture and structure abandonment. The origins and development of cremation practices in the region are explored as well as their significance in terms of Northern-Southern Levantine connections during the transition between the 8 th and 7 th millennium.
**Order of Authors:**	Fanny Bocquentin
	Marie Anton
	Francesco Berna
	Arlen Rosen
	Hamoudi Khalaily
	Harris Greenberg
	Thomas C. Hart
	Omri Lernau
	Liora K. Horwitz
**Additional Information:**	
**Question**	**Response**
**Financial Disclosure**<br><br>Enter a financial disclosure statement that	The Irene Levi Sala Care Archaeological Foundation supported financially this research project untitled Domesticating decay during the Pre-Pottery Neolithic: emergence of corpse cremation in Southern Levant (F. Bocquentin and A. Rosen Dir.).

Powered by Editorial Manager® and ProduXion Manager® from Aries Systems Corporation

describes the sources of funding for the work included in this submission. Review the submission guidelines for detailed requirements. View published research articles from *PLOS ONE* for specific examples.<br><br>This statement is required for submission and **will appear in the published article** if the submission is accepted. Please make sure it is accurate.<br><br>**Unfunded studies**<br>Enter: *The author(s) received no specific funding for this work.*<br><br>**Funded studies**<br>Enter a statement with the following details:<br>• Initials of the authors who received each award<br>• Grant numbers awarded to each author<br>• The full name of each funder<br>• URL of each funder website<br>• Did the sponsors or funders play any role in the study design, data collection and analysis, decision to publish, or preparation of the manuscript?<br>• **NO** - Include this sentence at the end of your statement: *The funders had no role in study design, data collection and analysis, decision to publish, or preparation of the manuscript.*<br>• **YES** - Specify the role(s) played.<br><br>* typeset	We are also indebted to our host institutes and financial supporters which make possible the renewed excavation program at Beisamoun: French National Center of Scientific Research (CNRS, UMR7041, Ethnologie Préhistorique), Israel Antiquities Authority (IAA), French center of Research at Jerusalem (CRFJ), French Ministry of Foreign Affairs (MEAE)
**Competing Interests**<br><br>Use the instructions below to enter a competing interest statement for this submission. On behalf of all authors, disclose any competing interests that could be perceived to bias this work—acknowledging all financial support and any other relevant financial or non-financial competing interests.<br><br>This statement **will appear in the published article** if the submission is accepted. Please make sure it is accurate. View published research articles from *PLOS ONE* for specific examples.	The authors have declared that no competing interests exist

Powered by Editorial Manager® and ProduXion Manager® from Aries Systems Corporation

**NO authors have competing interests**<br><br>Enter: *The authors have declared that no competing interests exist.*<br><br>**Authors with competing interests**<br><br>Enter competing interest details beginning with this statement:<br><br>*I have read the journal's policy and the authors of this manuscript have the following competing interests: [insert competing interests here]*<br><br>* typeset	
**Ethics Statement**<br><br>Enter an ethics statement for this submission. This statement is required if the study involved:<br><br>• Human participants<br>• Human specimens or tissue<br>• Vertebrate animals or cephalopods<br>• Vertebrate embryos or tissues<br>• Field research<br><br>Write "N/A" if the submission does not require an ethics statement.<br><br>General guidance is provided below. Consult the submission guidelines for detailed instructions. **Make sure that all information entered here is included in the Methods section of the manuscript.**	Field Permit Number : G39-2012; G41-2013<br>Israel Antiquities Authority

Powered by Editorial Manager® and ProduXion Manager® from Aries Systems Corporation

**Format for specific study types**<br><br>**Human Subject Research (involving human participants and/or tissue)**<br>• Give the name of the institutional review board or ethics committee that approved the study<br>• Include the approval number and/or a statement indicating approval of this research<br>• Indicate the form of consent obtained (written/oral) or the reason that consent was not obtained (e.g. the data were analyzed anonymously)<br><br>**Animal Research (involving vertebrate animals, embryos or tissues)**<br>• Provide the name of the Institutional Animal Care and Use Committee (IACUC) or other relevant ethics board that reviewed the study protocol, and indicate whether they approved this research or granted a formal waiver of ethical approval<br>• Include an approval number if one was obtained<br>• If the study involved *non-human primates*, add *additional details* about animal welfare and steps taken to ameliorate suffering<br>• If anesthesia, euthanasia, or any kind of animal sacrifice is part of the study, include briefly which substances and/or methods were applied<br><br>**Field Research**<br><br>Include the following details if this study involves the collection of plant, animal, or other materials from a natural setting:<br>• Field permit number<br>• Name of the institution or relevant body that granted permission	
**Data Availability**<br><br>Authors are required to make all data underlying the findings described fully available, without restriction, and from the time of publication. PLOS allows rare exceptions to address legal and ethical concerns. See the PLOS Data Policy and FAQ for detailed information.	Yes - all data are fully available without restriction

Powered by Editorial Manager® and ProduXion Manager® from Aries Systems Corporation

A Data Availability Statement describing where the data can be found is required at submission. Your answers to this question constitute the Data Availability Statement and **will be published in the article**, if accepted.<br><br>**Important:** Stating 'data available on request from the author' is not sufficient. If your data are only available upon request, select 'No' for the first question and explain your exceptional situation in the text box.<br><br>Do the authors confirm that all data underlying the findings described in their manuscript are fully available without restriction?	
**Describe where the data may be found in full sentences. If you are copying our sample text, replace any instances of XXX with the appropriate details.**<br><br>• If the data are **held or will be held in a public repository**, include URLs, accession numbers or DOIs. If this information will only be available after acceptance, indicate this by ticking the box below. For example: *All XXX files are available from the XXX database (accession number(s) XXX, XXX.).*<br>• If the data are all contained **within the manuscript and/or Supporting Information files**, enter the following: *All relevant data are within the manuscript and its Supporting Information files.*<br>• If neither of these applies but you are able to provide **details of access elsewhere**, with or without limitations, please do so. For example:<br><br>*Data cannot be shared publicly because of [XXX]. Data are available from the XXX Institutional Data Access / Ethics Committee (contact via XXX) for researchers who meet the criteria for access to confidential data.*<br><br>*The data underlying the results presented in the study are available from (include the name of the third party*	I do not understand

and contact information or URL).<br>• This text is appropriate if the data are owned by a third party and authors do not have permission to share the data.<br><br>* typeset	
Additional data availability information:	

Manuscript
Click here to download Manuscript Bocquentin-et-al-Emergence-cremation-corpse-NE.docx

Emergence of corpse cremation during the Southern Levantine Pre Pottery Neolithic

Fanny Bocquentin[1*], Marie Anton[2,3], Francesco Berna[4], Arlene Rosen[5] Hamoudi Khalaily[6,] Harris Greenberg[7], Thomas C. Hart[5], Omri Lernau[8], Liora Kolska Horwitz[9]

[1] UMR 7041 du CNRS. ArScAn, Equipe Ethnologie Préhistorique. MSH Mondes, Nanterre, France.

[2] Université Paris 1, Panthéon-Sorbonne, Paris, France.

[3] UMR 7206 du CNRS. Musée de l'Homme, Éco-Anthropologie et Ethnologie, Paris, France.

[4] Department of Archaeology, Simon Fraser University, Burnaby, Canada.

[5] Department of Anthropology, University of Texas, Austin, United States of America

[6] Israel Antiquities Authority, Jerusalem, Israel.

[7] Department of Archaeology, Boston University, Boston, United States of America.

[8] Zinman Institute of Archaeology, University of Haifa, Haifa, Israel.

[9] National Natural History Collections, The Hebrew University, Jerusalem, Israel.

***** Corresponding author
fanny.bocquentin@cnrs.fr

Abstract

Renewed excavations at the Neolithic site of Beisamoun (Upper Jordan Valley, Israel) has resulted in the discovery of a Late 8th and 7th millennium occupation layer (Final PPNB or PPNC: ca 7200-6400 cal BC). Several human graves have been unearthed within the habitation area, temporarily abandoned. They testify to a great diversity of funerary forms: primary burials, secondary deposits, skull removal, cranial plastering and cremations. One of the cremations represents a young adult individual who was cremated, in situ, in a pyre that also served as the final burial place. The right fibula has been directly dated to 7031-6700 cal BC making it the earliest corpse cremation yet found in the Near East. The deceased previously survived from a flint projectile injury, still embedded to the left shoulder. Archaeothanatology, bioanthropology, zooarchaeology, soil micromorphological analysis, and phytolith identification are integrated in order to explore the different stages and techniques involved in the ritual sequence: cremation pit construction, selection of fuel, primary position of the corpse, potential associated items and funerary containers, fire management, post-cremation gesture and structure abandonment. The origins and development of cremation practices in the region are explored as well as their significance in terms of Northern-Southern Levantine connections during the transition between the 8th and 7th millennium.

Introduction

The treatment of the dead during the Neolithization process in the Near East is a complex process that reflects part of the cognitive and symbolic world that guided this economic and dietary

shift [e.g. articles in 1]. Burial location and funerary gestures are diversified from one community to another as well as from one individual to another. Several operational sequences in burial practice co-exist resulting in primary, secondary, plural, single, staging, manipulations and/or removals that were carried out, sometimes side by side. Occasional defleshing, and/or dismemberments, and temporary mummification are suspected to have been practiced as well. The variety of treatments has its roots in the preceding Natufian culture, and possibly even before, but becomes more complex and diversified throughout the Pre-Pottery Neolithic period [amongst recent data e.g.: 2-10].

The practice of cremation has been mentioned several times for pre-Ceramic periods but often questioned, since accidental fire exposure could not be completely ruled out. From the beginning of sedentarization [e.g.: 11-14], or later periods [e.g. 8, 15, 16], hearths are regularly situated close to, or with, graves. In addition, the presence of burnt isolated human bones is not exceptional but remains poorly understood due to the lack of accurate contextual and taphonomic studies [synthesis in: 15]. One exception are the burnt human bones recovered in the Early Natufian occupation at Kebara Cave [17]. The anthropological analysis demonstrated secondary deposition of burnt bones belonging to a minimum of 31 individuals. Of these, only one could have been burnt as a fresh corpse, the others having been burnt most probably after the decay process. The colouration of the bones favour burning at a modest temperature, in a fire not conducted: an isolated Epipaleolithic case quite difficult to interpret [13: 151-160].

To definitively identify the practice of cremation, it is necessary to be able to demonstrate the intentionality of burning a corpse (primary cremation) or its bone remains (secondary cremation). This intentionality can be determined by the presence of three lines of evidence: the application of appropriate techniques for the intention, the recognition of pyre structures, and the repetition of unusual or suspicious cases. The practice of cremation has multiple reasons, that may differ from one society to another, and the symbolism it conveys is just as varied and can be positive (privilege) or negative (sacrilege) [e.g. : 18-21; for the post-Neolithic Near East see e.g. : 22]. As far as primary cremations are concerned, however, the expected result is common: it is a question of accelerating and taking control of the defleshing of the corpse by replacing the natural decomposition of the flesh with an anthropic action. It is a question of taming, in a way, of domesticating, an unavoidable biological process via a complex technical protocol. Indeed, the incineration of a corpse requires combustion at high temperature (>600°), for several hours or days. The greater the combustion is managed (constant heat, segments of corpses recentered in the furnace) the faster and more complete the process will be [e.g. 20]. For cremations of already dried remains (see *infra* note 4), the intentionality and expected result is more vague as combustion will change the bones little except their colour and, at high temperature, will crack, distort and fragment them. Thus, the technical constraints, specific to the intention, are in this case likely to be varied. Amongst cases of burnt bones, the Skull Building in Çayönü, dated to the end of the Pre-Pottery Neolithic period, is one of the most spectacular [15]. However, this combustion is not considered as a collective cremation (i.e. as its primary intention) but as a condemnation of the funeral building itself by fire. The time of funerary treatment is over when fire is used. To the contrary, Grave 556 at Tell Aswad (Syria) attests to the intentional firing of dried bones in a Middle PPNB context (8200-7500 cal BC). The remains of a young child appear to have been selectively removed from a primary burial, redeposited and burnt in situ as the pit show clear traces of burning [8: 212-213]. The bones, that are beige to black in colour, appear to have been exposed to a moderate temperature.

Indisputable evidence for cremation practices (associated with a pyre, part of a recurrent practice) is known in the Near East from the site of Tell Aïn el Kherk in a Pottery Neolithic context, dated to the middle of the 7th millennium BC [23].

An example of on an earlier instance of cremation is presented in this paper. The cremation was found at the site of Beisamoun, northern Israel. It enables us trace this practice back to the end of the 8th millennium BC, at the time of the critical transition period between the Late Pre-Pottery Neolithic B (PPNB) and the Pre-Pottery Neolithic C (PPNC or Final PPNB).

1. The chrono-cultural context

The end of the 8th millennium marks a break in the sphere of interactions that linked the Northern and Southern Levant through cultural exchange and probable population movements throughout the PPNB [e.g. 24]. In the Northern Levant, there is a continuation in site occupation and the adoption of pottery (Dark-Faced Burnished Ware). In the Southern Levant, many of the imposing villages occupied during the Late PPNB (megasites) were abandoned and pottery was adopted only some 600 to 800 years later. This so-called "Palestinian hiatus", long considered as a gap lacking a unique identity, is now a better known thanks to recent excavations at key sites whose occupation continues throughout the PPNC (also known as the Final PPNB) such as ‘Aïn Ghazal, Wadi Shueib, Beisamoun and most recently Motza.

Material culture at this time does not show a break in the way things were done or materials produced (architecture, tools, vessels etc.), but rather a standardization and a lower degree of skill accompanied by a diversification of production. The period is characterised by an agro-pastoral economy although the hunting of targeted species might be maintained in the most game-rich areas (e.g. wild boar at Beisamoun [25]). It is now likely~~,~~ that many PPNC occupations were not identified in excavation, and were considered as mixed levels between the PPNB and the subsequent early Pottery Neolithic (EPN) (e.g. at Munhata). However, a significant demographic decline cannot be ruled out given the profound change in the pattern of settlement occupation [26-29]. Among the skeletons discovered and studied to date, a dramatic increase in physiological stress is evident that hinders bone and dental maturation as well as an increase in the evidence of infectious diseases [synthesis in 30, 31]. Around 6400-6200 cal BC, pottery production is attested to in the Southern Levant, essentially marking the end of the Pre-Pottery cultures.

2. Beisamoun

Beisamoun is a Pre-Pottery Neolithic settlement located nearby the ancient Hula Lake (Fig 1) that was occupied during the 8th and 7th millennia and estimated at more than 10 ha in extent, [25, 32-34]. The renewed excavations from 2007 to 2016 under the direction of F. Bocquentin and H. Khalaily, reveal the very end of the occupation before abandonment during which a strong stratigraphic, architectural and symbolic continuity with the Late PPNB is observed, including in the funeral treatment of the deceased. There is a great diversity of funerary practices on the site, with a total of 28 single or double graves, including 17 primary burials, 9 secondary (4 are of cremated remains) and a pyre-grave (Locus 338) which is the subject of this article. There are a minimum of 33 individuals including 18 adults and 15 immatures (12 are infants).

Fig 1. Map of sites mentioned in the text (Ground Map: M. Sauvage, CNRS).

3. The pyre-grave 338: building, functioning, abandonment

3.1 The structure

Locus 338 is located in Sector E of the site (Squares R10 and R11). It is a U-shaped pit that reaches 80 cm in its largest diameter and is 60 cm deep, dug down from Layer Ib into Layer Ic. Layer Ib corresponds to a collapse of mud brick belonging to the earlier phase (Layer Ic) before reconstruction of the next phase (Layer I). The area seems to have been temporarily dedicated to burial and ritual activities as witnessed by ceremonial arrangements (plateforms, ritual animal deposit yet unpublished). Graves are preferably dug inside ~~previous~~ houses near the partially ruined walls.

The walls of the pit, indurated and reddish (two cm of thickness), were easily identified during the excavation but brittle once exposed. The eastern and southern edges had a wide opening before sinking vertically; the western edge had a bench about 20 cm wide. The northern edge of the pit was not preserved, but the distribution of the bones and their steep dip make it possible to reconstruct the outline of this wall with sufficient certainty (dashed line on the drawing Fig 2). The appearance of the wall is irregular both in shape and thickness. Soil micromorphological analysis revealed that the wall of the pit was plastered with a cm-thick carbonatic mud-based material resembling the raw material used to for some of the mud bricks excavated at the site. Vesicular micro-porosity and iron depletion pedofeatures indicate that this plastering material had been applied wet to the walls of the pit (Fig 3) and eventually fired in situ.

Fig 2. Structure 338. A: Map of the cremation pit; B: Identification of the upper edges of the pit and ongoing excavation of its south-east fourth. C: Details of a piece of mud-plastered wall (ref 3142-12; copyright L. Teira). D. Detail of the north-east fourth during excavation process. A fragment of collapsed wall inside the pit is visible (star).

Fig 3. Micromorphology of the exogenous material use to plaster the cremation pit. A) Photograph of structure 338 and the sampling location (red box) of the plastered wall. B) Macroscan of the 50x75mm petrographic thin section processed from the intact block sampled from the area indicated by the red box in figure 3A. Note the groundmass with spongy peds of reddish-yellow (7.5YR 6/6) to very pale brown (10YR 7/4) clay with common silt to very fine sand sized grains of quartz and calcite. cc = heated limestone. Hcl = heated terra rossa soil aggregates. Sh = gastropod shell. C) Microphotograph of the contact between the plastered wall surface of structure 338 and the sediment matrix filling structure 338. D) Microphotograph of area (red block in photo C) of well- preserved carbonatic mud plaster from the wall of structure 338. Note the massive light gray (10YR 7/1) matrix of calcium carbonate, with frequent (30-50% of field of view) silt-sized (2-20 µm) grains of quartz and calcite. Mixed into the calcium carbonate matrix are small fragments of weathered bone, shell, phytoliths, basalt fragments, and charcoal. Portions of the structure are slightly decalcified. The exterior edge appears to be physically degraded, so any burnishing or surface decoration is not preserved.

3.2 The deceased

355 fragments of identified human bone have been unearthed from this cremation pit. They belong to all parts of the skeleton although they differ in frequency i.e. teeth, carpals and tarsals are scarce. No duplication of skeletal elements exists and the Minimal Number of Individuals was estimated as one (Fig 4). Indeed, it is most likely that all pieces belong to the same individual: a young

adult. The age is based on the fact that all preserved epiphysis are fused, but vertebrae S2 and S3 are only partially fused (fusion before 30 years old see [35]). Sex could not be estimated with enough confidence as the bones are highly fragmented and mostly calcinated. The vertebrae have remodelled joint surfaces compatible with moderate arthritis.

Fig 4. ~~Conservation~~ sheet of the cremated individual. Coloration of bones are specified.

The total weight of this bone assemblage is 1158.7 g. This reinforces the presence of a single individual because the individual reference weight averages are higher (for a female and *a fortiori* for a male) [e.g.: 36, 37]. The removal of a few bones at the end of cremation is not excluded, particularly among the least well represented skeletal elements (see conservation sheet), but difficulties of identification due to fragmentation ~~is real~~.

This young deceased had been the victim of interpersonal violence which it survived for at least a few weeks. A fragment of a projectile point (of 11.6 mm length preserved) was still embedded in the base of its left scapular spine (piece of bone measure 32.82 mm of length). The perforated bone has remodelled around the flint fragment, which had been broken by bending at both ends (Fig 5). The orientation of the projectile shows that it penetrated through the posterior surface of the bone from the top left towards the infraspinous fossa. It perforated the entire height of the scapular spine. Depending on the position of the victim and the movement of the arm at the time of impact, the shooter could be positioned either to the back or on the left side of the deceased. The infraspinatus muscle was possibly torn, which most likely caused a large hematoma and severe pain but not necessarily impaired function. Today usually, 6 weeks to a few months might be necessary for the cortical bone to remodel. Here the cortical tissue is totally healed and ~~do not~~ show specific signs of infection. The projectile tip remained ~~into~~ the shoulder until the individual's death (a few months or few years after the injury). Because of the cremation treatment of its corpse, the flint shows several thermal pot lid fractures and a change of colour. The broken tip (0.5 -1.5 mm) is not present in the remodelled bone and must have fall into the pyre.

Fig 5. Projectile point embedded in the left scapula (ref 3209-2). A: Model seen from its back showing the location and direction of entrance of the projectile point within the bone (red triangle). B: Posterior anatomical view of the piece of scapular spine preserved. C. Infero-posterior anatomical view of the piece of scapular spine preserved. Note the healed bone around the projectile tip. D. Detail of the projectile tip (copyright A. Legrand). E. Photogrammetric acquisition of the inferior anatomical view (copyright R. Brageu). F. Detail of the flexion break of the proximal segment of the preserved projectile point (copyright R. Brageu). G and H: 3D acquisition: the projectile point embedded in the bone is seen completely; the ~~full reconstruction~~ of the cortical bone is confirmed (copyright F. Edon and D. Beloeil).

3.3 Spatial analysis of the human remains

The bones are distributed throughout the bottom of the structure, partly superimposed one on the other to a thickness of 40 cm. However, the density of remains was not very marked except at the centre of the pyre (Fig 6). If an apparent anatomical anarchy is visible at first, some interesting observations can be made while looking at the details. Skull fragments are found only in the southern half of the structure. Next to the south wall at an upper level, we found the base of the skull (mandible reversed and occipital fragments); the rest of the cranial vault and face (frontal, maxillars, parietals and temporals) were found at the centre of the pit. ~~To the contrary,~~ the cervical vertebrae were distributed out from the centre to the northern half of the pyre. The thoracic column and some of the ribs ~~are~~ concentrated in the centre following roughly a west-east direction. The lumbar vertebrae were found in the middle and against the south-western wall of the structure with several vertebral

fragments in close proximity to the sacrum, coccyx, and the left coxal. The right coxal is found diametrically opposite to this coherent group, lying almost complete not far from the North-Eastern wall of the pit. Altogether, despite an absence of articulated joints, and some long distance of dispersion, the axial skeleton shows some anatomical coherence. Neither is the appendicular skeleton lying in total disorder: both sides are not totally comingled but distributed preferentially to the north-western area for the left side and south-eastern area for the right side (Fig 7). The left shoulder, arm and bones of the forearm (scapula, clavicle, humerus and radius) were found clustered to the north. The bones of the right hand are grouped together at the south-east part of the structure, and the proximal and distal phalanx of the thumb were found articulated. Last but not least, four proximal phalanges of the right foot were found next to each other in anatomical position, distal part oriented towards the North, under the right coxal.

Fig 6. Spatial distribution of bones within Locus 338 according to anatomical categories. The injured scapula fragment is circled in black.
Fig 7. Spatial distribution of right and left sides of the appendicular skeleton.

Thus, the inventory of bones and their relative position favour the deposit of an articulated corpse and not dislocated bones (primary cremation). The non-random distribution of the appendicular skeleton, the trunk and skull bones suggests that the body was oriented along a south/north axis, probably in a seated position leaning against the south, south-east or south-west wall of the structure. This is also compatible with the narrowness of the pit. If we consider that the four feet phalanges are likely close to their initial position, then it would suggest that the corpse was placed with knees flexed, but not tightly. The cremation would have resulted in the trunk falling forward and its possible rotation.

3.4 The combustion process

3.4.1 Temperature of combustion

3.4.1.1 Colorimetric data:

Visual examination shows that all shades of discolouration are present on the bones of Locus 338 (Figs 8 and 9). To the southeast some bones of the right hand and a rib do not have signs of heating. They probably tipped over to the periphery of the hearth quite quickly. The dominant colour is grey-white, that is to say bones close to or at the stage of calcination. Transverse and longitudinal cracks, as well as deep cracks, are numerous. Several bones are distorted and shrunk, signatures of cremation at high temperatures and compatible with the hypothesis of a primary cremation sustained previously (although the distinction between fresh and dry bones from colour or cracks remain much debated [e.g. 38-42]). The skull, the vertebral column as a whole, and the left coxal are the most intensely burned elements, followed by the upper limbs and then the lower limbs. It can be considered that the position of the hearth but also the structure of the bones are factors that have influenced this colour gradation.

Fig 8. **Picture of bones in situ:** A. Segment of axial skeleton: ribs and vertebrae exposed in the middle of the structure. B. Right coxal in situ preserved almost complete by of a piece of collapsed mud wall (see Fig 2). C. 4 right feet proximal phalanges found directly under the right coxal.

Fig 9. Spatial distribution of human bones according to colorimetric data.

4.4.1.2 FTIR data:

The results of the FTIR analysis of the bones are summarized in Table 1[1]. FTIR revealed that in the analyzed tooth, two human skull fragments and five other unidentified bone fragments the original biogenic carbonate hydroxyapatite (CHA) is partially transformed into hydroxyapatite (HA), indicating that these fragments were heated above 500°C. The human skull fragments in particular show extremely reduced FTIR absorptions of carbonate as expected for bones that have been calcined at temperatures of 700-800°C (Fig 10). Several bone fragments that showed the typical black and brown discoloration of charring show no FTIR absorptions of HA. This mineralogical characteristic indicates that these bone fragments were exposed to temperatures below 500°C. In fact charring of bone collagen occurs at temperatures of between ca. 300 and 400°C [44].

	Sample ID #	Description	Discoloring	FTIR Analysis*	Integrated interpretation
1	338 3102-9	Humerus prox	Grey	CHA+C+K+Q	heated below 500°C + soil crust
2	3 383 114	Bone frag	Black	CHA+HA*tr*	heated to ca 500°C
3	3 383 114	Bone frag	Grey	CHA+C+K+Q	heated below 500°C + soil crust
4	338 3176-43	Human skull frag	Calcined	HA+CHA	heated above 500°C below 900°C
5	3 383 210	Human skull frag	Calcined	HA+CHA	heated above 500°C below 900°C
6	3 383 190	Bone frag larger	Grey-white	HA+CHA	heated above 500°C below 900°C
7	3 383 190	Bone frag smaller	Grey-white	HA+CHA+Q+C+K	heated above 500°C below 900°C + soil crust
8	3 383 190	Bone frag	Brown	CHA+C+Q	unheated or heated below 500°C + soil crust
9	3 383 197	Bone frag	Calcined	HA+CHA	heated above 500°C below 900°C
10	3 383 197	Tooth (enamel)	Black	CHA+HA*tr*	heated above 500°C
10a	3 383 197	Tooth (dentin)	Black	CHA	charred below 500°C
11	3 383 197	Bone frag	Brown	CHA	unheated or heated below 500°C
12	3 383 205	Bone frag	Unburnt	CHA	unheated
13	3 383 209	Bone frag	Black	CHA+C	charred below 500°C + calcite crust
14	3 383 209	Bone frag	Brown	CHA	unheated or heated below 500°C
15	3 383 210	Bone frag	Black	CHA+1617*s*	charred collagen = heated below 500°C
16	3 383 210	Bone frag	Unburnt	CHA+C+Q	unheated + soil crust
17	338 3142-53	Tibia shaft frag	Black	CHA	charred and heated below 500°C
18	338 3142-8	Bone frag	Unburnt	CHA+C	unheated + calcite crust
19	338 3176-50	Mandible condyle -subsurface	Brown	CHA	unheated or heated below 500°C
19a	338 3176-50	Mandible condyle -surface	Brown	CHA	unheated or heated below 500°C
20	338 3176-77	Bone frag	Unburnt	CHA+HA*tr* +3570	heated above 500°C below 900°C
21	338 3190-18	*Bos* rib proximal	Unburnt	CHA	unheated
22	3 383 190	Pig maxilla	Unburnt	CHA+C	unheated + calcite crust
23	338 3176-20	Cemented mud-brick material	Grey	Q+C+Cl*a* +582	Mud-brick material heated above 700°C

*C = calcite; Cl= clay minerals; CHA = carbonate-hydroxyapatite; HA = hydroxyapatite; Q = quartz; K = kaolinite; *a* = altered; *s* =strong; *tr* = trace;

Table 1. FTIR analysis of a selection of bone, teeth and mud brick fragments excavated from Locus 338 at Beisamoun.

Fig 10. **FTIR spectra of representative bone samples from Beisamoun, Locus 338**. (A) Fragment of cow rib apparently unburnt to a visual inspection. Spectrum shows FTIR absorptions of slightly recrystallized CHA typical of archaeological bone lacking collagen [44].(B) Bone fragment of undetermined species showing brown discoloring. FTIR spectrum similar to (A) with the addition of a strong carbonate absorption at 1423cm^{-1} and weak calcite absorption at 713cm^{-1}. (C) Bone fragment of undetermined species showing black discoloring. FTIR spectrum similar to (A) with the addition of a weak peak at 1095cm^{-1} suggesting to be heated below 500°C [44]. (D) Fragment of human skull bone showing evidence of being calcined. FTIR spectra shows HA absorption at 635cm^{-1} and very weak

[1] One fragment of cemented wall of the pyre (Fig 11), one tooth and 21 bone fragments (fauna and human) were analyzed by Fourier transform infrared spectrometry (FTIR) to assess what temperature some of these materials retrieved from locus 338 were heated to (Table 1). By using FTIR it is in fact possible to assess if bone and tooth mineral and material containing clay minerals such as kaolinite and smectite were exposed to temperature above 500° C [43, 44]. Aliquot of a few micrograms of material were scraped from each sample, powdered and mixed with 5mg of KBr. Using a Pike™ hand-press the mixture was pressed into a 7mm die and analyzed with a Thermo Nicolet iS5 FTIR spectrometer. FTIR spectra were collected by performing 32 scans with a resolution of 4cm^{-1} wavenumbers. The FTIR spectra were analyzed using Omnic software and the SFU geo-archaeology laboratory FTIR spectral reference library.

carbonate absorptions at 875 and 1450cm^{-1}. This spectral characteristics are typical of bone mineral transformed to HA due to exposure with temperature comprised between 550°C and 900°C [44].

As far as the analysis of the fired mud brick material fragment that lined the pit, FTIR shows that the clay minerals normally composing the mud brick material of Beisamoun (Bocquentin et al., 2014) appears to be altered and missing the characteristic FTIR absorptions of Kaolinite and Smectite at 3695, 3620, 3650 and 915 cm^{-1} wavenumbers (Fig 11). This mineralogical composition supports the hypothesis that the mud brick material fragment found in L338 was heated to or above 700°C [43].

Fig 11. Pyre wall samples from Beisamoun, Locus 338. A. Picture of the fired mud brick material found in the cremation pit (see Fig 2) (Ref 3176-20; copyright L. Teira). B. FTIR spectra of sample 3176-20 (A) Greyish brown bulk material. The spectrum shows FTIR absorptions of quartz and calcite and a shoulder at 1050cm-1 assigned to vitrified clay minerals. This type of spectra are typical of local terra rossa soil heated above 700°C [43]. (B) Light tan material encrusting the depression of the grass-marks. FTIR spectrum shows the absorptions of quartz (779, 800 cm^{-1}), heated smectite (1045cm^{-1} and shoulder at 3620cm^{-1}), and calcite (713, 875, 1435 cm^{-1}).

In conclusion, the FTIR analysis shows that some of the human bone as well as the mud lining of the pyre were heated to temperatures well above 500°C, necessary for corpse cremation. This result is compatible with the colour and fissuration observed on the bones identified as calcinated. It is worth noting, on the otherhand, as suggested by the direct observation of the bones, the heterogeneous temperatures of heating and the presence of unheated bones.

3.4.2 Pyre layout and fuels

The south, east and west walls show significant rubefaction indicating that the cremation was carried out ~~on site~~. But these signatures of burning were not visible at the base of the pit during excavation. This phenomenon has already been described elsewhere: positioned at the base of the pyre, the fire only reaches a high temperature when it rises in an oxidizing environment [e.g.: 45]. An empty space ~~of any bone~~, located in the southern half of the pit, could correspond to the heart of the fire around which the bones are the most calcined. If our reconstruction of the position is exact, then the fire would have been placed under the flexed knees of the deceased. For the combustion to proceed properly, fuel and oxygen must be abundant throughout the cremation process. Bone can fuel the ongoing combustion, but cannot start it [e.g.: 39]. It is essential to use fuel, such as wood, to obtain and maintain a fire.

In order to investigate the fuels used for the cremations, phytolith analyses was performed on 10 sediment samples (eight in Locus 338, two outside the pyre as control samples: Fig 12). The pit samples yielded high densities of phytoliths[2] (Table 2). We found 'silica aggregate' remains of wood ash (Fig 13). Another notable plant category are phytoliths from sedge stems and leaves (Cyperaceae). Sedges are water-loving plant that have spongy stems and are commonly used for basketry and matting. We cannot exclude that a reed 'shroud' may have been used to wrap the corpse. The practice of wrapping corpses has been practiced in this area since the Natufian period and is quite common in Neolithic contexts all over the Levant [e.g. 13, 46].

---

[2] The sediments were first separated into aliquots of ca. 800 mg of sediment in the < 250 micrometer size fraction. These were treated with 10% HCl to remove pedogenic carbonates. We then added a dispersant of NaP to disaggregate clay particles. The fine sands and silts were settled out of the samples by allowing the material to fall through a column of water, after which we decanted the clay particles. The remaining sand and silt was dried, then burned in a muffle furnace at 500°C to remove organic matter. The sediment was then floated in a heavy density liquid which allowed the phytolith particles to float. Phytoliths were decanted, washed and mounted onto microscope slides. At least 300 phytoliths were counted for each sample.

Fig 12. **Spatial distribution of phytolith samples and context list**.

BEISAMOUN SAMPLES	Bei-14-1	Bei-14-2	Bei-14-3	Bei-14-4	Bei-14-5	Bei-14-6	Bei-14-7	Bei-14-8	Bei-14-9	Bei-14-10
**SINGLE-CELL**	**n /gm**	**n /gm**	**n /gm**	**n /gm**	**n /gm**	**n /gm**	**n /gm**	**n /gm**	**n /gm**	**n /gm**
Long-Cell (Psilate)	72473	101699	103346	138738	129430	87024	163947	257644	149205	140270
Long (Echinate)	18423	18734	55501	54731	62431	56368	77566	143461	70214	71729
Long (Sinuate)	3687	2676	6698	3818	1523	11867	22917	17567	8777	9564
Long-Cell (Cylindroid)	8595	3747	6698	8910	18272	16811	33495	32205	14628	3188
Long-Cell (Dendritic)	125303	25157	71768	96734	147702	75157	135741	184449	105321	98826
Papillae:	8595	1606	11483	1273	7614	5933	14103	17567	14628	4782
Stoma	1234	1071	0	1273	1523	0	0	0	0	0
Hairs:	7375	4282	1914	7637	15227	13845	14103	20494	11702	3188
Trichomes:	11062	5353	3828	10183	6091	8900	3526	8783	7314	6376
Bulliform:	33173	21410	14354	33093	21318	25712	35257	114183	27793	23910
Fan-shaped Bulliform	8595	6423	2871	14001	12182	7911	15866	52700	19016	12752
Crenates	12282	3212	10526	6364	6091	3956	14103	11711	11702	6376
Polylobes	0	0	1914	0	0	0	0	0	1463	3188
Bilobes:	1234	1606	9569	0	7614	3956	8814	0	10240	3188
Rondels:	25798	4817	39233	12728	57863	20767	42309	46844	33644	63759
Saddles:	11062	1071	12440	8910	12182	6922	8814	17567	8777	4782
Cones:	3687	0	0	2546	3045	3956	1763	0	1463	7970
Horned Tower:	1234	0	0	0	4568	1978	1763	0	0	0
Flat tower	0	1071	0	0	0	1978	1763	5856	0	0
Elongate psilate	1234	0	0	0	0	0	0	0	0	0
Echinate Spheroid	0	0	0	0	0	0	0	0	0	0
Globular psilate Spheroid	0	0	957	1273	1523	0	0	0	0	0
Sclereids	0	535	0	0	0	0	0	0	0	0
Tracheids	0	535	957	1273	0	989	0	2928	1463	1594
Blocks	3687	0	0	2546	1523	0	0	2928	0	0
Platelet	1228	1071	3828	8910	7614	0	19392	11711	4388	12752
Single Polyhedron	0	1071	957	1273	3045	1978	0	0	2926	0
Compound platelet	0	535	0	0	0	2967	0	0	0	1594
Silica aggregate	18423	5888	19138	61095	35022	4945	15866	158099	71677	47819
Coarse Verrucate	0	0	0	0	1523	0	0	0	0	1594
**MULTI-CELL**	**n per gm**	**n per gm**	**n per gm**	**n per gm**	**n per gm**	**n per gm**	**n per gm**	**n per gm**	**n per gm**	**n per gm**
Leaf/Stem:	0	0	11483	7239	0	0	2350	1403	0	0
LS Psilate	15304	6423	0	0	20699	19036	23505	26655	22277	17306
LS Sinuate	665	1071	478	0	1428	2596	1175	7014	1310	0
LS Echinate	4658	2676	957	0	4996	0	0	5612	1310	2472
LS Stomata	0	0	0	0	0	2596	3526	0	0	0
LS Saddle	0	0	0	0	714	0	0	0	1310	0
Panicoid leaf/stem	1331	3747	1435	2758	3569	1731	0	1403	1310	3708
Unident Husk:	1996	5353	1914	1379	6424	13845	9402	11223	10483	8653
Wheat Husk	1996	1606	957	689	2141	3461	2350	1403	0	0
Barley Husk	665	0	0	345	0	0	3526	0	0	3708
Aegilops	665	0	0	0	0	0	0	0	0	0
Wild Grass Husk:	13308	6423	4306	3103	15703	9518	15278	11223	5242	4945
Cyperus C- cones	3327	5888	2392	1379	4283	6922	4701	5612	2621	4945
Phragmites Stem	1331	2141	478	689	714	1731	3526	0	1310	1236
Juncus type	0	0	0	0	0	0	0	0	0	0
Panicoid leaf/stem	1331	3747	1435	2758	3569	1731	0	1403	1310	3708
Bromus-type Stem	0	0	0	689	0	0	0	1403	0	0
Avena	0	0	0	0	0	0	0	0	0	0
Setaria-Type Husk	0	535	478	0	0	0	8227	2806	9173	3708
Phragmites Leaf	3992	3212	1435	689	1428	1731	7051	0	3931	3708
Cyperaceae leaf/stem	13308	5888	4785	1724	5710	3461	8227	11223	5242	2472
Sedge stem	0	535	0	0	0	0	0	0	0	2472
Reed grass stem	0	0	0	689	714	0	1175	0	1310	0
Cereal Straw	0	1606	0	0	1428	865	1175	1403	0	0
Scirpus-type	0	0	0	0	0	865	0	1403	0	0
Multi-tiered forms	665	0	0	0	0	0	0	0	0	0
Polyhedron	2662	0	0	345	1428	3461	0	0	5242	2472
Polyhedral hair base	0	0	0	0	0	865	0	0	0	0
Verrucate	0	535	0	0	0	0	0	0	0	0
Coarse Verrucate	0	0	0	0	714	1731	0	1403	0	0
Awn	665	0	957	689	714	0	1175	0	0	1236
Cyperus sp.	0	0	0	0	714	0	0	0	0	0
Leaf/stem with irregular ronel/cone	0	0	0	0	0	0	2350	0	0	0
Leaf/stem jigsaw	0	0	0	0	0	0	1175	0	0	0
Square-cell leaf/stem	0	0	0	0	0	0	0	0	1310	0
Melted phytolith	0	0	0	689	0	0	0	0	0	0
Low conf. palm leaf	0	0	0	345	0	0	0	0	0	0
Low conf. wheat	665	535	0	0	0	0	0	0	0	0
Multiple square cells	0	0	957	0	714	1731	0	0	0	0
High conf. emmer	665	0	0	0	0	0	0	0	0	0

Table. 2. Raw Phytolith Counts in Numbers per Gram Sediment.

Fig 13. Diagrams of phytoliths outside (samples 14-1 and 14-2) and inside the cremation pit (all other samples). A: Graph showing densities of "silica aggregates" from the ash of wood from trees and shrubs. B: Graph showing concentrations of Sedge and Reed Grass (cf *Phragmites*) phytoliths. C: Graph showing concentrations of phytoliths from wheat. D: Phytolith densities of grass stems and grass florets indicating flowering grasses were a component of the burial.

In addition to sedges, we also found abundant concentrations of phytoliths from reed grasses in general and in particular *Phragmites* (common reed). These tall reeds grow along stream beds and around the shores of lakes and wetlands which characterized Beisamoun. Their stalks are strong and can be used for construction materials such as for fences, huts, roofing, as well as pallets for beds. The abundant remains of these reeds in the samples from the pyre could indicate that *Phragmites* stalks were used as additional fuel, but we cannot rule out the possibility that they might have been used as a pallet on which to rest the corpse. In addition to this evidence for possible sources of fuel, and possible methods for wrapping and treating the dead body before the cremation, we found that the florets of grasses were positively correlated with the stems of grasses, indicating the likelihood that grasses were placed into the pit as whole plants. The grasses could have been used as tinder for lighting the fire, or as some sort of ornamentation [47]. In either case, one of the important implications of this, is the likelihood that the cremation was conducted during the time of year that the grasses were flowering, for example in the late winter or spring. Finally, we found significant numbers of phytoliths from the husks of wheat, indicating that grains of wheat were present in the pyre, but also in the surrounding area. This could suggest that burial rituals, included the placing of foodstuffs in the cremation pit, although it is also possible that grains were a part of the general sediment matrix.

3.4.3 Pyre management and ~~remains manipulations~~

While part of the assemblage is highly fragmented, which is not surprising in a cremation context, it should be noted that several bones had retained their integrity or almost (right coxal, mandible - despite the fracturing of all teeth - metatarsals) and that the diaphyses of the long bones were relatively little fragmented. This may well be related to very limited combustion conduction with minimal technical intervention, leaving the corpse to burn at a slow rate [e.g. 20]. This would also explain the significant heterogeneity of combustion temperatures revealed by bone colour and FTIR investigation. It should be noted that, even without external intervention, under the action of fire, corpses are susceptible to trunk rotation and sometimes ~~spectacular~~ limb movements due to muscle contraction by heat [e.g. 37]. Subsequently, the gradual subsidence of the wood may have facilitated the dislocation of the skeleton. As they fell, the bones then spread around the hearth, further disrupting their initial position and orientation.

We observed five striae on the posterolateral face of the posterior talar facet of the right calcaneus (though the posterior talar facet was too fragmentary to be observed fully). These striae are wide, the longest are also the shallowest. The morphology of these striae more likely indicates the action of a carnivore rather than human intervention (Fig 14). In addition, on the edge with the sustentacular sulcus there is a small blunt and bright area, as if polished. The rest of the collection was meticulously observed a second time looking for additional traces on other bones, but without success. Although there is an animal burrow at the bottom of the pyre pit, it likely belongs to a rodent and not a carnivore. Therefore, the observed modification on the calcaneus should be considered as the result of an isolated event, more likely prior to cremation. A possible explanation could be a damage of the fresh foot by a carnivore on the corpse laid out and waiting to be cremated. The articulated phalanges of the right thumb found in the pyre ~~witnesses~~ nevertheless a short delay between death and cremation.

Fig 14. **Location of the striae and polish area observed on the right calcaneus**. Binocular acquisition made by A. Legrand.

3.5 The pit filling

Only one artefact was present in the pyre: a fragment of spatula, made of mammalian bone. Its fragmentary state does not favour an intentional deposit but the question remains open. A total of 776 faunal remains were recovered from cremation pit. The majority (NISP= 692) represent unidentified splinters of mammalian bone and tooth retrieved during sieving ~~such that they are extremely small~~. Only 84 remains could be identified to species and/or skeletal element. These are listed in Table 3 and represent at least eight taxa or faunal body size classes including common Levantine wild and domestic ungulates, birds of prey and freshwater fish.

**Species**	**NISP**
Cattle (*Bos* sp.)	7
Caprine (*Capra/Ovis* sp.)	2
Goat (*Capra* sp.)	2
Gazelle (*Gazella* sp.)	1
Pig (*Sus* sp.)	13
Large Mammal	5
Medium Mammal	39
Small Mammal	2
Raptor (Bird of prey)	1
Fish (Pisces sp.)	7
Cyprinids (e.g. Carp)	2
Cichlids (e.g. Tilapia)	3
**Total identified**	**84**
**Total unidentified frags**	692
**Total Fauna**	**776**

Table 3. Faunal taxa represented in cremation pit 338

The issue is whether the fauna recovered from the cremation pit were intentionally placed in the pit or are accidentally associated with the cremation (Fig 15). If intentional then they may have served as fuel for the cremation or represent grave offerings that were incinerated with the deceased. Alternately, they were simply part of the sedimentary matrix into which the cremation pit was dug, and so were accidentally burnt. Species identified in the pit, mirror those found in other parts of the site [25, 33, 34]. For remains that could be identified to skeletal element, a wide range of body parts are represented with no spatial discernible patterning (Table 4). Table 5 and Fig 16 present the data on colouration of all faunal remains recovered from the pit[3]. It is evident that the majority of remains

[3] Burnt animal remains may be the result of intentional burning resulting from cooking, refuse removal through burning in a fire, use of bone as fuel or due to intentional cremation [e.g. 48, 49, 50, 51, 52, 53, 54]. However, bones may also be burnt due to unintentional actions (e.g. post-depositionally), as demonstrated elsewhere [55, 56, 57, 58]. These studies have shown that bones lying underneath or adjacent to hearths or on cooled ashes may be altered in colour and texture, though apparently less so than bones that were in direct contact with a fire. Many methods are in use to determine burning. Here we have chosen three approaches- scoring colour [38, 56], bone size and type [e.g. 59, 60, 61] microscopic examination (x70) of the bone surface for alterations such as cracking [after 58] and FTIR analysis [62] to assess burning temperature. Experiments (e.g. 40, 55, 63] have shown that when burnt, dry bones tend to have lighter hue (brown shades)

show some degree of burning (black- black/grey classes). A total of 31% of the remains are unburnt (classes unburnt to brown/black in colour) while 22% are grey to white, which is the most similar colouration to the calcined human remains. Moreover, there was no patterning to the burning based on whether the skeletal element was an unidentified splinter or a diagnostic bone. In terms of their spatial arrangement, there are slightly more unburnt bones in the southern part of the pit than in the northern part, with a concentration of blackened bones towards the centre of the pit.

Fig 15. Spatial distribution of faunal remains from the pit according to colorimetric data.

**Element**	**NISP**
horncore	1
cranial	8
mandible	2
teeth	10
rib	11
vertebra#	17
scapula	3
humerus	2
radius	1
metacarpal	1
pelvis	1
femur	1
tibia	2
longbone shafts	15
calcaneum	1
carpals/tarsals	2
1st ph	4
2nd ph	2
**Total NISP**	**84**
# includes 12 fish vertebrae	

Table 4. Faunal skeletal element representation

**Colours**	**NISP**
Unburnt	91
Brown	107
Brown-Black	42
Black	178
Black-Grey	183
Grey-White	74
White	101
**Total NISP**	**776**

Table 5. Colour of faunal remains

Fig 16. ~~Pig diagram~~ showing breakdown of burning of faunal remains in the cremation pit.

compared to fresh bones, and exhibit less surface damage. Consequently, the degree of burning (colouration) may reflect the state of the bones when exposed to combustion.

It has been argued [e.g. 49, 61] that high proportions of small sized (<2 cm) burned fragments of spongy bone reflect its use as fuel. Most bones in the Beisamoun pit (NISP=636) represent very small fragments- both cortical and spongy bone- under 10mm in length, while 144 bones had lengths over 10mm (Fig 17A). Even so, the majority of bones are small and fall in the 10 to 30mm length range, with very few over 60mm in length. Likewise for breadth the majority of bones were under 5mm in width, while the larger remains peaked at 10 to 15mm (Fig 17B). As demonstrated in Table 6, there is a correlation between bone size (length, breadth, depth measurements) and colour, with the burnt bones e.g. ranging from black to grey, overall having smaller dimensions. Since burning makes bones more friable and so more susceptible to fragmentation [56, 60], this may account for the observed patterning rather than their use as fuel. Further evidence negating the use of bone as fuel in this pit is the low numbers of spongy bones in the assemblage as a whole, the presence of unburned spongy bone and the presence of burnt teeth.

Fig 17. Histograms of bone size (a) length (b) Breadth. Missing are bones <10mm in length and <5mm in breadth.

		NISP	L	B	D
Unburnt	X	40	31.78	15.05	10.44
	max		131.01	45.61	123.6
	min		11.51	5.63	2.42
Brown	X	21	31.82	14.91	8.40
	max		86.74	42.87	45.26
	min		12.27	5.56	2.31
Brown-Black	X	20	21.82	12.19	6.60
	max		37.08	19.82	12.52
	min		12.39	4	2.68
Black	X	27	20.86	10.48	5.73
	max		51.17	24.82	17.82
	min		10.28	4.67	2.47
Black-Grey	X	16	20.82	9.23	6.13
	max		43.85	13.96	10.34
	min		10.62	3.91	2.98
Grey-White	X	15	19.62	10.44	6.48
	max		24.33	15.78	19.8
	min		12.03	6.95	2.64

Table 6. Colour-size correlation

The identified bones and a sample of 50 unidentified fragments were subjected to a microscopic examination of the surface texture at x70 magnification. Cracks and exfoliation on the cortical surface were only observed on the calcined bones. Pérez et al. [58] noted that fresh bone exhibits extensive changes to the cortical surface (in color, a high degrees of fragmentation, cracks and exfoliation =structural changes on the cortical surface), while dry bone shows only changes in color. If, the bones in the pit were dry when burnt, this may explain the absence of alterations to the bones surface as well as the more minor burning relative to the human remains. Experiments on modern

bone used a fuel demonstrate that spongy bones burn better than dry bones and compact bone without the cancellous portion [48, 49, 60, 61, 64]. Thus, if bone was used for fuel, spongy bones should dominate the burnt assemblage. Examination of the relative frequency of cortical shafts to trabecular-rich bones (spongy bones) in the cremation pit (Table 4), shows that only 34.5% represent spongy bones (ribs, vertebrae, horncore). Moreover, as demonstrated above, the vast majority of the faunal remains exhibit a far lower degree of burning than the calcined and cremated human remains. Consequently, it is highly unlikely that the spongy bones found in the pit served as fuel for the cremation.

Non-anthropogenic modifications were observed on only two bones; small carnivore puncture holes were evident on a humerus shaft and on an unidentified large mammal shaft fragment (Fig 18A). In addition, a percussion fracture was evident on the same humerus shaft as noted above (Fig 18B) and another shaft fragment exhibited a flaked end. This random representation of damage to the bones further supports the faunal assemblage as part of the background fill of the pit.

Fig 18. Non-anthropogenic modifications observed on faunal assemblage. (a) Large mammal bone fragment with carnivore tooth pits. (b) Large mammal humerus shaft with a percussion fracture.

To conclude, the data presented here on the animal remains from Locus 338 all point to their representing accidental inclusions, remains that were already present in the sediment into which the pit was dug. This is based on several factors: (i) the lack of evidence for selection of specific taxa and/or skeletal elements since a wide spectrum of both are represented, and all taxa are found in other parts of the site; (ii) the absence of any discernible spatial structure in the location of the faunal remains within the pit –they are dispersed throughout; (iii) the small size of most of the remains – with the majority representing unidentified splinters less than 10mm in dimension- suggests a refuse deposit; (iv) the uneven pattern of burning -ranging from unburnt to calcined – and relative paucity of bones burnt to the same degree as the human remains – would rather reflect the proximity of the remains to the cremation rather than being the result a single combustion event. The range of colours and the low frequency of spongy parts, also negates the use of bones as fuel; (v) the low relative absence of structural changes to the cortical bone surface on most bones, suggests that the majority were already dry when burnt-which may also account for the lower degree of burning observed. (vi) the presence of disparate taphonomic modifications on the bones including carnivore and human damage, indicate different agents of modification and so origins. Consequently, the animal remains do not present a cohesive assemblage and as such do not form an integral part of the cremation or rites associated with it.

3.6 Abandonment of the pyre

Eroded throughout its northern upper half about 15 to 20 cms, the pit is entirely preserved at its southern end. The lower two-thirds are filled with ash sediment, in which the human remains were found (Fig 19). Above the pit, in the upper 20 to 25 cm, is a sediment identical to the surrounding matrix (Ib). Some segments of the pit wall collapsed inside the structure, sealing the layer of ash and bone (Fig 2). This suggests that once the cremation was completed, the structure was left abandoned as it was, open. This hypothesis is reinforced by microstratigraphic analyses carried out on several samples of the wall, which show that its upper part has been strongly altered by weathering. The removal of bones after cooling cannot be excluded (which may explain why the skeleton is not complete). In addition, the presence in Beisamoun of secondary deposits of burnt bones attests to a tradition of selective collection destined to be buried elsewhere (see below). But in case of pyre 338, it is difficult to separate what was taken away from what was dispersed by the fire, destroyed later on or taken away by the animal that inhabited the burrow.

Fig 19. West section of the south-east fourth of the pit. Ongoing excavation. Note the ashy sediment at bottom sealed by Layer Ib matrix.

4. Other Cremations at Beisamoun

To date, five other cremations are known on the site (2 in sector F and 3 in sector E). Amongst them three were excavated and other were found in the last excavation season and left in situ (locus 460 was partly excavated). All of them are secondary deposits of selected cremated bones collected together with some ashes of the pyre. Their respective inventories show that all categories of bones were selected, in small quantities (loci 246 and 347 were fully excavated; 211 was partly destroyed by a mechanical shovel) (Fig 20). For ~~all of them~~ the MNI is one adult and the bones show signs of primary cremation of a fresh corpse. The bones of Locus 347 delimited a partial circle, attesting that they were likely deposited in a container, possibly a basket [25: fig. 32]. In locus 211 a worked, perforated and burnt limestone cylinder was deposited with the cremated human remains [25: fig. 25]. These cremations are all contemporaneous with other types of funerary treatment of ~~adult~~ ~~deceased~~ at the site. ~~At the contrary~~, immature individuals are buried the same way (primary deposit) during the whole occupation sequence.

Fig 20. ~~Conservation~~ sheets of the loci 211, 246, 347 from Beisamoun, three secondary deposits of selected cremated remains (MNI is one adult respectively).

Discussion

The Beisamoun Locus 338 is a cremation pit that served ~~as well~~ as a final repository for the cremated corpse. What remains of the deceased allows us to affirm that it was a relatively fresh corpse that has been burnt. The bones and the structure exhibit signs of combustion at a high temperature (well adapted to the intention to remove flesh) on fresh bone tissue (distortion, splitting, longitudinal cracking). Overall, some bones have retained their anatomical coherence, including labile joints (feet phalanges; ribs/vertebrae; shoulder/arm left). The body was possibly seated, its back against the south or south-west wall of the structure. This hypothesis will have to be strengthen by new cases in the future. Phytoliths are numerous and testify to the presence of wood fuel. Sedges and reed grass possibly formed part of a funerary bed or a funerary container. The pit was constructed using a plaster-like material common in other dwelling features at the site. Bones and ashes were left in place and later enclosed by the collapse of the burnt pit wall.

The site occupation in this sector recently excavated spans 7200 to 6400 cal BC but ending possibly a bit later as no C14 dates were yet obtained for the most recent levels. The occupation might indeed cover the end of the Late PPNB (not precisely dated) and the whole Final PPNB/PPNC entity. Throughout this sequence, the lithic toolkit composition shows little variation and retains traits of the previous PPN tradition based on bidirectional technology [65]. We know from previous research at the site and the two deep trenches we dug, that the site was occupied from at least the Middle PPNB.

Two of the six cremation deposits were directly dated. The calcinated fibula of skeleton cremated in pyre 338 (Layer Ib sector E) was dated to 7031-6700 cal BC (Beta-514789; 7950±30 BP; 95,4 % of confidence BetaCal3.21); the adult from locus 211 (Upper Layer of sector F) was dated by its calcinated femur at 7084-6831 cal BC (Beta-519962; 8060±30 BP; 97.7% of confidence BetaCal3.21). According to our stratigraphic observations, the four other cremated loci should be contemporaneous or slightly earlier. It means that cremation treatment appears at Beisamoun with the transition from the 8th to the 7th millennia BC. Immediately before, adults are found in primary or secondary deposit and experience skull removal and plastering (two ~~much~~ damaged plastered skulls were found in the

2016 excavation season).With the appearance of cremation, primary and secondary are maintained but skull removal and plastering seem to disappear. After a cohabitation of few centuries, inhumation and cremation both disappear on site and there were only a few child burials interred in the site before its definitive abandonment. Cremation practice seems consequently quit ephemeral locally.

To date, the cremations at Beisamoun and the pyre structure are possibly the most ancient of the Near East. Strickingly similar cremation pits that were mud-plastered, were found at the later site of Tell-el-Kherk in the Northern Levant that have been attributed to the Middle Pottery Neolithic level dated between 6600 and 6100 cal BC [7, 23, 66]. However, they differ notably in their functioning: remains of all ages, including infants, were found and most of them were used to inter several deceased, up to ten in number. This quantity of individuals packed in structures not larger (about 1 m diameter) than locus 338 of Beisamoun convinced the authors that that the deceased must have been ~~cremated~~ ~~already~~ skeletonized or at least decomposed (some joints are indeed still articulated). Moreover, mostly skulls and long bones were present. A detailed anthropological analyses would probably be necessary to arrive at some conclusions regarding the chaine operatoire in this case but the fact that the bones are found almost complete, with transversal break and a majority do not seem calcinated but only carbonnized (from picture observation) would indeed support the hypothesis of secondary cremations[4]. A few centuries later, during the 6th millennium in Halaf contexts, cremation and cremation pits are also documented in ~~site~~ further to the east such as Yarim Tepe II [67] and further to the north-west at Mesrin [68]. In the Southern Levant, charred bones are mentioned in one PPNC grave at Ashkelon but considered as part of secondary disturbances [69 p. 245]. In the other Southern Levantine sites with several dozens of FPPNB/PPNC graves ('Aïn Ghazal; Atlit Yam; Motza) cremation practice is as yet, not documented. This would suggest that cremation emerged in the Central Levant and spread to the Northern Levant where large cemeteries appear in parallel. At Beisamoun, ~~at~~ the contrary, cremation ~~is just preceding~~ the end of the local millennial practice of burying the dead near houses (at least a selected part of the dead). Indeed, Pottery Neolithic burials in general are very scarce in the Southern Levant and cemeteries are yet unknown witnessing a clear shift in ~~dead treatment~~ and possibly shift in ~~believes.~~ According to the data available from Beisamoun, this shift in adult funerary handling could have taken place before the end of the FPPNB/PPNC, approximately at 6500 cal BC.

In conclusion the emergence of cremation at the end of the 8th millennium could be contemporaneous with the very beginning of the FPPNB/PPNC, a major transitional break that is still poorly understood. The cremation activities demonstrate the practice of an alternative funerary program which might have had a strong impact on funeral procedure, mourning time and even ritual meaning. The pyre structure found at Beisamoun, together with the secondary deposits, show a complex operational chain of funerary ~~gesture~~ with strong technical constraints and several steps of handling. This involvement contrasts with other cultural aspects which seem to be technically under invested when compared to previous time periods (e.g. limeplaster manufacture, house building, flint knapping). Moreover, the Central Levant seems to be an innovative centre in terms of funerary treatment which may have spread towards the north. Indeed the similarity of the cremation pits at Tell-el-Kherk makes coincidence unlikely, even if the practice appears to have evolved and changed. It is an argument to think that some South-North Levantine connection did continue at the end of the Late PPNB despite observed new ~~divergence~~ in lithic production and the lag in the introduction/development of pottery manufacture in the southern Levant.

Acknowledgments:

[4] Some anthropologists is this case may refute the term « cremation » as no attempt is made here of active defleshing by fire. However, the deliberate treatment of the remains by fire at Tell-el-Kherk is obvious considering the pyre structures and the number of skeletons involved and should be distinguished from other accidental burning as well as all cases where the human remains are ~~burn~~ as a consequence but not as first intention (destruction of grave structure by fire, proximity with fire place etc…[see for instance 15]).

Simon Dorso and Caroline Masset were patient enough to excavate the cremation pit with all the meticulous attention required. Cécile Berton and Rachel Jadaud participated to the preparation of the bones (cleaning, restoration, refitting). The Ecole Biblique et Archéologique Française de Jérusalem generously hosted us during part of the lab work; we are especially grateful to J.-B. Humbert who gave us assistance since the beginning of the project and a warm welcome. Alexandra Legrand, Yolanda Fernandez-Jalvo and Ferran Borrell offered us advice concerning the striae observed on the calcaneus and the projectile embedded in the scapula. Luis Teira (Universitad de Cantabria), Alexandra Legrand (USR 3225), Rémi Brageu (LAMS) for their excellent micro and macroscopic photography. X-ray and 3D imaging were performed by Denis Belœil and Fabrice Edon at the Service d'Imagerie médicale du Centre du Moulinet, Paris 13ème, under the supervision of Laurent Buisson and Dr. Eric Auberton who kindly give us their opinions on the injured shoulder. We are grateful for their availability.

References:

[1] Coqueugniot E, Aurenche O, editors. Néolithisations : nouvelles données, nouvelles interprétations. À propos du modèle théorique de Jacques Cauvin. Paléorient (Special Issue). 2011;37. Available from: https://www.persee.fr/issue/paleo_0153-9345_2011_num_37_1

[2] Benz M. Beyond death - the construction of social identities at the transition from foraging to farming. In: Benz M, editor. The Principle of Sharing Segregation and Construction of Social Identities at the Transition from Foraging to Farming. Berlin: Ex-Oriente; 2010. pp. 249–276.

[3] Croucher K. Death and dying in the Neolithic Near East. Oxford: Oxford University Press; 2012.

[4] Santana J, Velasco J, Ibáñez JJ, Braemer F. Crania with mutilated facial skeletons: A new ritual treatment in an early Pre-Pottery Neolithic B cranial cache at Tell Qarassa North (South Syria). American Journal of Physical Anthropology. 2012;149: 205-216. doi:10.1002/ajpa.22111

[5] Ortiz A, Chambon P, Molist M. "Funerary bundles" in the PPNB at the archaeological site of Tell Halula (middle Euphrates valley, Syria): analysis of the taphonomic dynamics of seated bodies. Journal of Archaeological Science. 2013;40: 4150–4161. doi:10.1016/j.jas.2013.06.014

[6] Erdal YS. Bone or Flesh: Defleshing and Post-Depositional Treatments at Körtik Tepe (Southeastern Anatolia, PPNA Period). European Journal of Archaeology. 2015;18: 4–32. doi:10.1179/1461957114Y.0000000072

[7] Chamel B. Bioanthropologie et pratiques funéraires des populations néolithiques du Proche-Orient : l'impact de la Néolithisation : étude de sept sites syriens – 9820-6000 cal. BC. PhD thesis, Université de Lyon 2. 2014. Available from: http://www.theses.fr/182102378

[8] Khawam R. L'Homme et la mort au néolithique précéramique B : l'exemple de Tell Aswad. PhD thesis, Université de Lyon 2. 2014. Available from: http://www.theses.fr/2014LYO20133

[9] Grosman L, Munro ND, Abadi I, Boaretto E, Shaham D, Belfer-Cohen A, et al. Nahal Ein Gev II, a Late Natufian Community at the Sea of Galilee. PLOS ONE. 2016;11. doi:10.1371/journal.pone.0146647

[10] Bocquentin F, Kodas E, Ortiz A. Headless but still eloquent! Acephalous skeletons as witnesses of Pre-Pottery Neolithic North-South Levant connections and disconnections. Paléorient. 2016a;42(2):33–52. doi:10.3406/paleo.2016.5719

[11] Garrod DAE. Excavations in the Caves of the Wadi-el Mughara, 1931. Bulletin of the American School of Prehistoric Research. 1932;8: 6-11.

[12] Noy T. Some aspects of Natufian mortuary behavior at Nahal Oren. In: Hershkovitz I, editor. People and culture in change : proceedings of the second symposium on Upper Palaeolithic, Mesolithic and

Neolithic populations of Europe and the Mediterranean Basin ; Part 1. Oxford: British Archaeological Reports; 1989.

[13] Bocquentin F. Burial practices, biological factors and cultural identities during the Natufian period : a bio-archaeological prespective. PhD. Thesis, Université Bordeaux 1. 2003. Available from: https://hal-univ-paris10.archives-ouvertes.fr/tel-01997088.

[14] Valla F, Khalaily H, Valladas H, Tisnerat-Laborde N, Samuelian N, Bocquentin F, et al. Les fouilles de Mallaha en 2000 et 2001: 3ème rapport préliminaire. Journal of the Israel prehistoric society. 2004;34: 49–244.

[15] Le Mort F, Erim-Özdögan A, Özbek M, Yilmaz Y. Feu et archéoanthropologie au Proche-Orient (Epipaléolithique et Néolithique). Le lien avec les pratiques funéraires. Données nouvelles de Çayönü (Turquie). Paléorient. 2001;26: 37-50. doi:10.3406/paleo.2000.47081.

[16] Stordeur D, Khawam R. Les crânes surmodelés de Tell Aswad (PPNB, Syrie). Premier regard sur l'ensemble, premières réflexions. Syria (Archéologie, art et histoire). 2007;84 : 5–32. doi:10.4000/syria.321

[17] Bar-Yosef O, Sillen A. Implications of the New Accelerator Date of the Charred Skeletons from Kebara Cave (Mt. Carmel). Paléorient. 1993;19: 205–208. doi:https://doi.org/10.3406/paleo.1993.4591

[18] Hertz R. Contribution à une étude sur la représentation collective de la mort. In: Hertz R, editor. Sociologie religieuse et folklore. Paris: Les Presses universitaires de France (Bibliothèque de sociologie contemporaine);1907 [1928, 1970]. Available from: http://classiques.uqac.ca/classiques/hertz_robert/socio_religieuse_folklore/socio_religieuse_.html

[19] Thomas L-V. Le cadavre: de la biologie l'anthropologie. Bruxelles: Editions Complexe; 1980.

[20] Le Goff I. Cadavre et crémation. In: Guy, H, Jeanjean A, Richier A, (editors). Le Cadavre en procès, Techniques &Culture. 2013;60: 92-109.

[21] Kuijt I, Quinn CP, Cooney G, editors. Transformation by fire: the archaeology of cremation in cultural context. Tucson: The University of Arizona Press; 2014.

[22] Bachelot L, Tenu A, editors. Entre mondes orientaux et classiques : l'incinération dans les cultures syro-anatoliennes. Ktéma. 2005;30: 5-204.

[23] Tsuneki A. A glimpse of human life from the Neolithic cemetery at Tell el-Kerkh, Northwest Syria. Documenta Praehistorica. 2011;38: 83-95.

[24] Milevski I, Bocquentin F, Molist M, editors. North-South Connections and Disconnections in the Prehistory and Proto-history of the Levant. Paléorient. 2016; vol. 42. Available from: https://hal-univ-paris10.archives-ouvertes.fr/hal-01996652

[25] Bocquentin F, Khalaily H, Bar-Yosef Mayer D, Berna F, Biton R, Boness D, et al. Renewed Excavations at Beisamoun: Investigating the 7th Millennium cal. BC of the Southern Levant. Journal of the Israel prehistoric society. 2014;44: 5-100.

[26] Rollefson G, Kohler-Rollefson I. PPNC adaptations in the first half of the 6th millennium b.c. Paléorient. 1993; 33–42.

[27] Kuijt I. People and Space in Early Agricultural Villages: Exploring Daily Lives, Community Size, and Architecture in the Late Pre-Pottery Neolithic. Journal of Anthropological Archaeology. 2000;19: 75-102. doi:10.1006/jaar.1999.0352

[28] Bar-Yosef O, Bar-Yosef Mayer D. Early Neolithic Tribes in the Levant. In: Parkinson WA, editor. The Archaeology of Tribal Societies. Ann Arbor, Michigan: International Monographs in Prehistory; 2002. pp. 340–371.

[29] Simmons AH. The Neolithic Revolution in the Near East: Transforming the Human Landscape. Tucson, Ariz: University of Arizona Press; 2011.

[30] Smith P, Horwitz L. Ancestors and inheritors: A bio-cultural perspective of the transition to agro-pastoralism in the Southern Levant. In: Cohen MN, Crane-Kramer GMM, editors. Ancient health: skeletal indicators of agricultural and economic intensification. Gainesville: University Press of Florida; 2012. pp. 207-222.

[31] Bocquentin F, Chamel B, Anton M. Subsistence and foodways transition during Neolithization process: glimpses from a contextualized dental perspective. Food & History. Forthcoming.

[32] Lechevallier M. Abou Gosh et Beisamoun. Paris: Association Paléorient (Mémoires et Travaux du Centre de Recherche Français de Jérusalem); 1978.

[33] Bocquentin F, Khalaily H, Samuelian N, Barzilai O, Le Dosseur G, Kolska Horwitz L, et al. Renewed excavation on the PPNB site of Beisamoun, Hula Basin. Neo-Lithics: The Newsletter of Southwest Asian Neolithic Research. 2007;(2):17–21.

[34] Bocquentin F, Barzilai O, Khalaily H, Horwitz LK. The PPNB Site of Beisamoun (Hula Basin): Present and Past Research. In: The state of the stone: Terminologies, Continuities and Contexts in Near Eastern Lithics. Berlin: Ex-Oriente; 2011. p. 197–211. (Studies in early Near Eastern production, subsistence, and environment).

[35] Schaefer M, Black S, Sheuer L. Juvenile Osteology. A Laboratory and Field Manual. Elsevier/Academic Press; 2009.

[36] McKinley JI. Bone fragment size and weights of bone from modern British cremations and the implications for the interpretation of archaeological cremations. International Journal of Osteoarchaeology. 1993;3: 283-287.

[37] Depierre G. Crémation et archéologie. Nouvelles alternatives méthodologiques en ostéologie humaine. Dijon : Éditions Universitaires de Dijon, Collection Art, Archéologie & Patrimoine ; 2014.

[38] Shipman P, Foster GF, Schoeninger M. Burnt bones and teeth: an experimental study of color, morphology, crystal structure and shrinkage. Journal of Archaeological Science 1984;11: 307-325.

[39] Susini A. Étude des caractéristiques biophysiques des tissus calcifiés humains soumis à des traitements thermiques : Applications anthropologiques et médicales. Université de Genève; 1988.

[40] Buikstra JE and Swegle M. Bone Modification Due to Burning: Experimental Evidence. In: Bonnichsen R, Sorg MH, editors. Bone Modification. Orono, ME: Center for the Study of the First Americans. 1989. pp. 247-258.

[41] Mayne Correia P. Fire Modification of Bone: A Review of the Literature. In: Haglund WD, Sorg MH, editors. Forensic Taphonomy: The Postmortem Fate of Human Remains. Boca Raton: CRC Press; 1997. pp. 275-293.

[42] McKinley JI. The analysis of cremated bone. In: Cox M, Mays S, editors. Human osteology in archaeology and forensic science. London: Greenwich Medical Media; 2000.pp 403-421.

[43] Berna F, Behar A, Shahack-Gross R, Berg J, Zorn ., Boaretto E, Gilboa A, Sharon I, Shalev S, Silshtein S, Weiner S. Sediments Exposed to High Temperatures: Reconstructing Pyrotechnological Practices in Late Bronze and Iron Age Strata at Tel Dor (Israel). Journal of Archaeological Science. 2007;34: 358-373.

[44] Berna F, Bone alteration and diagenesis. In: Artioli G, editor. Scientific Methods and Cultural Heritage. An Introduction to the Application of Materials Science to Archaeometry and Conservation Science. Oxford: Oxford University Press; 2010. pp. 364-367.

[45] Blaizot F. Contribution à la connaissance des modes de dislocations et de destruction du squelette pendant la crémation : l'apport du bûcher funéraire en fosse du Néolithique final à Reichstett-

Mundolsheim (Bas-Rhin). Bulletins et Mémoires de la Société d'Anthropologie de Paris. 2005 ;17(1-2) : 13-35.

[46] Boyd B. Ecologies of fiber-work: Animal technologies and invisible craft practices in prehistoric Southwest Asia. Quaternary International. 2018;468: 250–261. doi:10.1016/j.quaint.2017.06.050

[47] Nadel D, Danin A, Power R, Rosen A, Bocquentin F, Tsatskin A, et al. Earliest floral grave lining from 13,700–11,700-y-old Natufian burials at Raqefet Cave, Mt. Carmel, Israel. Proceedings of the National Academy of Sciences. 2013;110: 11774–11778. doi:10.1073/pnas.1302277110

[48] Costamagno S, Théry-Parisot I, Brugal JP, Guibert R. Taphonomic consequences of the use of bones as fuel. Experimental data and archaeological applications. In: O'Connor T, editor. Biosphere to Lithosphere: New Studies in Vertebrate Taphonomy. Oxford: Oxbow Books; 2005. pp. 51-62.

[49] Théry-Parisot I, Costamagno S, Brugal JP, Fosse P, Guilbert R. The use of bone as fuel during the Palaeolithic, experimental study of bone combustible properties. In: Mulville J, Outram A, editors. The Zooarchaeology of Fats, Oils, Milk and Dairying. Oxford: Oxbow Books; 2005. pp. 50-59. ⟨halshs-00008489⟩

[50] Asmussen B. Intentional or incidental thermal modification? Analysing site occupation via burned bone. Journal of Archaeological Science. 2009;36(2): 528–36.

[51] Bosch MD, Nigst PR, Fladerer FA, Antl-Weiser W. Humans, bones and fire: Zooarchaeological, taphonomic, and spatial analyses of a Gravettian mammoth bone accumulation at Grub-Kranawetberg (Austria). Quaternary International. 2012;252: 109-121.

[52] Yravedra J, Uzquiano P. Burnt bone assemblages from El Esquilleu cave (Cantabria, Northern Spain): deliberate use for fuel or systematic disposal of organic waste? Quaternary Science Reviews. 2013; 68: 175-190.

[53] Fulton DN, Hesse P, Hesse B. Considering Carcasses: Sheep and Goat Sacrifice at Carthage, Tunisia, and Al Qisha, Yemen. In: Lev-Tov J, Hesse P, Gilbert A, editors. The Wide Lens in Archaeology: Honoring Brian Hesse's Contributions to Anthropological Archaeology. Lockwood Press; 2017. pp. 245-256.

[54] Carroll EL, Smith M. Burning questions: Investigations using field experimentation of different patterns of change to bone in accidental vs deliberate burning scenarios. Journal of Archaeological Science: 2018;20 : 952-963.

[55] Bennett JL, Thermal alteration of buried bone. Journal of Archaeological Science. 1999;26: 1-8.

[56] Stiner MC, Kuhn SL, Weiner S, Bar-Yosef O. Differential burning, recrystallization,and fragmentation of archaeological bone. Journal of Archaeological Science. 1995 ;22(2): 223-237.

[57] Cain CR. Using burned animal bone to look at Middle Stone Age occupation and behavior. Journal of Archaeological Science. 2005;32: 873-884.

[58] Pérez L, Sanchis A, Hernández CM, Galván B, Sala R, Mallol C. 2017. Hearths and bones: An experimental study to explore temporality in archaeological contexts based on taphonomical changes in burnt bones. Journal of Archaeological Science. 2017;Reports 11: 287-309.

[59] Villa P, Bon F, Castel JC. Fuel, fire and fireplaces in the Palaeolithic of Western Europe. Review of Archaeology. 2002;23(1): 33-42.

[60] Villa P, Castel JC, Beauval C, Bourdillat V, Goldberg P. Human and carnivore sites in the European Middle and Upper Paleolithic: similarities and differences in bone modification and fragmentation. Revue de Paléobiologie. 2004;23(2): 705-730.

[61] Théry-Parisot I. Fuel management (bone and wood) during the Lower Aurignacian in the Pataud rock shelter (Lower Palaeolithic, Les Eyzies de Tayac, Dordogne, France). Contribution of experimentation. Journal of Archaeological Science. 2002;29(12): 1415-1421.

[62] Berna F, Goldberg P, Horwitz L.K, Brink J, Holt S, Bamford M, Chazan M. Microstratigraphic Evidence of *in situ* Fire in the Acheulean Strata of Wonderwerk Cave, Northern Cape Province, South Africa. PNAS. 2012;109(20): E1215–E1220.

[63] Whyte T. Distinguishing remains of human cremations from burned animal bones. Journal of Field Archaeology. 2001;28(3/4): 437-448.

[64] Costamagno SC, Griggo C, Mourre V. Approche expérimentale d'un problème taphonomique: utilisation de combustible osseux au Paléolithique. Préhistoire européenne. 1999;13: 167-194.

[65] Borrell F, Bocquentin F, Gibaja J, Khalaily H. Defining the Final PPNB/PPNC in the Southern Levant: insights from the chipped stone industries of Beisamoun. In: Briois F, Astruc L, McCartney C, editors. 8th Conference on PPN Chipped and Ground Stone Industries of the Near East Nicosia University of Cyprus 23-27th November 2016. In Press.

[66] Tsuneki A, editor. Life and Death in the Kerkh Neolithic Cemetery. University of Tsukuba and DGAM Archaeological Mission to Tell el-Kerkh; 2011.

[67] Merpert NY, Munchaev RM. Chapitre 10. Burial Practices of the Halaf Culture. In: Yoffee N, Clark JJ, editors. Early Stages in the Evolution of Mesopotamian Civilization Soviet Excavations in Northern Iraq. Tuscon & London: The University of Arizona Press; 1993. pp. 207–223.

[68] Akkermans P. Halaf Mortuary Practices: A Survey. In: Haex OMC, Curvers HH, Akkermans PMMG, editors. To the Euphrates and Beyond Archaeological Studies in Honour of Maurits N van Loon. Rotterdam: CRC Press; 1989. pp. 75–88.

[69] Faerman M, Smith P. Chapitre 14. Anthropological and taphonomic analyses of skeletal remains. In: Garfinkel Y, Dag D, editors. Neolithic Ashkelon. Jerusalem: Institute of Archaeology of the Hebrew University, Qedem Monographs 47; 2008. pp. 237–248.

Click here to download Figure Fig1.tif

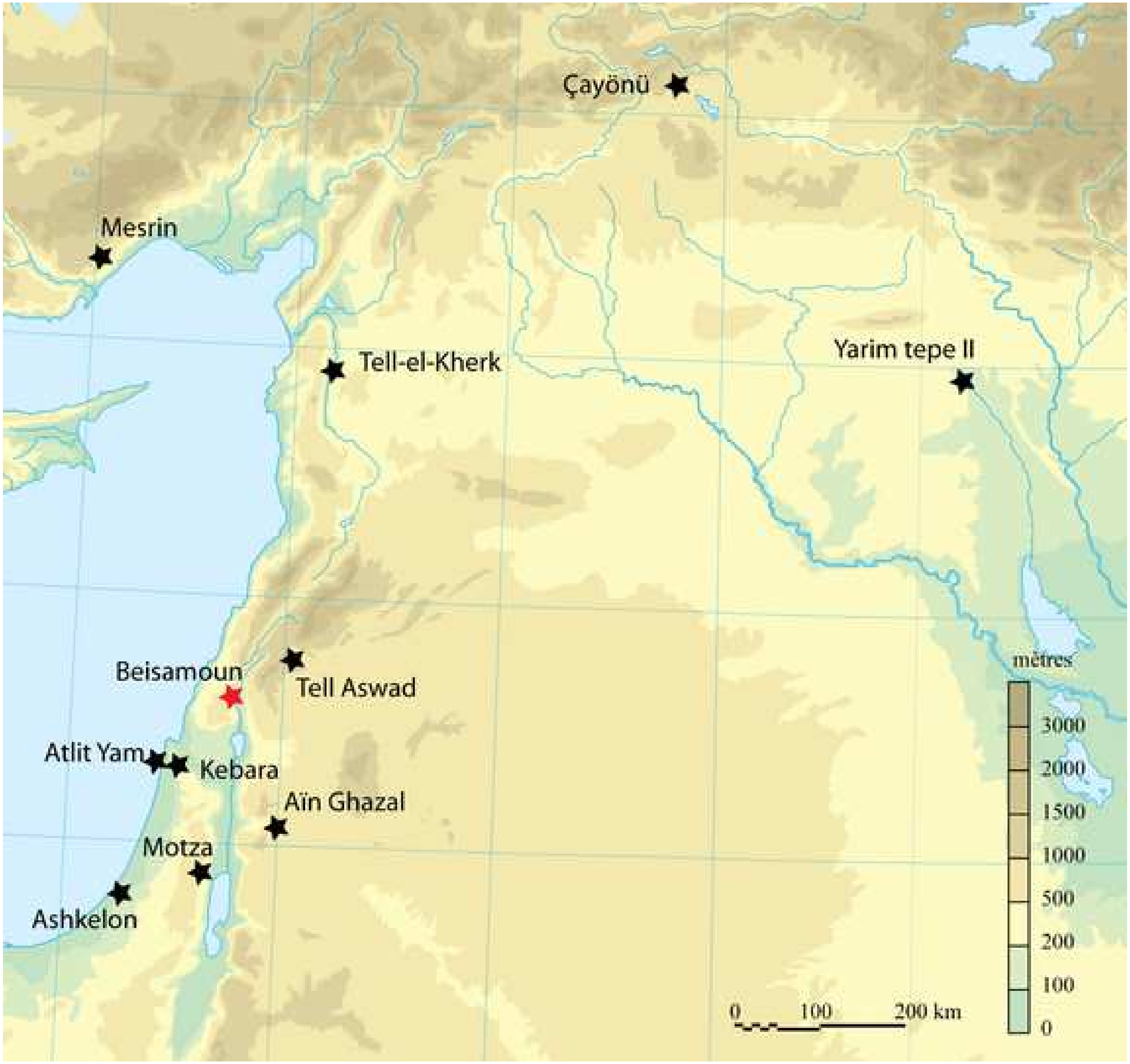

Click here to download Figure Fig2.tif

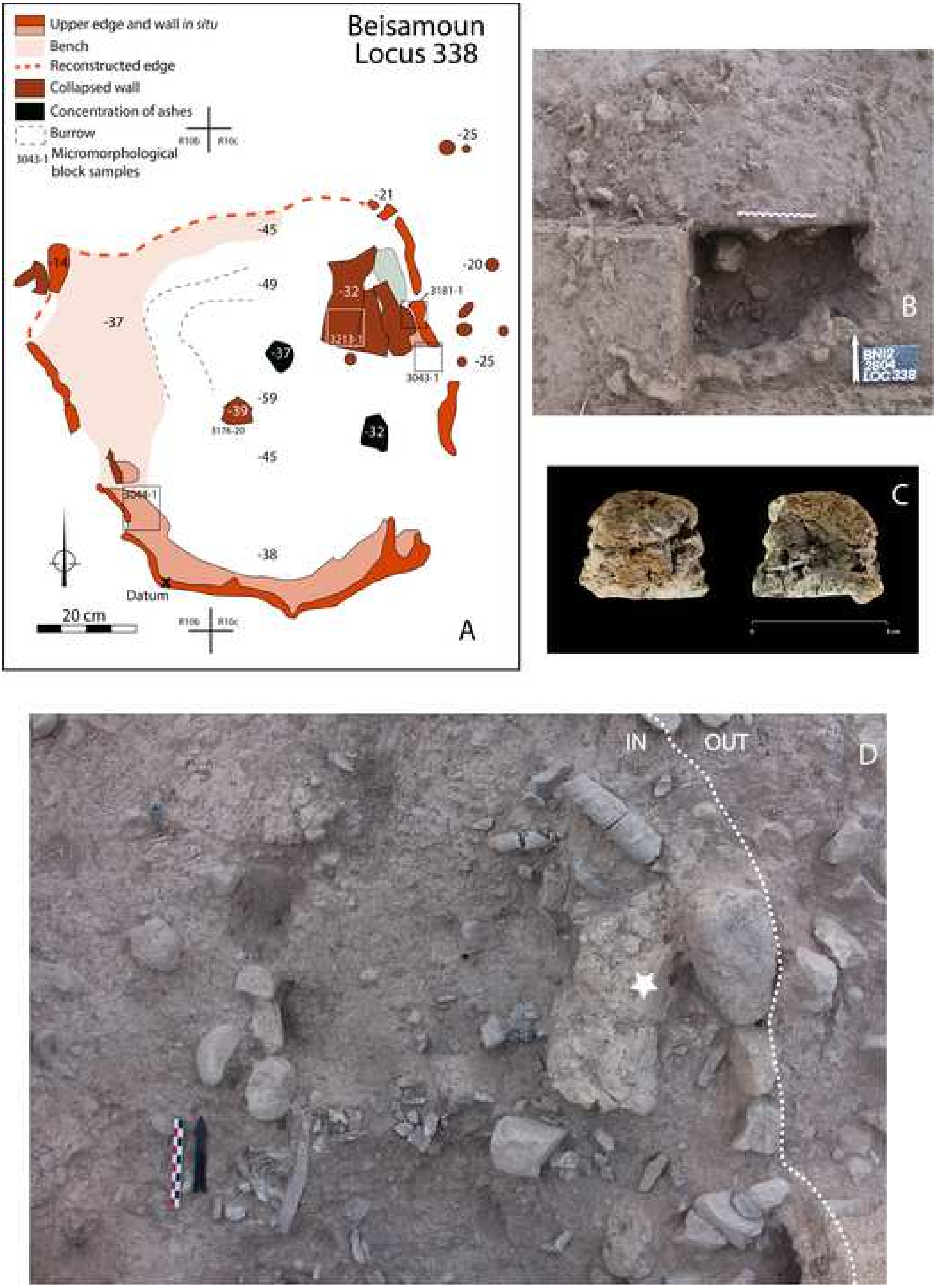

Figure

Click here to download Figure Fig3.tif

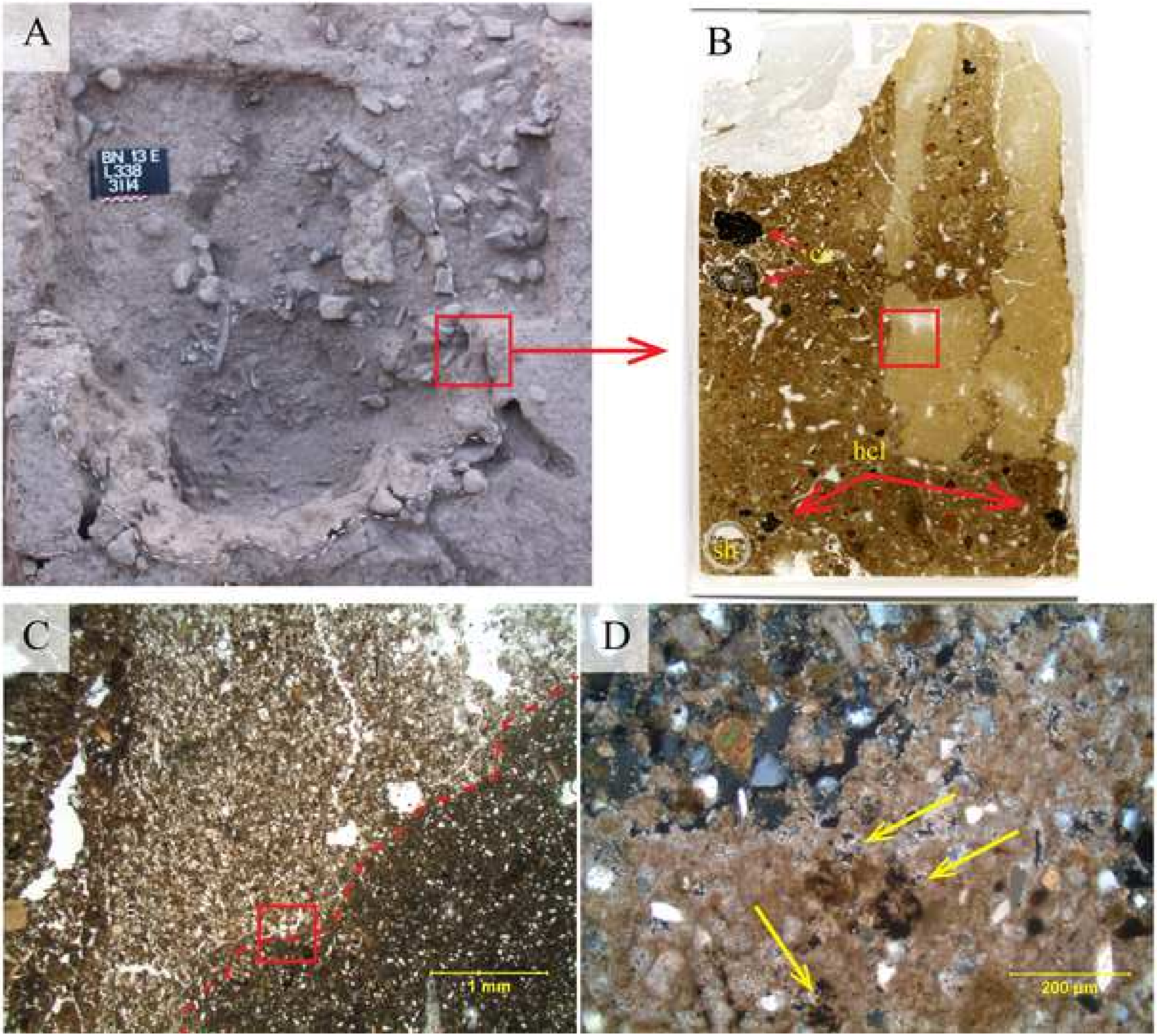

Click here to download Figure Fig4.tif

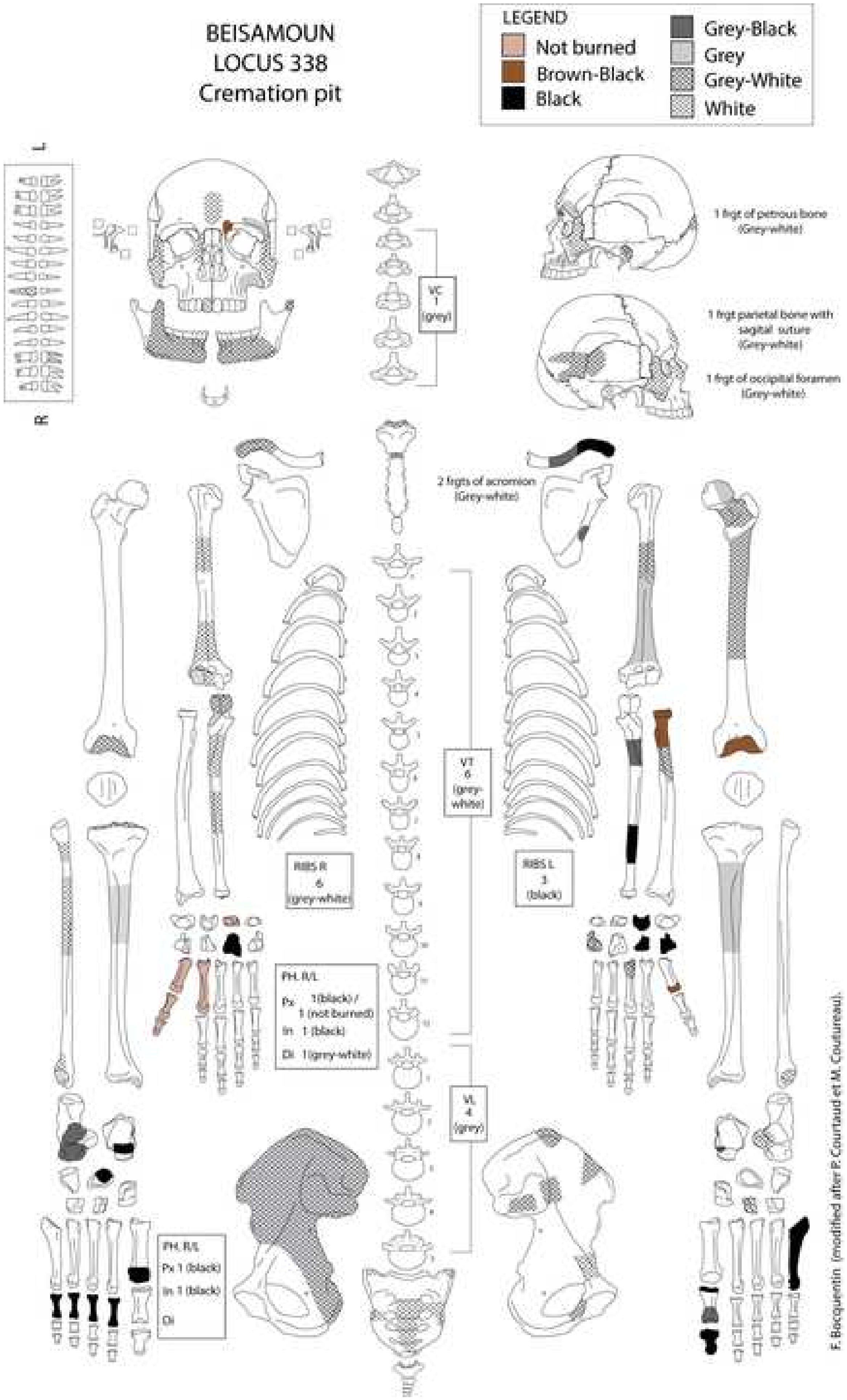

Click here to download Figure Fig5.tif

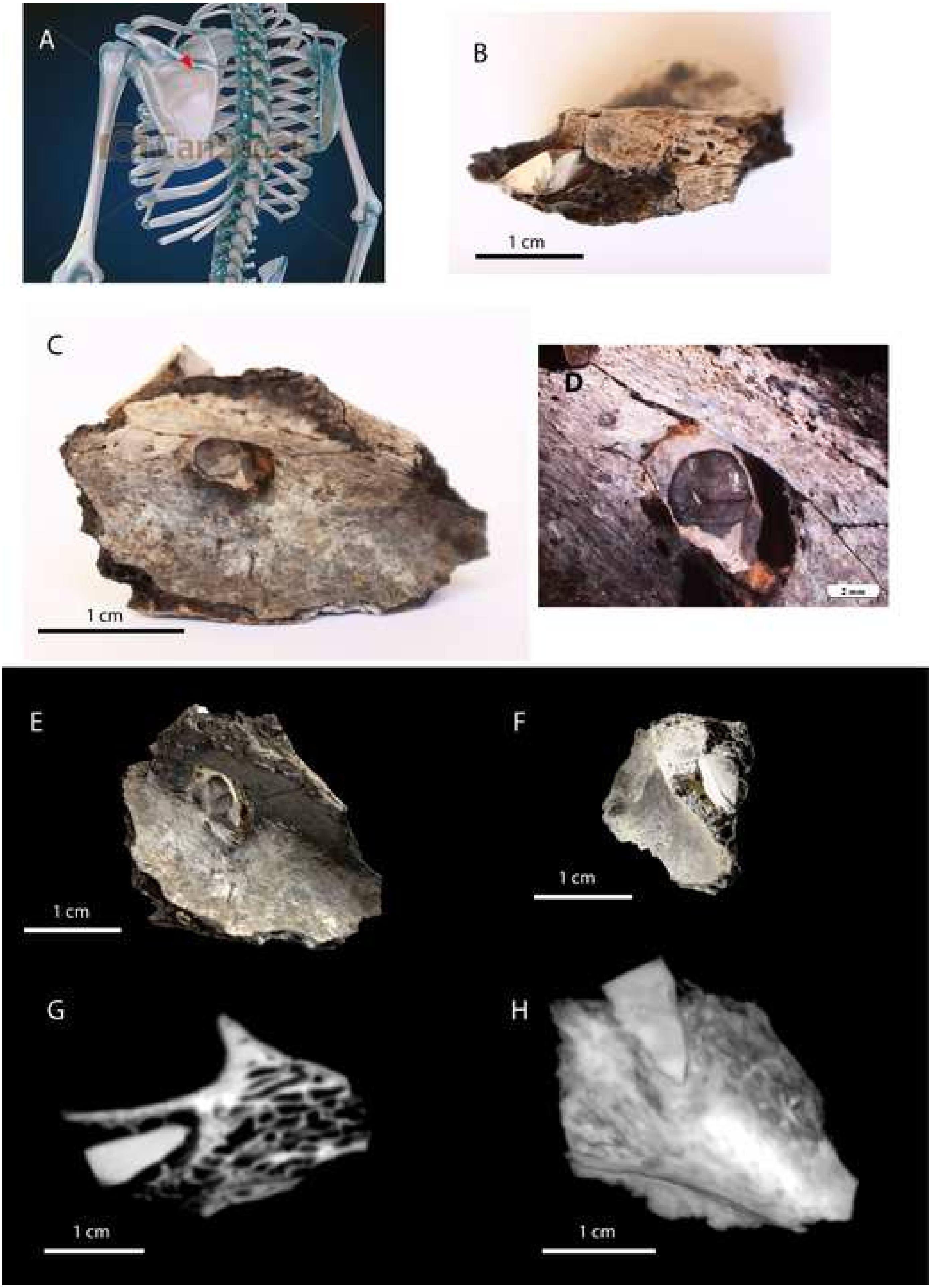

Click here to download Figure Fig6.tif

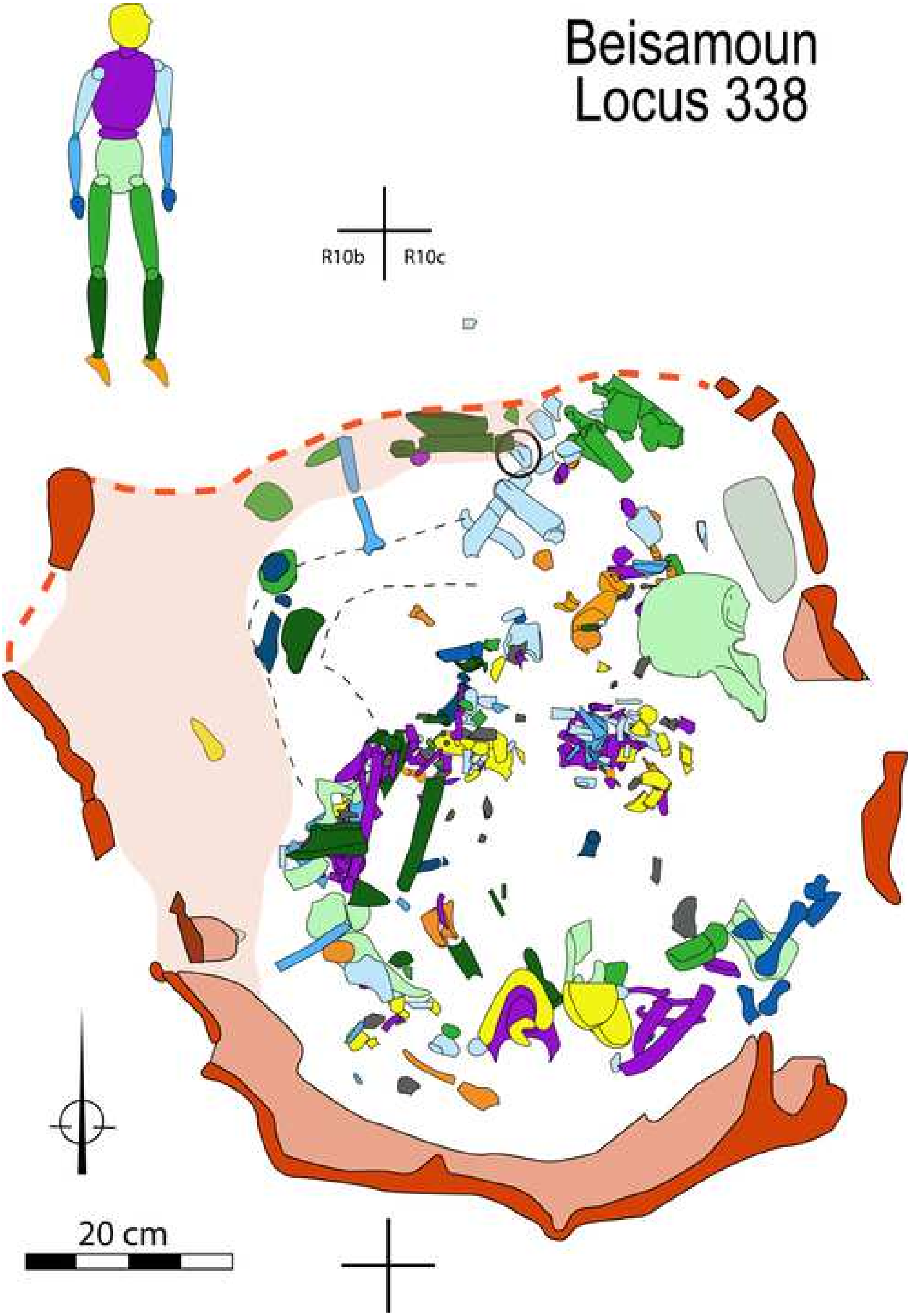

Click here to download Figure Fig7.tif

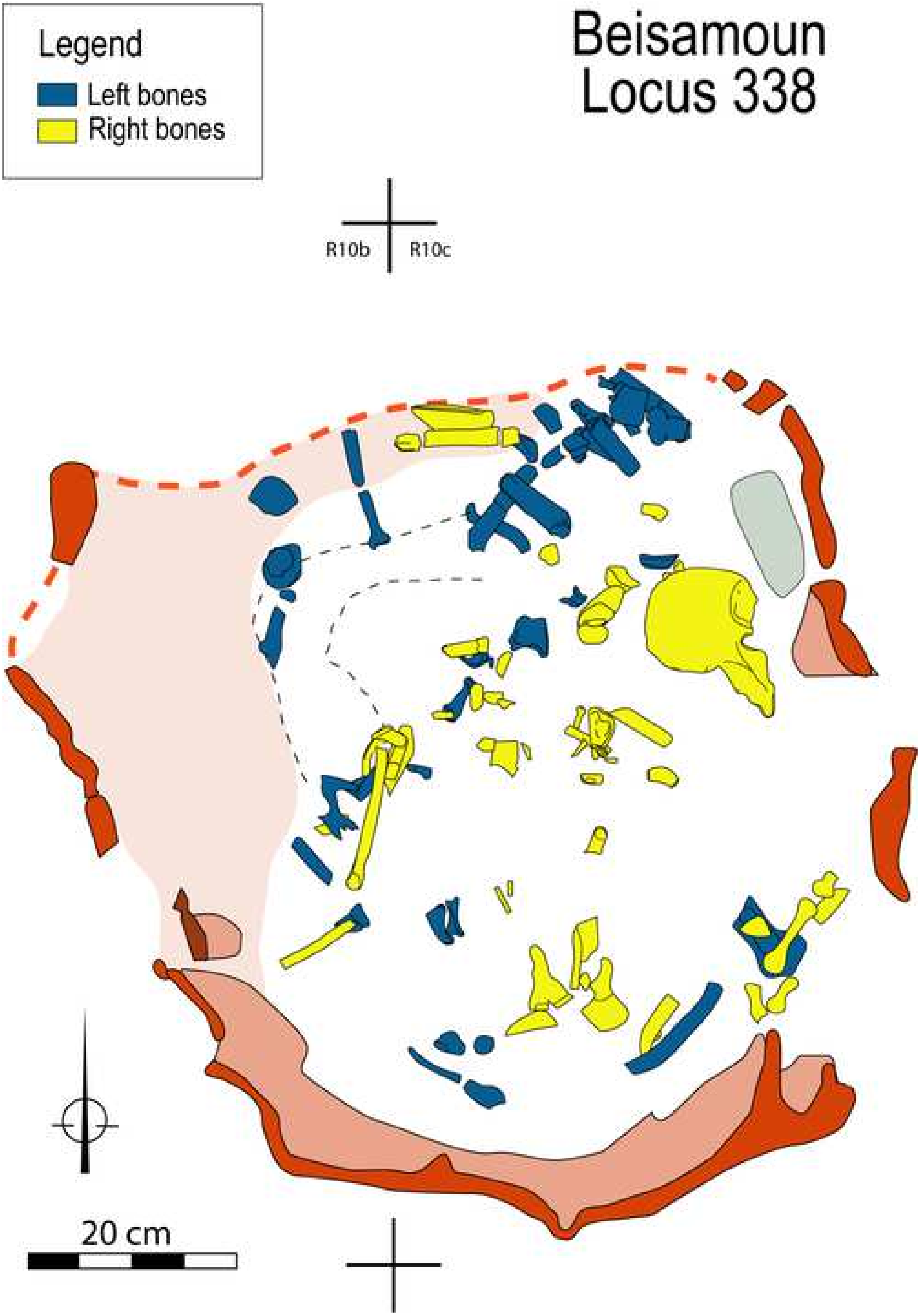

Click here to download Figure Fig8.tif

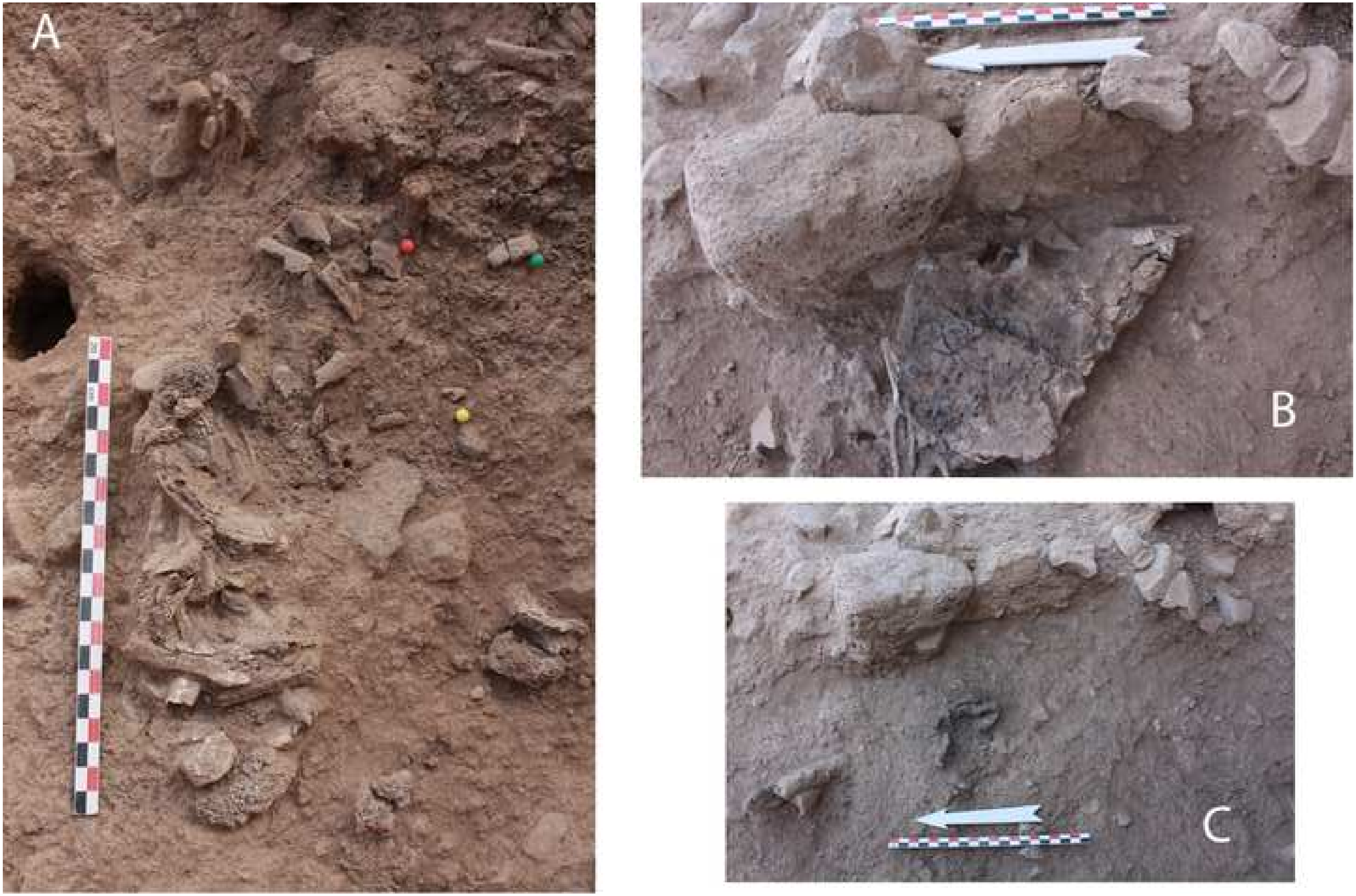

Click here to download Figure Fig9.tif

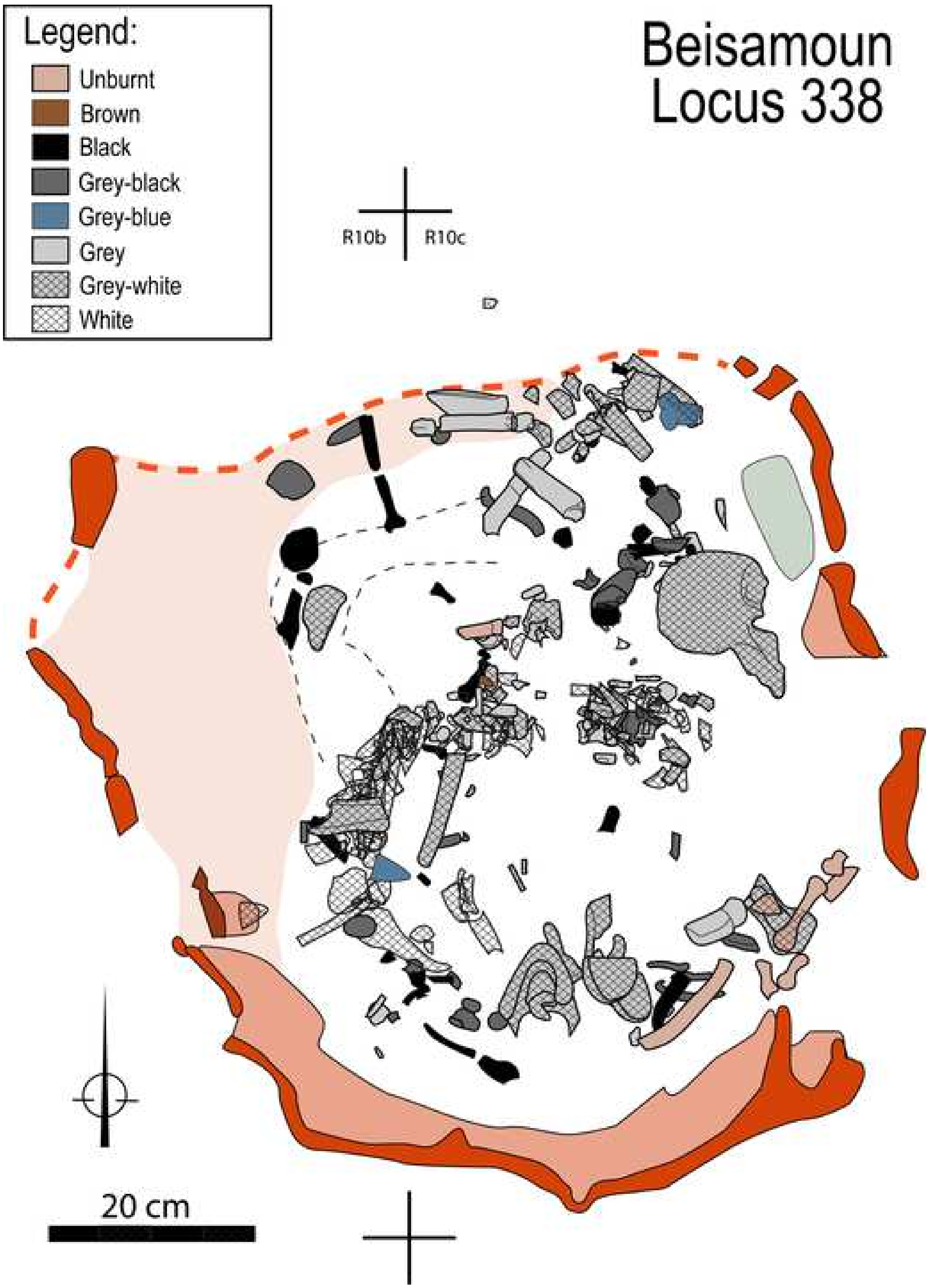

Click here to download Figure Fig10.tif

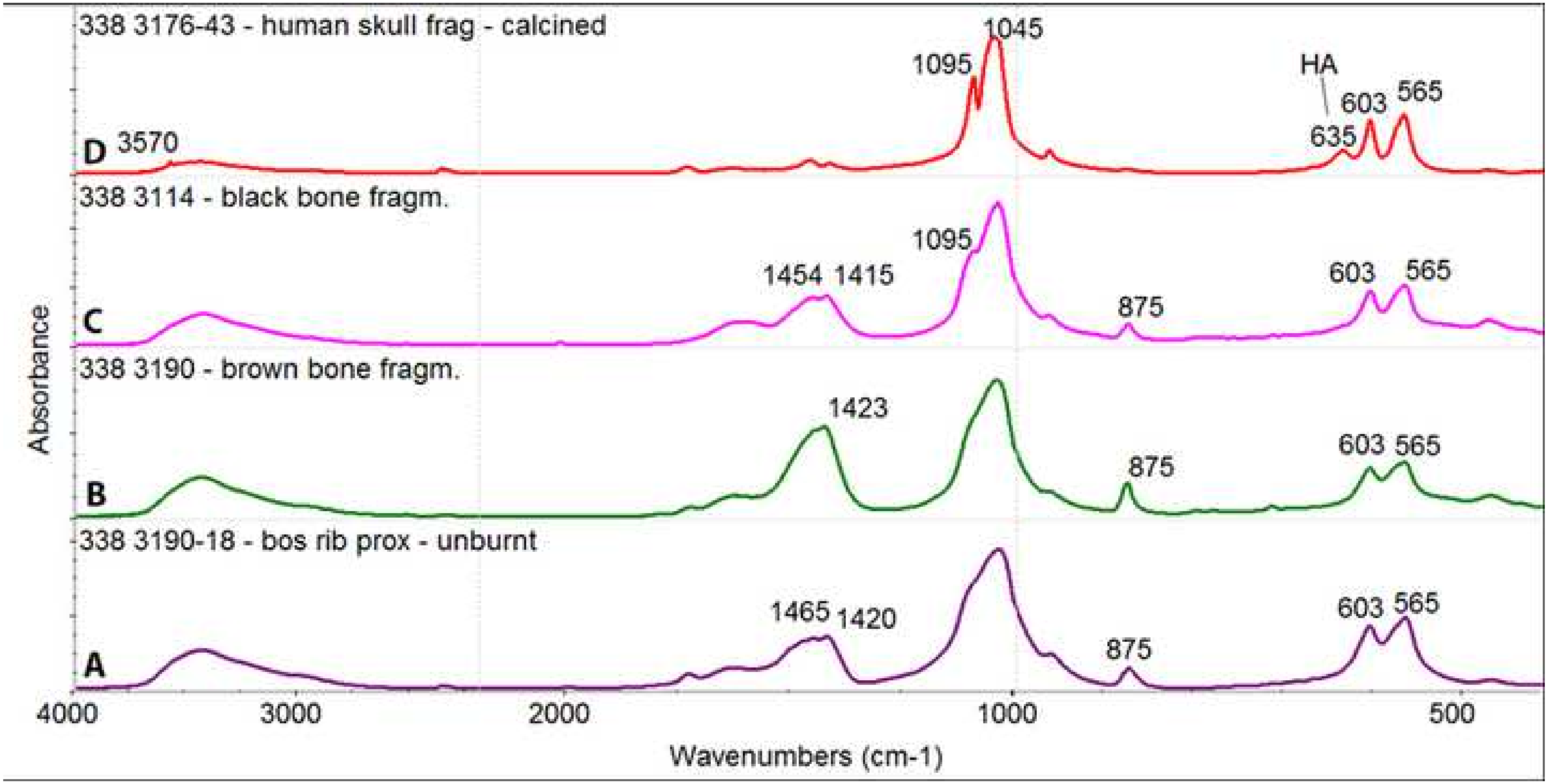

Click here to download Figure Fig11.tif

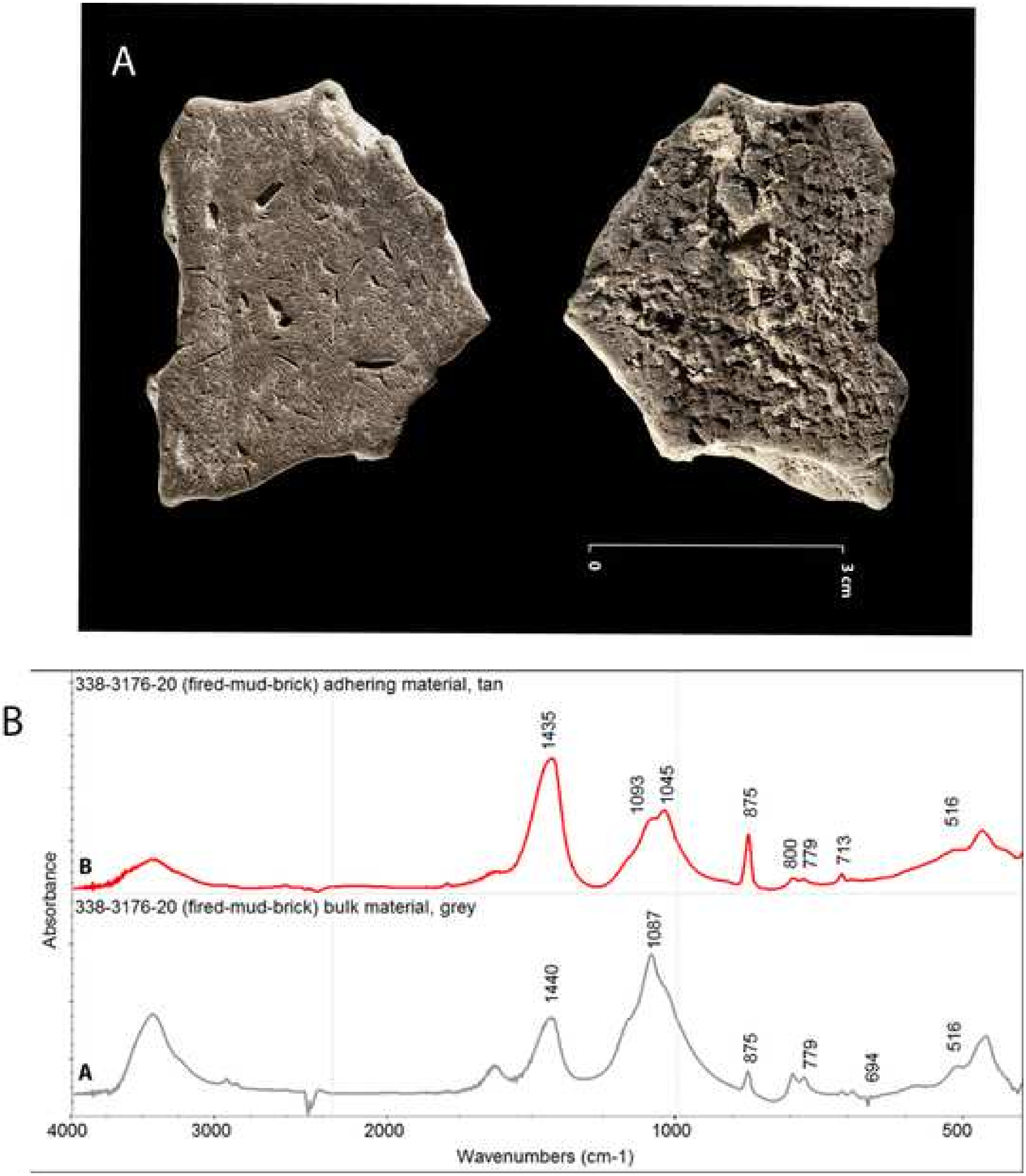

Click here to download Figure Fig12.tif

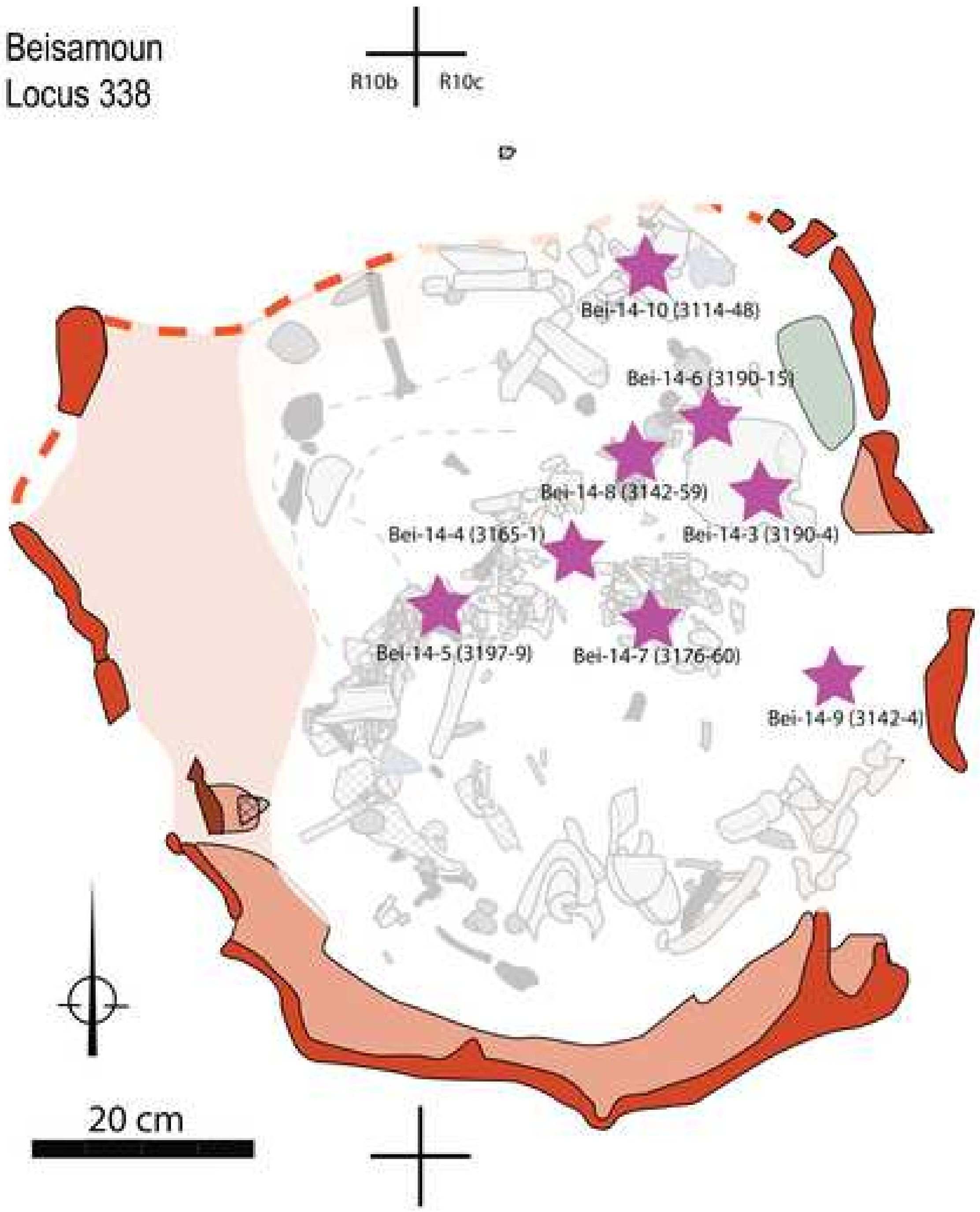

Lab No.	Square or Locus	Sample	Context	Heights
**Bei-14-1**	R10a	2885-1	patch of harder sediment	-45/-47
**Bei-14-2**	S9b	3028-1	burnt sediment	-43/-45
**Bei-14-3**	Locus 338	3190-4	under complete coxae #3176-32	-78
**Bei-14-4**	Locus 338	3165-1	block of ashes in the middle of the pit	-76
**Bei-14-5**	Locus 338	3197-9	burnt sediment found at the bottom of the cremation pit	-86
**Bei-14-6**	Locus 338	3190-15	under 4 foot phalanges in anatomical connexion #3190-6,7,8,16	-78
**Bei-14-7**	Locus 338	3176-60	under bones #3176_43,45,54	-75
**Bei-14-8**	Locus 338	3142-59	in contact with bone #3142-58	-76
**Bei-14-9**	Locus 338	3142-4	ashy sediment, next to eastern wall of the pit and bone #3142-40	-75
**Bei-14-10**	Locus 338	3114-48	north of pit and under bones #3114-8,10,11	-77

Click here to download Figure Fig13.tif

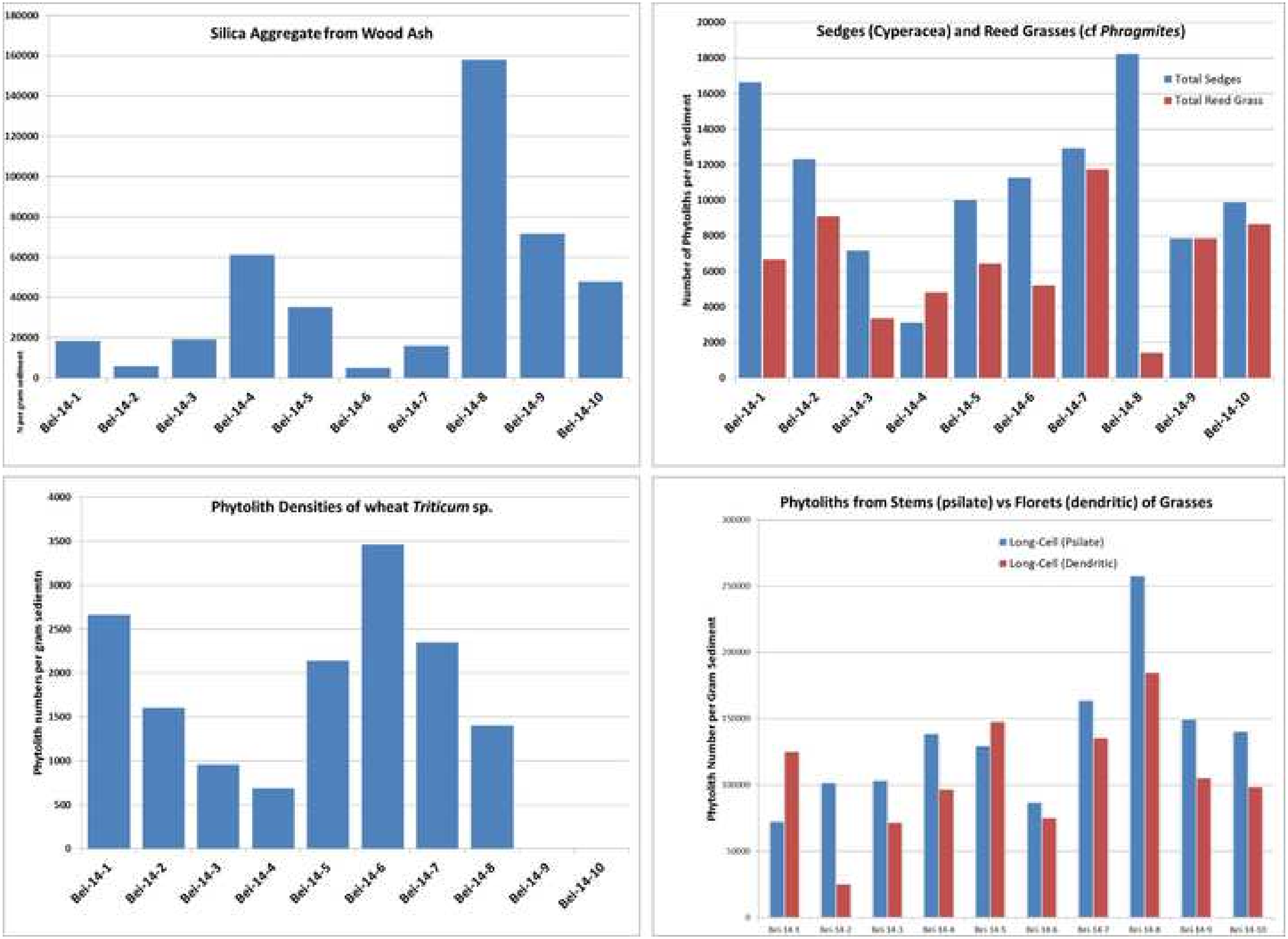

Click here to download Figure Fig14.tif

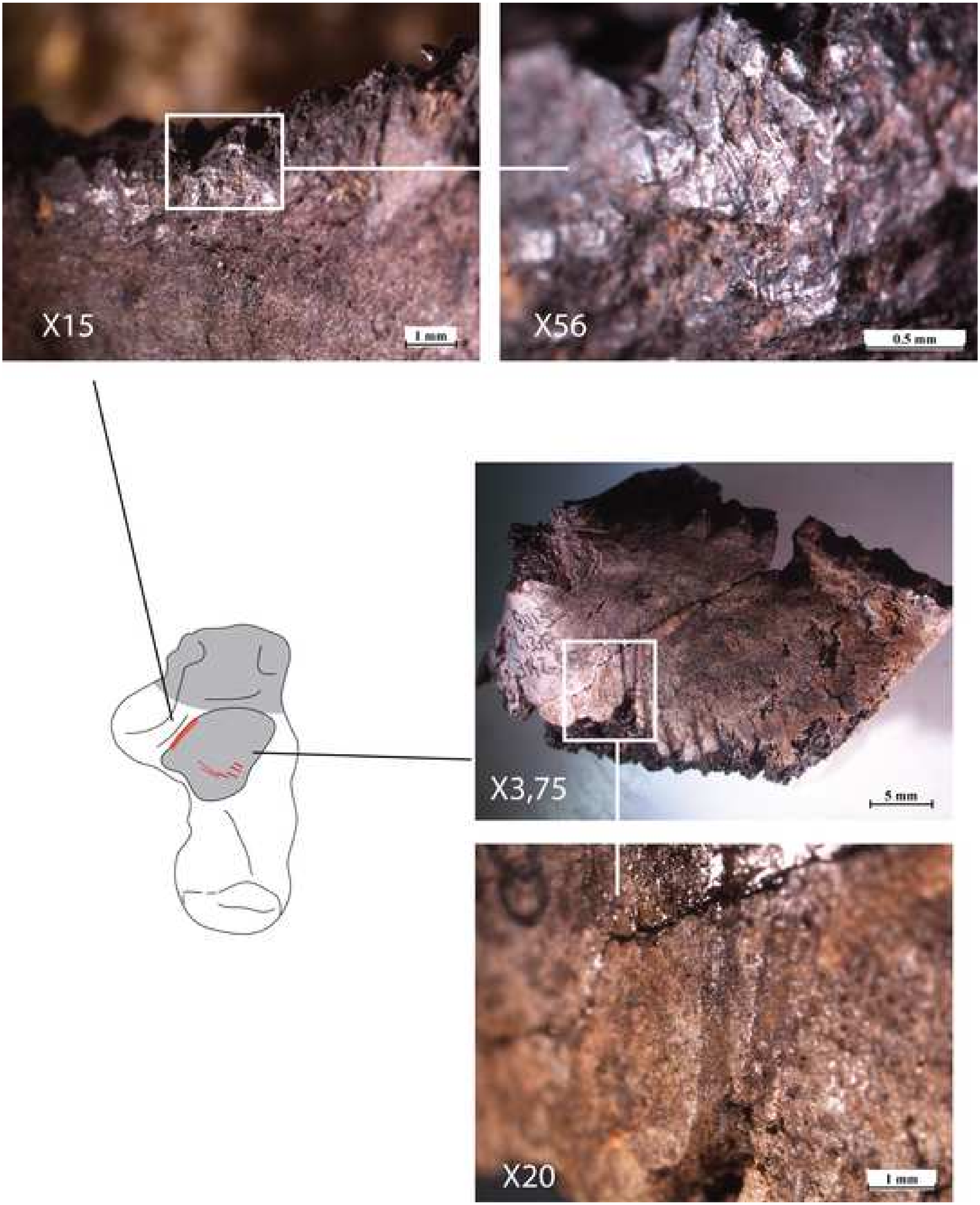

Click here to download Figure Fig15.tif

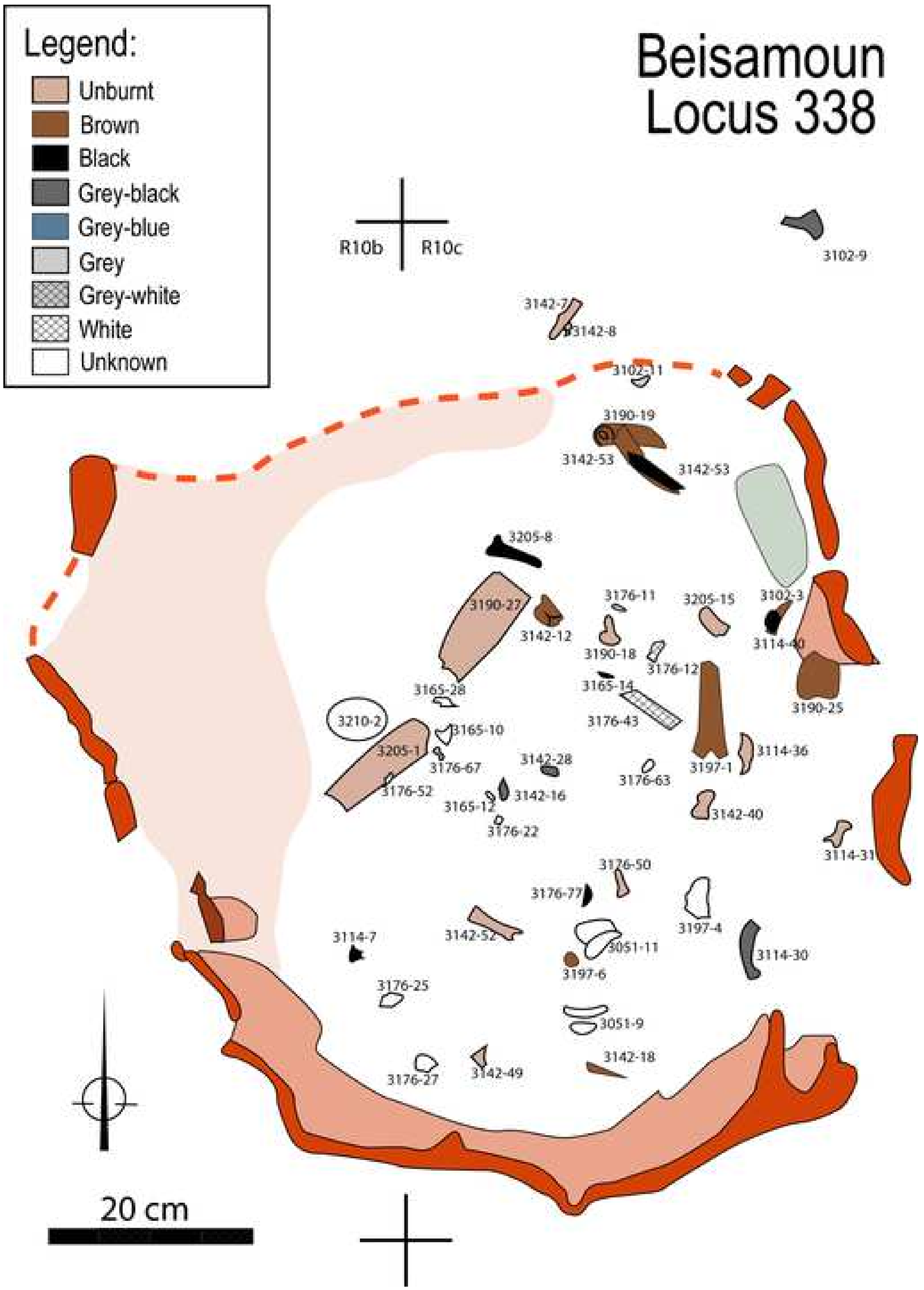

Click here to download Figure Fig16.tif

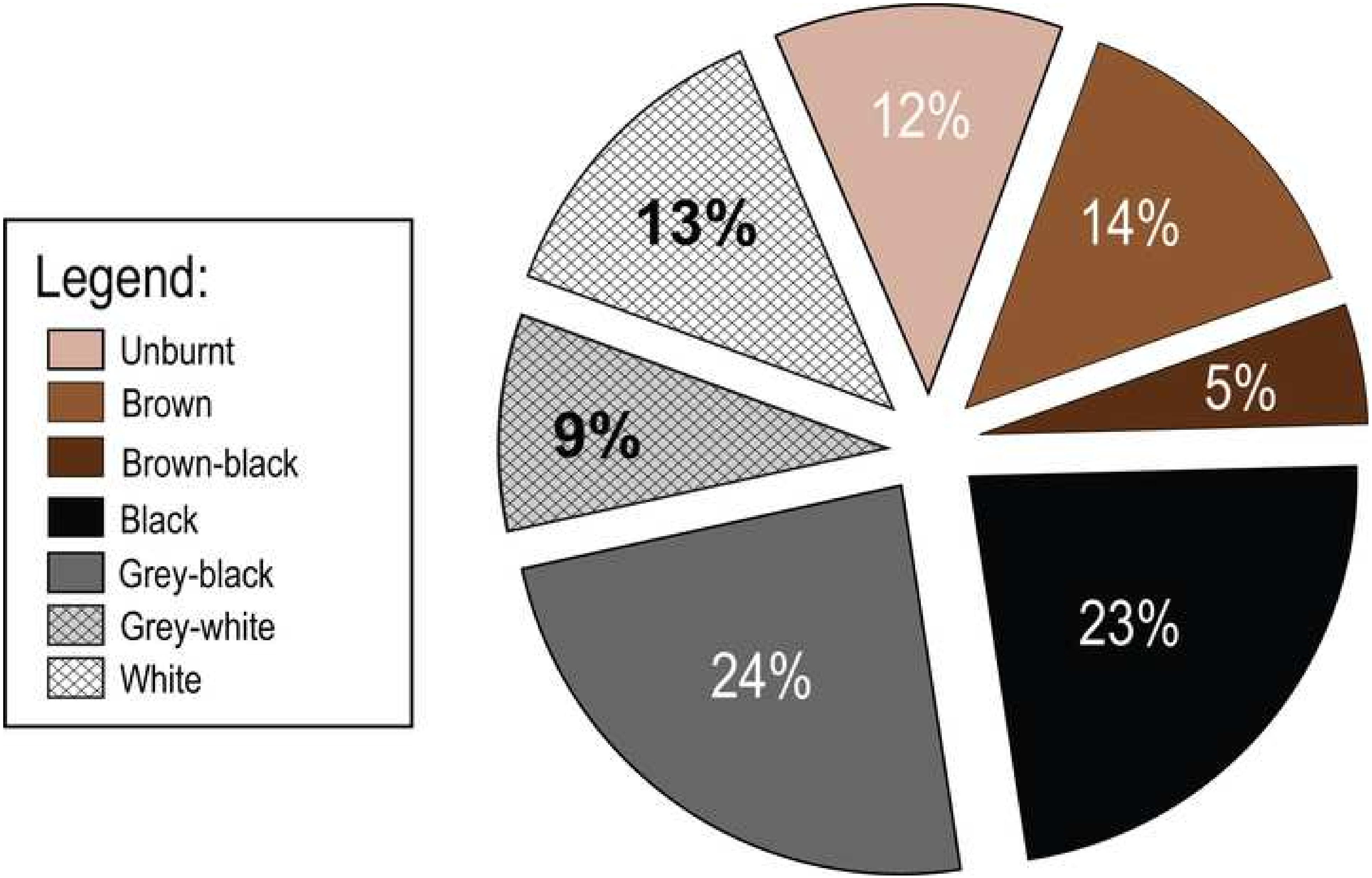

Click here to download Figure Fig17.tif

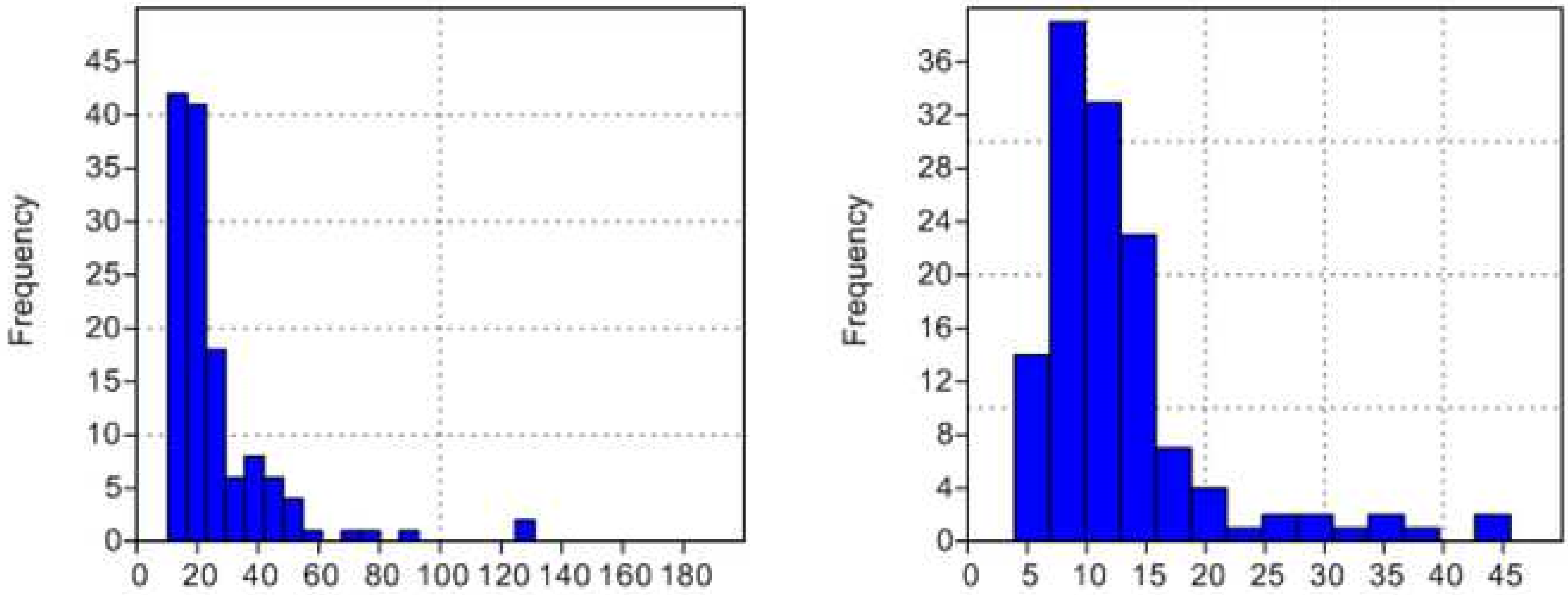

Click here to download Figure Fig18.tif

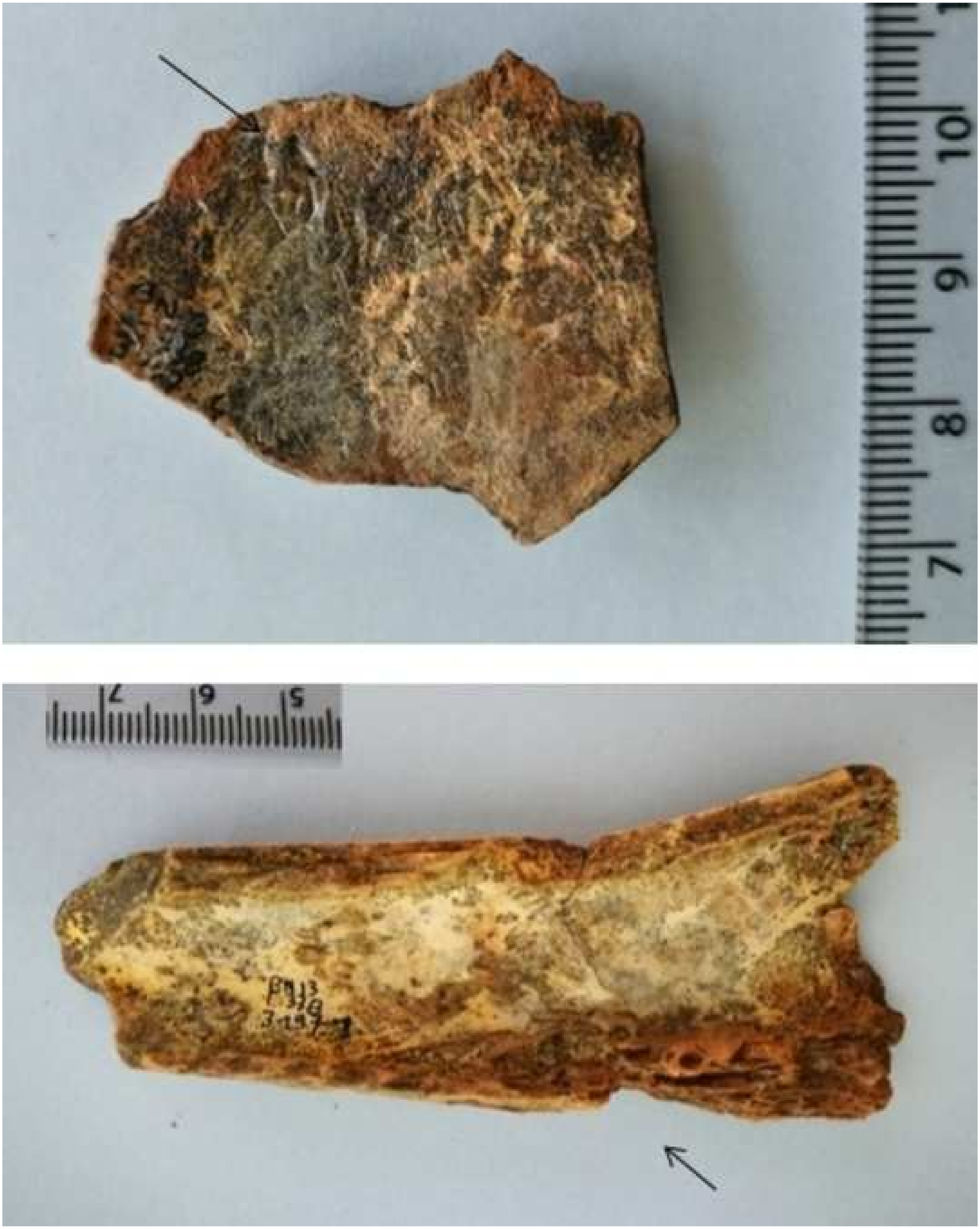

Click here to download Figure Fig19.tif

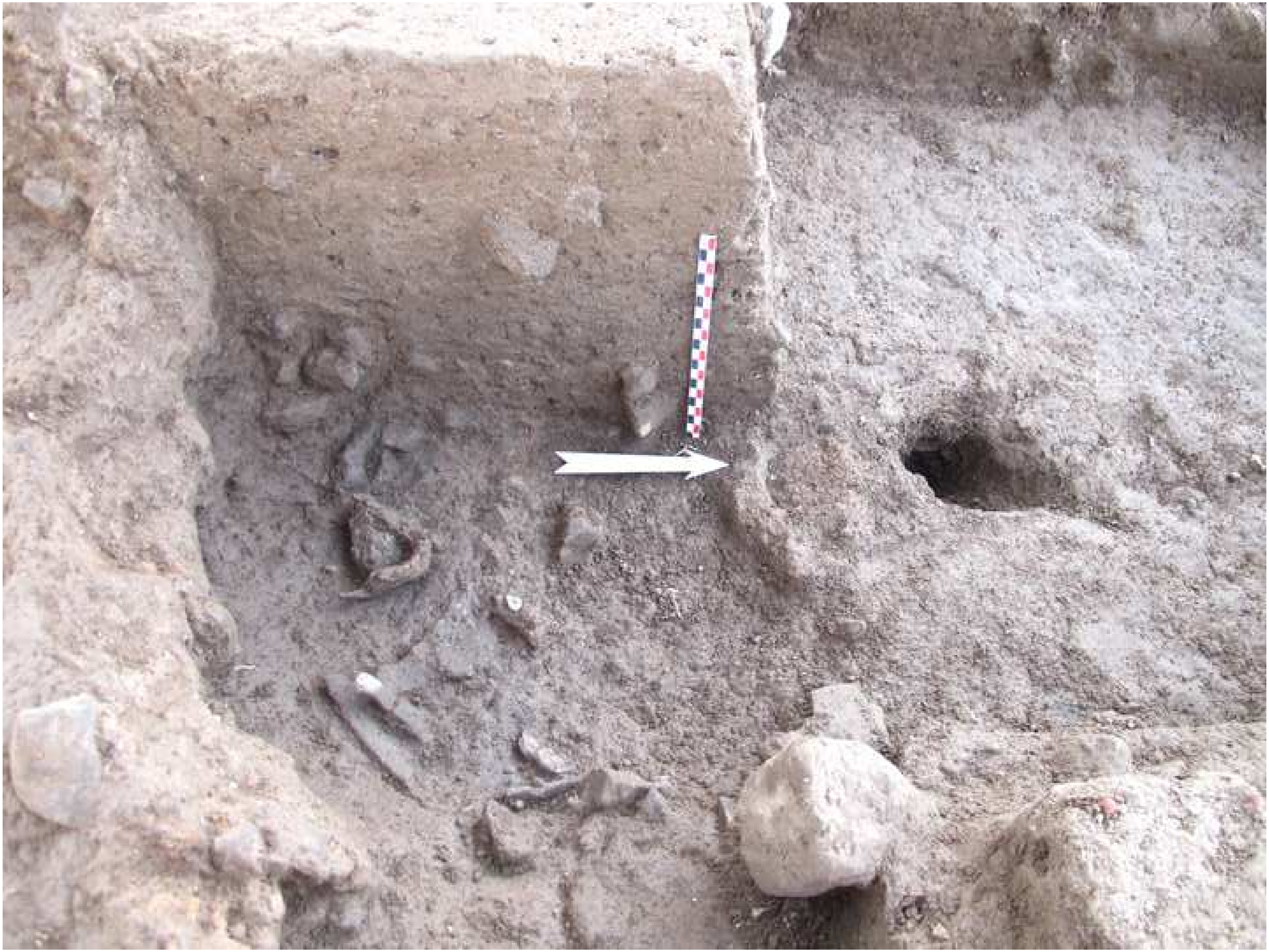

Click here to download Figure Fig20.tif

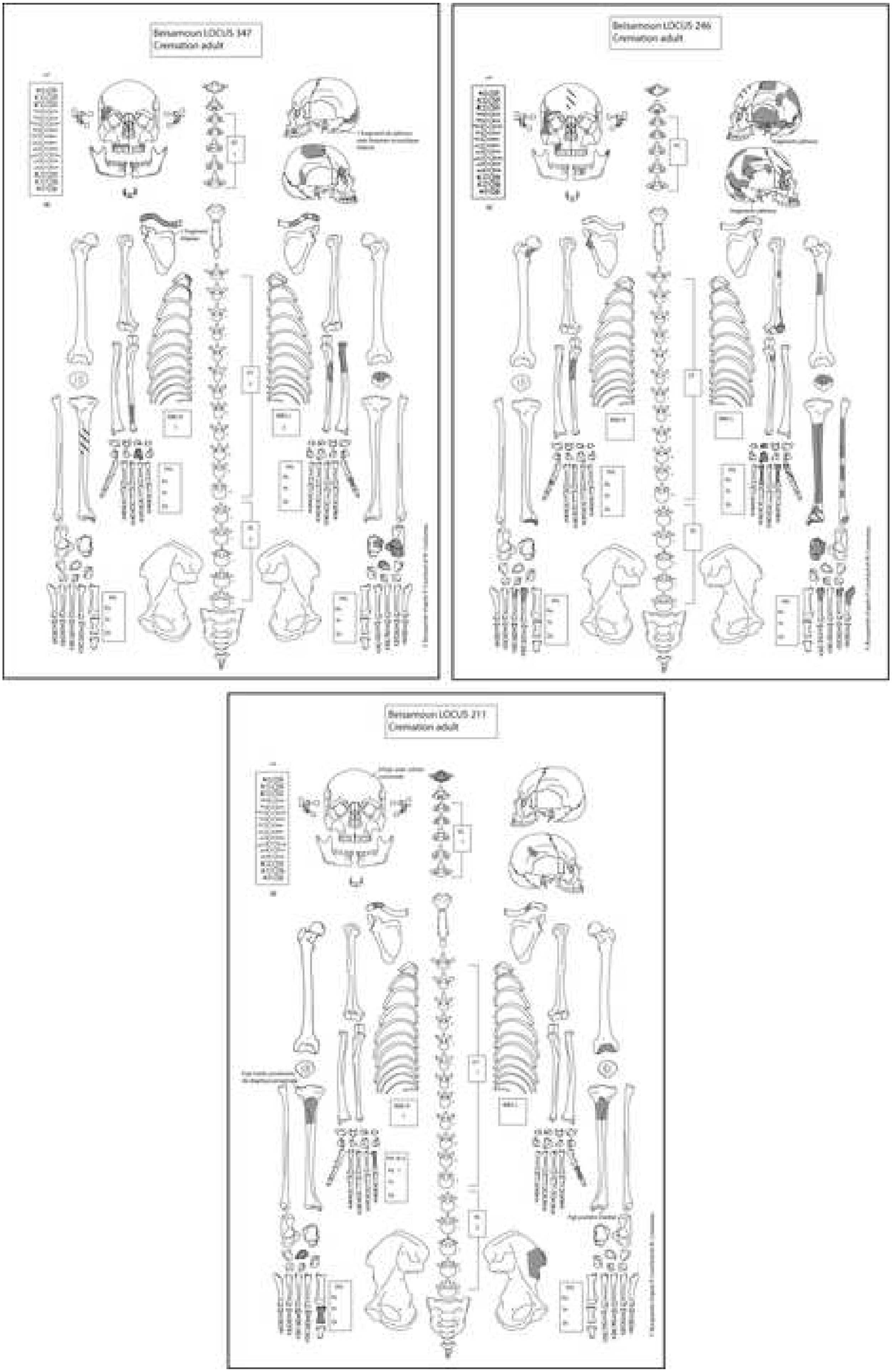

Supplement: Attachment — Submitted filename: PONE-D-19-25674_reviewer -ANSWERS IN CAPS.pdf [file pone.0235386.s004.pdf]
