# Supplementary material for: Emergence of corpse cremation during the Pre-Pottery Neolithic of the Southern Levant: A multidisciplinary study of a pyre-pit burial
Source: PLoS One. 2020 Aug 12;15(8):e0235386. doi: 10.1371/journal.pone.0235386 (PMC7423105; doi:10.1371/journal.pone.0235386)
Supplement: Attachment — Submitted filename: Response to Reviewers-bocquentin et al.docx [file pone.0235386.s005.docx]

**'Response to Reviewers'**

PONE-D-19-25674R1
Emergence of corpse cremation during Pre Pottery Neolithic in the Southern Levant
PLOS ONE

Please find in green our responses to each reviewers.

Reviewer #1: The paper is considerably improved in its current version and I still believe this is an important and generally well-documented piece of research. However, I am disappointed that the authors persist in playing down or ignoring entirely the increasing evidence from other sites in the Neolithic Near East for delayed burial (involving either complete desiccation of the corpse or some form of soft tissue reduction, i.e. partial decomposition), especially in the discussion section where the last set of comments were completely ignored.

Regarding desiccation, under the right environmental conditions, or with intentional effort, human remains can desiccate in parts of the world that would not be considered "deserts". There are ethnographic examples from Papua New Guinea and other tropical environments (where corpses are smoked), for example, and even within Mediterranean coastal regions such as Sicily, where the well-known Capuchin mummies at Palermo desiccated naturally.

We have added a whole paragraph dedicated to that topic. We argue that the hypothesis of a phase of desiccation before cremation is unlikely but we keep the discussion open.

As with reviewer #2, I am skeptical about the feasibility of successfully cremating a fresh corpse in such a small and poorly ventilated space - both in terms of fuel load required and proper ventilation (regardless of whether or not the pit opening was left uncovered). I would find their argument more convincing if they acknowledged the possibility that the corpse may have been already partially decomposed, as it would likely require less fuel; proper ventilation may have been less of an issue as well, and would still accommodate the observation that joint articulations seem to have been maintained. They use the term "relatively fresh corpse", but perhaps they need to define what they mean by "relatively". It may be that we, as reviewers, and the authors are not so far apart after all on the possibility of a period of delay between death and cremation?

Again, we've added a paragraph to discuss the feasibility of cremation in a relatively narrow pit. We can see that this case is far to be unique in the literature and that several technical possibilities are being considered to understand how these pyre-pits work.

Regarding data availability (question 4 above), as mentioned in my comments on the first draft, it would have been nice to see more images of the burnt bones themselves so that one could independently assess the level of heat alterations to bone in the context of differences between dry and fleshed bone. I do understand however that providing images of every single bone is not feasible.

We have added a figure (Fig 10) with about twenty bones illustrating the different hues of colour found on the cremated bones of Locus 338.

Reviewer #2: Author: Fanny Bocquentin
Title: Emergence of Corpse Cremation during the Southern Levantine Pre Pottery Neolithic
Journal: PLOS-ONE
File number: PONE-D-1925674

Rec: Accepted with minor revisions.

This is now an excellent paper, and should be published with minor revisions. The authors have made a good effort to address my concerns with the original version. I am supportive of the publication of this paper subject to addressing the issues raised below.

Reviewer 2: Ian Kuijt

General comments
First, while this is a great methodological paper, the paper suffers from having a very poor introduction. The introduction meanders and does not really tell the readers what the main focus of the paper will be….and then the paper kind of gets lost in history and symbolism. The authors need to identify the main focus of the paper. Is it strictly focused on demonstrating that this earliest occurrence of intentional primary cremation from the Near East? If so, this is a wasted opportunity. This is excellent research, well-illustrated and presented, but the authors do not step back from the trees and talk about the forest. I urge the authors to think about the big issues they identify at the end of the paper, in some ways using these themes to introduce the paper. The introduction and abstract are the most important ways you can draw in the readers, so these need to be crisp, focused on major evolutionary issues, and digestible to the readers.

Second, the paper does a poor job of defining the dates of the periods in question. For example, the PPNC / final PPNB is mentioned on page one, but not with specific details. It is mentioned in the abstract, but this also needs to be in the body of the text.

Third, I would urge the authors to go back and add a sub-heading to introduce the site of Beisamoun and reorganize the section Chrono-Cultural Context. I think the original was more effective. Right now the site is introduced in a hidden way, buried in the middle of the section titled Chrono-Cultural Context. This is a poorly developed section, with the title hinting at a discussion of regional culture-history, and then in the middle of this section we are introduced to Beisamoun.

We have completely rewritten or reorganized the summary, the introduction, the contextual part and the conclusion. We have added information about the C14 dates.

Fourth, the authors have added a much needed section on excavation methods (sections 2.1., 2.2.). These are excellent and really, really, make this an outstanding paper.

Fifth, section 2.6.3. deals with the burning event. This is a good section. My only suggestion is to add more clarity, if you can, about the placement of the wood and body. You talk about fuel and other great topics, but I am struggling to understand what you see as the original position of the fuel and body. For example, do you believe that the 60 cm deep pit was filled with wood, up to and above the ground surface, and then the fire was started? Given the need for oxygen this makes sense, for it is all about oxygen. In another section you state that the person may have been seated in the 60 cm high pit. If so, was fuel placed around the body? If this is your argument then place find a place, probably as the lead sentence of one of your paragraphs to state this up front. Help you reader understand your overall view, and then dive into the details.

As specified for reveiwer 1, we have added a paragraph on this subject to discuss the different technical possibilities. We cannot give a definitive answer because the functioning of a pyre through the preserved material remains is always extremely difficult, especially for this case of pyre-pit which is one of the oldest in the world.

Specific comments:
Fig. 1. The names of multiple sites are misspelled on the map and need to be corrected.
Tell el-Kerkh is the correct published spelling, not Tell-el-Kherk as listed on Fig. 1.
Yarim Tepe II is the correct published spelling, not Yarim tepe II as listed on Fig. 1
‘Ain Ghazal is the correct published spelling, not Aïn Ghazal as listed on Fig. 1. Sorry, but M. Sauvage, CNRS, needs to correct this figure before publication.

Pg. 2., first sent, first paragraph. “The treatment of the dead during the Neolithization process in the Near East was a complex process, part of the cognitive and symbolic world that guided the economic and dietary shift from hunting and gathering to agro-pastoralism [e.g. articles in 1].” This suggests that the treatment of the dead “guided” the shift from hunting and gathering to agro-pastoralism. I would urge the authors to reconsider this argument, or perhaps it is a misstatement.

Pg. 3., first paragraph “…as the condemnation of the funeral building itself by fire.” This is poor wording. Might I suggest “the burning of the funerary building”. Condemnation is not a clear way to present this.

Pg. 3., first paragraph, and throughout the entire paper, “…site of Tell Ain el Kherk in a …”. As correctly cited in the references, the name of this site is Tell el-Kerkh (see Tsuneki 2012). To meet the publication standards of this journal it is critical that the authors employ the correct, published spelling of all sites in the text, references and figures.

All have been addressed

Pg. 4., end of second paragraph “They are currently kept at the French National
Research Center in Jerusalem (CRFJ) and they will be handed over to the IAA after publication. In the meantime, they are available for review upon request to the CRFJ and the corresponding author of the current article.” This should be in a foot or endnote. This does not advance discussion: it is an administrative aside.

moved to footnote

Pg. 4., first sentence of section 2.1. “As for all features…”. Should be corrected to “As with all features….”

The paper reads well, but as with all essays at this point of production, there are typos and grammatical issues that need to be cleared up before publication.
We hope we've corrected everything
